# Immunogenicity and Risk Factors Associated With Poor Humoral Immune Response of SARS-CoV-2 Vaccines in Recipients of Solid Organ Transplant: A Systematic Review and Meta-Analysis

**DOI:** 10.1001/jamanetworkopen.2022.6822

**Published:** 2022-04-12

**Authors:** Kasama Manothummetha, Nipat Chuleerarux, Anawin Sanguankeo, Olivia S. Kates, Nattiya Hirankarn, Achitpol Thongkam, M. Veronica Dioverti-Prono, Pattama Torvorapanit, Nattapong Langsiri, Navaporn Worasilchai, Chatphatai Moonla, Rongpong Plongla, William M Garneau, Ariya Chindamporn, Pitchaphon Nissaisorakarn, Tany Thaniyavarn, Saman Nematollahi, Nitipong Permpalung

**Affiliations:** 1 Department of Microbiology, Faculty of Medicine, Chulalongkorn University, Bangkok, Thailand; 2 Department of Physiology, Faculty of Medicine, Chulalongkorn University, Bangkok, Thailand; 3 Department of Preventive and Social Medicine, Faculty of Medicine Siriraj Hospital, Mahidol University, Bangkok, Thailand; 4 Department of Medicine, Johns Hopkins University School of Medicine, Baltimore, Maryland; 5 Department of Medicine, Faculty of Medicine, Chulalongkorn University, Bangkok, Thailand; 6 King Chulalongkorn Memorial Hospital, Bangkok, Thailand; 7 Faculty of Allied Health Sciences, Chulalongkorn University, Bangkok, Thailand; 8 Department of Medicine, Massachusetts General Hospital, Boston; 9 Department of Medicine, Brigham and Women’s Hospital, Boston, Massachusetts; 10 Department of Medicine, University of Arizona College of Medicine, Tucson

## Abstract

**Importance:**

Recipients of solid organ transplant (SOT) experience decreased immunogenicity after COVID-19 vaccination.

**Objective:**

To summarize current evidence on vaccine responses and identify risk factors for diminished humoral immune response in recipients of SOT.

**Data Sources:**

A literature search was conducted from existence of database through December 15, 2021, using MEDLINE, Embase, Web of Science, Cochrane Library, and ClinicalTrials.gov.

**Study Selection:**

Studies reporting humoral immune response of the COVID-19 vaccines in recipients of SOT were reviewed.

**Data Extraction and Synthesis:**

Two reviewers independently extracted data from each eligible study. Descriptive statistics and a random-effects model were used. This report was prepared following the Preferred Reporting Items for Systematic Reviews and Meta-analyses (PRISMA) reporting guideline. Data were analyzed from December 2021 to February 2022.

**Main Outcomes and Measures:**

The total numbers of positive immune responses and percentage across each vaccine platform were recorded. Pooled odds ratios (pORs) with 95% CIs were used to calculate the pooled effect estimates of risk factors for poor antibody response.

**Results:**

A total of 83 studies were included for the systematic review, and 29 studies were included in the meta-analysis, representing 11 713 recipients of SOT. The weighted mean (range) of total positive humoral response for antispike antibodies after receipt of mRNA COVID-19 vaccine was 10.4% (0%-37.9%) for 1 dose, 44.9% (0%-79.1%) for 2 doses, and 63.1% (49.1%-69.1%) for 3 doses. In 2 studies, 50% of recipients of SOT with no or minimal antibody response after 3 doses of mRNA COVID-19 vaccine mounted an antibody response after a fourth dose. Among the factors associated with poor antibody response were older age (mean [SE] age difference between responders and nonresponders, 3.94 [1.1] years), deceased donor status (pOR, 0.66 [95% CI, 0.53-0.83]; *I*^2^ = 0%), antimetabolite use (pOR, 0.21 [95% CI, 0.14-0.29]; *I*^2^ = 70%), recent rituximab exposure (pOR, 0.21 [95% CI, 0.07-0.61]; *I*^2^ = 0%), and recent antithymocyte globulin exposure (pOR, 0.32 [95% CI, 0.15-0.71]; *I*^2^ = 0%).

**Conclusions and Relevance:**

In this systematic review and meta-analysis, the rates of positive antibody response in solid organ transplant recipients remained low despite multiple doses of mRNA vaccines. These findings suggest that more efforts are needed to modulate the risk factors associated with reduced humoral responses and to study monoclonal antibody prophylaxis among recipients of SOT who are at high risk of diminished humoral response.

## Key Points

**Question:**

What are the humoral immune response rates and risk factors associated with diminished response after COVID-19 vaccination in recipients of solid organ transplant?

**Findings:**

In this systematic review and meta-analysis of 29 studies and 11 713 recipients of solid organ transplant, seroconversion rates increased with progressively increased numbers of mRNA COVID-19 vaccine doses. Older age, recent transplantation, deceased donor status, active use of antimetabolites, and recent exposure to antithymocyte globulin or rituximab were risk factors associated with diminished humoral immune response after receiving 2 doses of mRNA vaccines.

**Meaning:**

These findings suggest that more efforts are needed to modulate the risk factors associated with reduced humoral responses among recipients of solid organ transplant.

## Teaser

This systematic review and meta-analysis assesses the current evidence on vaccine response and risk factors associated with diminished immune response in recipients of solid organ transplant.

## Introduction

Individuals with COVID-19 who have undergone solid organ transplant (SOT) experience higher mortality and prolonged viral shedding compared with the general population.^1,2,3,4,5^ Multiple vaccine platforms have been proven successful in reducing viral spread and preventing poor outcomes in the general population.^6,7,8^ Unfortunately, recipients of SOT were excluded from the initial licensing trials of these vaccines, and accumulating data have shown reduced immunogenicity among recipients of SOT.^9,10,11,12,13,14^

The US Food and Drug Administration (FDA) has approved the COVID-19 mRNA vaccines BNT162b2 (Pfizer-BioNTech) and mRNA-1273 (Moderna) and has granted emergency use authorization (EUA) for the adenoviral vector vaccine Ad26.COV2.S (Janssen).^15,16,17,18^ In response to emerging SARS-CoV-2 variants and evidence of a mortality benefit from booster or additional doses, the US Centers for Disease Control and Prevention (CDC) recommended a booster or additional dose after completion of the primary COVID-19 vaccination series for all adults who received BNT162b2, mRNA-1273, or Ad26.COV2.S.^19,20,21^ For patients who are immunocompromised, including recipients of SOT, the CDC recommended an additional primary shot (third dose of mRNA COVID-19 vaccine for those receiving BNT162b2 or a booster dose of mRNA-1273) and a subsequent dose (fourth dose of BNT162b2 or second booster dose of mRNA-1273 for those receiving mRNA COVID-19 vaccine or second dose for those receiving Ad26.COV2.S).^22^ Despite this strategy, there are concerns for inadequate protection and risks of breakthrough infections among recipients of SOT because of diminished immunogenicity. We conducted this systematic review and meta-analysis to summarize the current evidence on vaccine responses and identify risk factors associated with diminished humoral immune response among recipients of SOT.

## Methods

### Data Sources and Searches

A systematic search was conducted independently by 2 of us (N.C. and K.M.) in MEDLINE, Embase, Web of Science (Clarivate), Cochrane Library, and ClinicalTrials.gov databases for research available through December 15, 2021. Complete search terms are included in the eMethods in the Supplement. Studies from different databases were combined, and duplicates were excluded. We did not limit our search by language. We conducted the study according to Preferred Reporting Items for Systematic Reviews and Meta-analyses (PRISMA) reporting guideline. This study was registered in the International Prospective Register of Systematic Reviews (PROSPERO) (CRD42021277109).

### Study Selection and Quality Assessment

Two authors (N.C. and K.M.) independently reviewed all studies and selected studies that reported the immunogenicity of COVID-19 vaccines in recipients of SOT, described as study participants in the methods and results. We included clinical trials and observational studies consisting of prospective cohort, retrospective cohort, and case-control studies. We excluded studies of humoral immunity after COVID-19 infection in study participants. Corresponding authors were contacted for immunogenicity testing or vaccination information if needed. We used Google Translate (Alphabet) to translate non-English studies during title and abstract screening. The Newcastle-Ottawa scale was used for assessing the risk of bias of the studies (eTable 1 in the Supplement).^23^ Conflicts were resolved by mutual consensus between reviewers.

### Data Extraction

The checklist for critical appraisal and data extraction for systematic reviews of prediction modeling studies was used.^24^ Our primary outcome was the seroconversion rate after COVID-19 vaccine administration. We extracted the numbers of responders and total participants to calculate the seroconversion rates. Responders were defined as participants whose humoral response met definitions and cutoffs of antibody testing in each primary study. The numbers of responders, total participants, and odds ratios (ORs) with 95% CIs of factors associated with vaccine response were extracted. If ORs were not available, crude numbers were extracted for OR calculation.

### Statistical Analysis

Descriptive statistics were used to characterize humoral immune response, the primary outcome, for each COVID-19 vaccine platform and for each number of doses. We then performed a meta-analysis with Comprehensive Meta-Analysis software version 3.3 (Biostat) to identify risk factors associated with poor humoral immune response. To determine the factors associated with humoral immunogenicity, pooled ORs (pORs) with 95% CIs for binary variables and differences in means (with SEs) for continuous variables were calculated using meta-analysis with the random-effects model. If the study provided both adjusted and unadjusted ORs, we used adjusted ORs for calculations. If the primary study provided ORs of the factors associated with vaccine nonresponse, we used log transformation to calculate ORs associated with vaccine response of those specific factors. We performed sensitivity analyses using a leave-1-out method.^25^ Funnel plot and Egger regression were used to assess the publication bias.^26^ If the *P* value of Egger regression was *P* < .1, the publication bias was considered significant.^27^ Factors with concerns of publication bias were further adjusted by the Duval and Tweedie trim-and-fill method.^28^ We assessed the heterogeneity of effect size estimates of each study using the *I*^2^ statistic. The *I*^2^ statistic ranged from 0% to 100%, with *I*^2^less than 25% indicating low heterogeneity; *I*^2^ of 25% to 60%, moderate heterogeneity; and *I*^2^ greater than 60%, substantial heterogeneity.^29^ *P* values were 2-sided, and statistical significance was set at *P* = .05. Data were analyzed from December 2021 to February 2022.

## Results

### Study and Patient Characteristics

**Figure 1.**
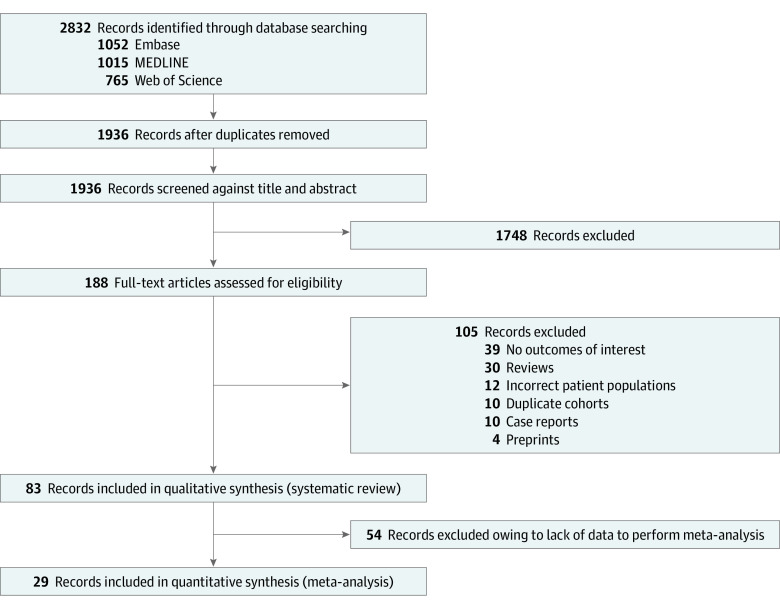
Study Selection Flowchart

Our initial search generated 2832 studies; 896 studies were removed because they were duplicates, and 1748 studies were excluded by screening through the titles and abstracts. We performed full-study reviews on 188 articles. After review, 105 articles were excluded owing to being a review article, case report, preprint, incorrect patient population, or duplicate cohort or having no outcomes of interest. A total of 83 studies^9,10,11,12,13,14,30,31,32,33,34,35,36,37,38,39,40,41,42,43,44,45,46,47,48,49,50,51,52,53,54,55,56,57,58,59,60,61,62,63,64,65,66,67,68,69,70,71,72,73,74,75,76,77,78,79,80,81,82,83,84,85,86,87,88,89,90,91,92,93,94,95,96,97,98,99,100,101,102,103,104,105,106^ were included in the systematic review, of which 29 studies were included in the meta-analysis (Figure 1). The characteristics of 83 included studies are described in eTable 2 in the Supplement. There were 11 713 study participants across all studies, including heart, lung, heart-lung, liver, kidney, pancreas, kidney-pancreas, and other combined transplantation. Grading of recommendation assessment, development and evaluation for potential factors associated with seroconversion was reported in eTable 3 in the Supplement.^107^

### Humoral Immune Responses

#### mRNA Vaccines

A total of 83 studies of immunogenicity of the mRNA COVID-19 vaccines in study participants were identified. Of these, 18 studies reported antibody response after 1 dose, 54 studies after 2 doses, 11 studies after 3 doses, and 2 studies after 4 doses of the mRNA COVID-19 vaccines.

**Figure 2.**
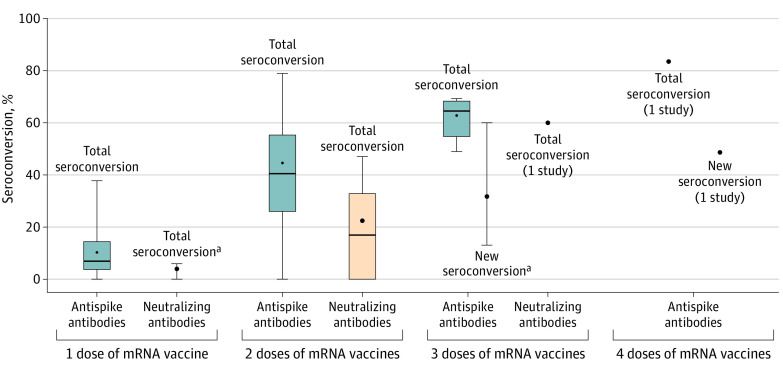
Antibody Response of mRNA Vaccines Total seroconversion includes all seroconversion regardless of humeral immune response from the previous dose. New seroconversion only includes seroconversion from patients with no or minimal immune response from the previous dose. Dark lines indicates medians; dots, means; boxes, IQRs; whiskers, ranges. ^a^Box plot cannot be graphed because fewer than 5 studies were included.

Among the studies analyzed, the weighted mean (range) seroconversion rate after 1 dose of mRNA vaccine was 10.4% (0%-37.9%) for antispike antibodies (18 studies^12,33,35,37,40,41,42,44,48,51,52,55,57,69,71,76,103,105^) and 4.1% (0%-5.9%) for neutralizing antibodies (2 studies^12,69^) (Figure 2). The mean (range) antibody testing time was 25.5 (21-28) days after the first dose.

The weighted mean (range) total seroconversion rate after 2 doses of mRNA COVID-19 vaccines was 44.9% (0%-79.1%) for antispike antibodies (53 studies^10,11,12,13,14,32,35,36,38,40,41,42,43,45,46,47,48,49,50,51,52,53,54,55,56,57,60,61,62,63,64,65,66,67,69,70,71,74,79,80,82,84,86,88,91,92,93,94,95,97,102,103,105^) and 22.6% (0%-47.5%) for neutralizing antibodies (8 studies^12,49,62,65,67,69,71,95^) (Figure 2). Among studies reporting seroconversion rates after 2 doses of mRNA COVID-19 vaccines, we reviewed the rates of positive antibody response by types of the mRNA COVID-19 vaccines. The BNT162b2 vaccine had a weighted mean (range) seroconversion rate of 44% (range 0%-79.1%) for antispike antibodies (36 studies^11,13,35,40,41,42,43,45,46,47,49,50,51,52,53,54,56,57,60,62,63,64,65,66,67,69,70,80,84,86,88,91,92,93,102,105^) and 15.3% (0%-35%) for neutralizing antibodies (5 studies^49,62,65,67,69^). The mRNA-1273 vaccine demonstrated a weighted mean (range) seroconversion rate of 51.4% (29.9%-76.2%) for antispike antibodies (7 studies^10,12,32,48,79,103,105^) and a mean of 26.9% for neutralizing antibodies (1 study^12^). The mean (range) antibody testing time after the second dose was 31.9 (8-81) days for all vaccines, 33.8 (8-81) days for the BNT162b2 vaccine, and 25.2 (14-28) days for the mRNA-1273 vaccine. Humoral immune response rates after 2 doses of mRNA vaccines by the different testing modalities are summarized in eTable 4 in the Supplement.

Three doses of mRNA vaccines showed higher total seroconversion rates (including all seroconversion regardless of humoral immune response from the second dose) with a weighted mean (range) of 63.1% (49.1%-69.1%) for antispike antibodies (8 studies^31,41,52,56,72,78,81,85,99^) and a mean of 60% for neutralizing antibodies (1 study^81^) (Figure 2). Two studies reported new seroconversion (ie, only study participants with no or minimal immune response after the second dose), with the weighted mean (range) seropositivity rate of 32% (13.3%-60%) for antispike antibodies (Figure 2). The mean (range) antibody testing time was 26.3 (14-30) days after the third dose. A study by Schrezenmeier et al^104^ reported a 36% antispike antibodies response rate and a 35% neutralizing antibodies response rate after 2 doses of BNT162b2 followed by either 1 dose of BNT162b2 or AZD1222 (University of Oxford and Vaccitech).

A study by Alejo et al^30^ reported high positive antibody response rates after 4 doses of mRNA vaccines, with a mean response rate of 83.3% for antispike antibodies; however, the study also included study participants with positive antibody response after the third dose. A study by Kamar et al^98^ included only study participants with negative or low positive antibody response after the third dose, and reported a seropositivity rate of 48.7% after 4 doses of the BNT162b2 vaccine for antispike antibodies.

#### Other Vaccine Platforms

For study participants vaccinated with the viral-vectored vaccine platform, Boyarsky et al^9^ reported that 16.7% had positive antispike antibodies after 1 dose of Ad26.COV2.S, and Prendecki et al^86^ reported 43.6% had positive antispike antibodies after 2 doses of AZD1222. Masset et al^100^ reported that either 2 doses of AZD1222 followed by 1 dose of mRNA vaccine or 1 dose of AZD1222 followed by 2 doses of mRNA vaccine resulted in antispike antibody seroconversion in 75% of participants. Among inactivated COVID-19 vaccine platforms, only CoronaVac (Sinovac Biotech) has been studied in recipients of kidney transplants, and the seroconversion rate for antispike antibodies was 15.2% after 1 does (1 study^58^) and 40.8% (range, 18.8%-43%) after 2 doses.^58,89^ There were no studies available for other vaccine platforms response in recipients of SOT at the time of our data search. Humoral immune response rates by the COVID-19 vaccine types and doses are summarized in eTable 5 in the Supplement.

### Factors Associated With Reduced Humoral Immune Responses After 2 Doses of mRNA Vaccines

#### Host Characteristics

**Figure 3.**
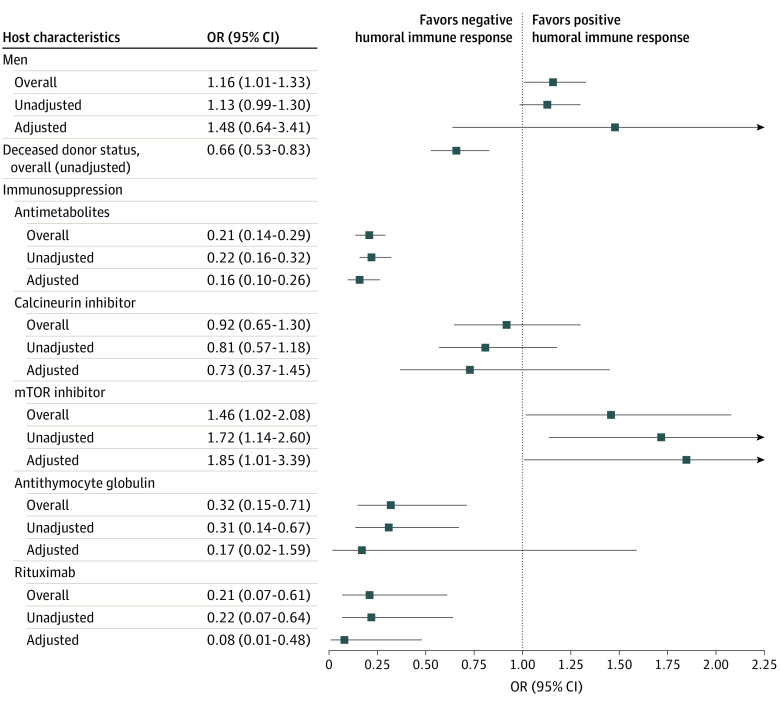
Overall, Unadjusted, and Adjusted Pooled Odds Ratios (ORs) Accounting for Confounders mTOR indicates mammalian (mechanistic) target of rapamycin.

Increased age was associated with lower seroconversion rates. The pooled difference in means (SE) of 10 studies showed study participants with antibody response were 3.94 (1.1) years younger than those without antibody response (*P* = .001)^45,46,47,49,53,56,59,63,66,103^ (Table; eFigure 1 in the Supplement). Male sex was associated with higher seroconversion rates (pOR, 1.16 [95% CI, 1.01-1.33]; *P* = .04; *I*^2^ = 0%) (26 studies^32,45,46,47,49,50,53,56,57,59,62,63,64,66,70,74,79,80,82,84,86,88,92,93,94,103^) (Table, Figure 3; eFigure 1 in the Supplement). Body mass index (BMI) and absolute lymphocyte count were not associated with differences in antibody response based on differences of means that were not statistically significant (Table; eFigure 1 in the Supplement).

**Table.** Summary of Factors Associated With Immunogenicity After 2 Doses of mRNA Vaccines

Risk factor	Positive humoral immune response, pOR (95% CI)	Pooled difference in positive humoral immune response, mean (SE)	Studies, No.	Certainty of evidence (GRADE)	Comments
Host characteristics					
Age	NA	–3.94 (1.1)	10	Very low	None
Male	1.16 (1.01-1.33)	NA	26	Low	pOR of male sex lost significance after removing 1 of several studies from analysis
BMI	NA	0.15 (0.23)	9	Very low	None
Lymphocyte count	NA	0.16 (0.13)	4	Very low	None
Transplant characteristics					
Time from transplant, y	NA	2.12 (0.71)	9	Low	None
Deceased donor status	0.66 (0.53-0.83)	NA	10	Moderate	None
Maintenance IS					
Antimetabolites	0.21 (0.14-0.29)	NA	25	Low	pOR of mTOR inhibitors lost significance after removing 1 of several studies from analysis; it also lost significance after accounting for publication bias.
Calcineurin inhibitors	0.92 (0.65-1.30)	NA	17	Low	None
mTOR inhibitors	1.46 (1.02-2.08)	NA	21	Very low	None
Augmented IS in 12 mo					
Antithymocyte globulin	0.32 (0.15-0.71)	NA	5	Very low	pORs of rituximab exposure lost significance after removing Haskin et al^47^ study from the analysis
Rituximab	0.21 (0.07-0.61)	NA	5	Moderate	None

Abbreviations: BMI, body mass index (calculated as weight in kilograms divided by height in meters squared); IS, immunosuppression; mTOR, mammalian (mechanistic) target of rapamycin; NA, not applicable; pORs, pooled odds ratios.

#### Transplant Characteristics

Receipt of a deceased donor organ was associated with lower seroconversion rates compared with living donor status (pOR, 0.66 [95% CI, 0.53-0.83]; *P* < .001; *I*^2^ = 0%) (10 studies^32,45,47,49,56,59,66,84,94,103^) (Table, Figure 3; eFigure 1 in the Supplement). Time from transplantation to vaccination was associated with seroconversion rate. The pooled difference in means (SE) from 9 studies^45,46,47,49,53,56,59,63,66^ showed study participants with positive antibody response had 2.17 (0.71) years longer from transplantation to vaccination compared with those without antibody response (*P* = .002) (Table; eFigure 1 in the Supplement).

#### Immunosuppression

A total of 25 studies^32,45,46,49,50,53,56,57,59,62,63,64,66,70,74,79,80,82,88,91,92,93,94,95,103^ reported on antimetabolite use at the time of vaccine administration, which was associated with lower seroconversion rates (pOR, 0.21 [95% CI, 0.14-0.29]; *P* < .001; *I*^2^ = 70%) (Table, Figure 3; eFigure 1 in the Supplement). A total of 21 studies^10,32,45,46,49,50,53,56,57,62,63,64,66,70,74,80,82,84,88,93,103^ reported active use of mammalian (mechanistic) target of rapamycin (mTOR) inhibitors, which was associated with higher seroconversion rates (pOR, 1.46 [95% CI, 1.02-2.08]; *P* = .04; *I*^2^ = 42%) (Table, Figure 3; eFigure 1 in the Supplement). Furthermore, 17 studies^32,45,46,49,50,53,56,57,63,64,74,79,80,82,84,92,103^ reported calcineurin inhibitor (CNI) use at the time of vaccine administration, which was not associated with antibody response (pOR, 0.92 [95% CI, 0.65-1.30]; *P* = .64; *I*^2^ = 21%) (Table, Figure 3; eFigure 1 in the Supplement).

Both rituximab exposure^45,47,66,74,103^ and antithymocyte globulin (ATG) exposure^10,45,47,66,74^ within 12 months of vaccination were associated with lower seroconversion rates (rituximab: pOR, 0.21 [95% CI, 0.07-0.61]; *P* = .005; *I*^2^ = 0%; ATG: pOR, 0.32 [95% CI, 0.15-0.71]; *P* = .005; *I*^2^ = 0%) (Table, Figure 3; eFigure 1 in the Supplement). All data extraction of potential risk factors are summarized in eTable 6 and eTable 7 in the Supplement.

### Sensitivity Analysis and Publication Bias

Results of sensitivity analysis are reported in eFigure 2 in the Supplement. The pORs of male sex and seroconversion lost significance after removing 1 of the following studies: Cholankeril et al,^92^ Davidov et al,^93^ Ducloux et al,^80^ Haskin et al,^47^ Kantauskaite et al,^82^ Masset et al,^56^ Rozen-Zvi et al,^66^ Sanders et al,^103^ or Villanego et al.^74^ The pORs of rituximab exposure and seroconversion lost significance after removing Haskin et al^47^ from the analysis. The pORs of mTOR inhibitor use and seroconversion lost significance after removing any 1 of the following studies: Benotmane et al,^32^ Cucchiari et al,^10^ Grupper et al,^45^ Kantauskaite et al,^82^ Korth et al,^53^ Peled et al,^62^ Rabinowich et al,^63^ Rashidi-Alavijeh et al,^64^ Sanders et al,^103^ or Villanego et al.^74^

**Figure 4.**
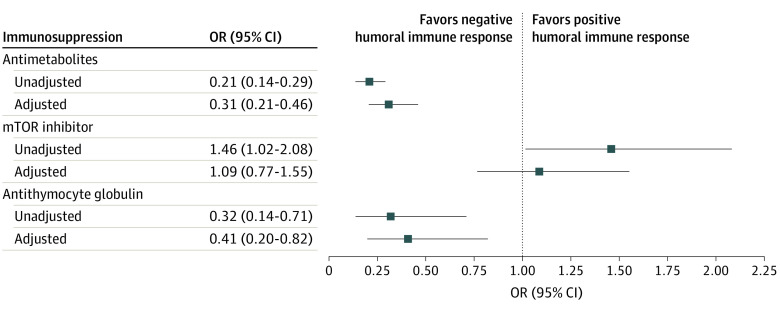
Unadjusted and Adjusted Pooled Odds Ratios (ORs) Accounting for Publication Bias mTOR indicates mammalian (mechanistic) target of rapamycin; OR, odds ratio.

We found evidence of publication bias for age, BMI, lymphocyte count, antimetabolite use, mTOR inhibitor use, and ATG exposure (eFigure 3 in the Supplement). After accounting for publication bias by the Duval and Tweedie trim-and-fill method, antimetabolites (adjusted pOR, 0.31 [95% CI, 0.21-0.46]) and ATG (adjusted pOR, 0.41 [95% CI, 0.20, 0.82]) remained significantly associated with lower seroconversion rates (Figure 4; and eFigure 4 in the Supplement). After adjusting, the association of mTOR inhibitors was no longer significant (adjusted pOR, 1.09 [95% CI, 0.77-1.55]) (Figure 4; and eFigure 4 in the Supplement). The adjusted pooled difference in means of age remained significant, while those of BMI and lymphocyte count remained not statistically significant (eFigure 4 in the Supplement).

## Discussion

This systematic review and meta-analysis summarizes the cumulative evidence of immunogenicity of COVID-19 vaccines and risk factors associated with poor humoral response in recipients of SOT. Despite receiving multiple doses of mRNA vaccines, approximately 20% to 40% of recipients of SOT did not mount an antibody response. Although the correlation between positive humoral immune response with the clinical efficacy of COVID-19 vaccines in recipients of SOT has yet to be determined, the rates of humoral immune response in these populations after 3 or 4 doses of the mRNA COVID-19 vaccines are lower than those found in the general populations of the phase III clinical trials,^7,8,108^ and the rates of COVID-19 vaccine breakthrough infection in recipients of SOT are higher than those of the general public.^39,109^ COVID-19 breakthrough infection rates before the emergence of the Omicron variant in recipients of SOT who had received at least 2 doses of an mRNA vaccine or 1 dose of an adenovirus vaccine varied from 0.23% to 2.5%, as reported among transplant centers in the US. The breakthrough infection rate was up to 5% among recipients of kidney transplant treated with belatacept in a French cohort study,^39^ which is much higher than the general public.^110,111^

Our study has identified several risk factors associated with the lower seroconversion rate after 2 doses of mRNA COVID-19 vaccines in recipients of SOT. Recipients of SOT who were older, had recent transplants, or received deceased donor organ transplants had lower seroconversion rates. Unfortunately, we cannot determine specific cutoffs of age or time after transplantation associated with poor antibody response based on our study design. Similarly, recipients of SOT who were actively using antimetabolite immunosuppression (eg, mycophenolate mofetil, mycophenolic acid, or azathioprine) or who had recent exposure to rituximab or ATG within 12 months had lower seroconversion rates. We hypothesize that lower seroconversion with these agents could be caused by direct suppression of B-lymphocyte function or suppression of T-lymphocyte-dependent B-lymphocyte activation.^112,113,114^ Currently, there are ongoing clinical trials in Israel (NCT04961229), the US (NCT04969263), and the Netherlands (NCT05030974) to evaluate immunogenicity in solid organ transplant recipients after modulation of immunosuppression.

Despite higher seroconversion rates with progressively higher numbers of vaccine doses in recipients of SOT, the durability of the antibody response to repeated vaccination and the clinical outcomes in infection rates, disease severity, and mortality remain unknown.^81^ As of December 20, 2021, the US FDA has issued an EUA for tixagevimab-cilgavimab, a combination of long-acting monoclonal antibodies (mAb), for preexposure prophylaxis in patients who are immunocompromised, including recipients of SOT.^115,116^ Although the EUA states that mAb preexposure prophylaxis should not be considered an alternative to vaccination, there is inherent tension between the strategy of active immunization through vaccination and passive immunization with mAbs. Long-acting mAb preexposure prophylaxis is a valuable resource to add protection for recipients of SOT who have received all available and recommended doses of COVID-19 vaccines but who have demonstrated poor humoral immunity or who have the risks factors associated with lower seroconversion rates identified in our study. But for recipients of SOT who have not received all available doses of COVID-19 vaccines, there are now 2 options: to proceed with vaccination according to the recommended schedule or to postpone vaccination and pursue mAbs. The risk factors identified in this study for lower seroconversion rates after 2 vaccine doses may also help to inform transplant professionals who must advise their patients on time-sensitive decisions about active vs passive immunization by weighing the likelihood of benefit from vaccination compared with the likelihood of benefit from mAbs.

Additional unanswered questions include the optimal number of doses for a primary vaccination series, particularly in recipients of SOT with risk factors associated with lower seroconversion rates. Additionally, the role of antibody testing to determine strategies for additional doses or mAbs, the appropriate antibody cutoff level or other tests to ascertain immunity, and the most just allocation of scarce vaccine supply toward first doses or additional and booster doses at the global level are questions worthy of investigation and discussion.

### Limitation

This study has some limitations. One limitation of COVID-19 vaccine immunogenicity research in recipients of SOT to date is the overrepresentation of the mRNA platform. Many recipients of SOT, particularly those living outside the US, may not have been fully vaccinated against COVID-19 or may not have access to mRNA vaccines owing to vaccine scarcity. Second, several techniques of SARS-CoV-2 antibody testing were used in the studies, and there is no criterion standard at this time. Third, the correlation between humoral immune response and the clinical efficacy of COVID-19 vaccines in recipients of SOT remains unclear. Fourth, most study participants were recipients of kidney transplant, with relatively fewer other organ transplant types. Fifth, the data regarding other vaccine platforms, including the heterologous prime-boost strategy, in recipients of SOT are extremely limited.

## Conclusions

In this systematic review and meta-analysis of 29 studies and 11 713 recipients of SOT, seroconversion rates among recipients of SOT vaccinated with mRNA vaccines were higher with successive doses but remained lower than those among the general population. The availability of long-acting mAbs for preexposure prophylaxis presented an additional option for solid organ transplant recipients who have been vaccinated but may still be at inordinate risk for COVID-19, and mAbs are an additional consideration for recipients of SOT who have not yet been vaccinated and who may have multiple risk factors associated with a lower immune response to vaccination.

## Notes

Supplement. **eMethods.** Search Strategies **eTable 1.** Newcastle-Ottawa Quality Assessment Scale of Included Studies in Meta-Analysis **eTable 2.** Study Characteristics **eTable 3.** Grading of Recommendation Assessment, Development and Evaluation (GRADE) for Potential Factors Associated With Diminished Humoral Immune Response **eTable 4.** Humoral Immune Response Rates After 2 Doses of mRNA Vaccines by the Different Testing **eTable 5.** Humoral Immune Response by the COVID-19 Vaccine Types and Doses **eTable 6.** Participants and the Effect Estimates of Potential Risk Factors for Diminished Humoral Immune Response in Each Study **eTable 7.** Continuous Data of Potential Risk Factors for Diminished Humoral Immune Response **eFigure 1.** Forest Plots of Studied Risk Factors **eFigure 2.** Sensitivity Analysis of Studied Risk Factors **eFigure 3.** Publication Bias of Studied Risk Factors **eFigure 4.** Funnel Plots and Adjusted Effect Estimates Accounting for Publication Bias
eReferences.
