# Supplementary material for: Immunogenicity and Risk Factors Associated With Poor Humoral Immune Response of SARS-CoV-2 Vaccines in Recipients of Solid Organ Transplant: A Systematic Review and Meta-Analysis
Source: JAMA Netw Open. 2022 Apr 12;5(4):e226822. doi: 10.1001/jamanetworkopen.2022.6822 (PMC9006106; doi:10.1001/jamanetworkopen.2022.6822)
Supplement: Supplement. — eMethods. Search Strategies eTable 1. Newcastle-Ottawa Quality Assessment Scale of Included Studies in Meta-Analysis eTable 2. Study Characteristics eTable 3. Grading of Recommendation Assessment, Development and Evaluation (GRADE) for Potential Factors Associated With Diminished Humoral Immune Response eTable 4. Humoral Immune Response Rates After 2 Doses of mRNA Vaccines by the Different Testing eTable 5. Humoral Immune Response by the COVID-19 Vaccine Types and Doses eTable 6. Participants and the Effect Estimates of Potential Risk Factors for Diminished Humoral Immune Response in Each Study eTable 7. Continuous Data of Potential Risk Factors for Diminished Humoral Immune Response eFigure 1. Forest Plots of Studied Risk Factors eFigure 2. Sensitivity Analysis of Studied Risk Factors eFigure 3. Publication Bias of Studied Risk Factors eFigure 4. Funnel Plots and Adjusted Effect Estimates Accounting for Publication Bias eReferences. [file jamanetwopen-e226822-s001.pdf]

Supplemental Online Content

Manothummetha K, Chuleerarux N, Sanguankeo A, et al. Immunogenicity and risk factors associated with poor humoral immune response of SARS-CoV-2 vaccines in recipients of solid organ transplant: a systematic review and meta-analysis. *JAMA Netw Open*. 2022;5(4):e226822. doi:10.1001/jamanetworkopen.2022.6822

eMethods. Search Strategies

eTable 1. Newcastle-Ottawa Quality Assessment Scale of Included Studies in Meta-Analysis

eTable 2. Study Characteristics

eTable 3. Grading of Recommendation Assessment, Development and Evaluation (GRADE) for Potential Factors Associated With Diminished Humoral Immune Response

eTable 4. Humoral Immune Response Rates After 2 Doses of mRNA Vaccines by the Different Testing

eTable 5. Humoral Immune Response by the COVID-19 Vaccine Types and Doses

eTable 6. Participants and the Effect Estimates of Potential Risk Factors for Diminished Humoral Immune Response in Each Study

eTable 7. Continuous Data of Potential Risk Factors for Diminished Humoral Immune Response

eFigure 1. Forest Plots of Studied Risk Factors

eFigure 2. Sensitivity Analysis of Studied Risk Factors

eFigure 3. Publication Bias of Studied Risk Factors

eFigure 4. Funnel Plots and Adjusted Effect Estimates Accounting for Publication Bias

eReferences.

This supplemental material has been provided by the authors to give readers additional information about their work.

© 2022 Manothummetha K et al. *JAMA Network Open.*

e**Methods.** Search Strategies

OVID Medline

1. exp COVID-19/ or (COVID-19 or SARS-CoV-2 or 2019 Novel Coronavirus or 2019-nCoV).mp.
2. (Ad26*COV*S or Janssen or JNJ-78436735 or Ad26COVS1 or VAC31518).mp.
3. (AZD1222 or ChAdOx1 nCoV-19 or AZ or astrazeneca or ChAdOx1-S or AZD2816).mp.
4. (BNT162b2 or Pfizer or biontech or Comirnaty or Tozinameran).mp.
5. (Coronavac or Sinovac or PiCoVacc).mp.
6. (mRNA-1273 or Moderna).mp.
7. ((WIV04 and HB02) or Sinopharm).mp.
8. exp Vaccination/ or exp Vaccines/ or vaccin*.mp.
9. exp Immunization/ or immuni*ation.mp.
10. 8 or 9
11. 1 and 10
12. exp COVID-19 Vaccines/ or ((COVID-19 or SARS-CoV-2 or 2019 Novel Coronavirus or 2019-nCoV) adj2 Vaccin*).tw.
13. 2 or 3 or 4 or 5 or 6 or 7 or 11 or 12
14. exp Organ Transplantation/ or organ transplant*.mp.
15. heart*.mp. or exp Heart/
16. (cardiac or cardio*).mp.
17. lung*.mp. or exp Lung/
18. pulmonary.mp.
19. exp Kidney/ or kidney*.mp.
20. renal.mp.
21. liver.mp. or exp Liver/
22. hepatic.mp.
23. pancrea*.mp. or exp Pancreas/
24. intestin*.mp. or exp Intestines/ or exp Intestine, Small/
25. 15 or 16 or 17 or 18 or 19 or 20 or 21 or 22 or 23 or 24
26. transplant*.mp. or exp Transplant Recipients/
27. 25 and 26
28. exp Heart Transplantation/ or heart transplant*.mp.
29. exp Lung Transplantation/ or lung transplant*.mp.
30. exp Kidney Transplantation/ or kidney transplant*.mp.
31. exp Liver Transplantation/ or liver transplant*.mp.
32. exp Pancreas Transplantation/ or pancrea* transplant*.mp.
33. Intestin* transplant*.mp.
34. transplant*.mp.
35. 14 or 27 or 28 or 29 or 30 or 31 or 32 or 33 or 34
36. 13 and 35

© 2022 Manothummetha K et al. *JAMA Network Open.*

Embase:

1. 'sars-cov-2 vaccine'/exp OR 'sars-cov-2 vaccine'
2. 'ad26.cov2.s vaccine'/exp OR 'ad26.cov2.s vaccine'
3. 'tozinameran'/exp OR 'tozinameran'
4. 'chadox1 ncov 19'/exp OR 'chadox1 ncov 19'
5. 'picovacc vaccine'/exp OR 'picovacc vaccine'
6. 'mrna-1273 vaccine'/exp OR 'mrna-1273 vaccine'
7. 'bbibp corv vaccine'/exp OR 'bbibp corv vaccine'
8. 'coronavirus disease 2019'/exp OR 'coronavirus disease 2019'
9. 'vaccin*' OR 'vaccine'/exp OR 'vaccine' OR 'vaccination'/exp OR 'vaccination'
10. 'immunization'/exp OR 'immunization'
11. #9 OR #10
12. #8 AND #11
13. #1 OR #2 OR #3 OR #4 OR #5 OR #6 OR #7 OR #12
14. 'organ transplant*' OR 'organ transplantation'/exp OR 'organ transplantation'
15. 'heart'/exp OR 'heart' OR 'cardio*' OR 'cardiac'
16. 'lung'/exp OR 'lung' OR 'pulmonary'
17. 'kidney'/exp OR 'kidney' OR 'renal'/exp OR 'renal'
18. 'liver'/exp OR 'liver' OR 'hepatic'
19. 'pancreas'/exp OR 'pancreas' OR 'pancrea*'
20. 'intestine'/exp OR 'intestine' OR 'intestin*'
21. 'transplant'/exp OR 'transplant' OR 'transplantation'/exp OR 'transplantation' OR 'transplant*' OR 'graft recipient'/exp OR 'graft recipient' OR 'transplant* recipient*'
22. #15 OR #16 OR #17 OR #18 OR #19 OR #20
23. #21 AND #22
24. ('organ'/exp OR organ OR 'heart'/exp OR heart OR 'lung'/exp OR lung OR 'renal'/exp OR renal OR'liver'/exp OR liver OR intestinal) AND transplant*
25. #14 OR #23 OR #24
26. 'heart graft'/exp OR 'heart graft' OR 'heart transplantation'/exp OR 'heart transplantation' OR 'heart transplant*'
27. 'lung transplantation'/exp OR 'lung transplantation' OR 'lung transplant*'
28. 'kidney graft'/exp OR 'kidney graft' OR 'kidney transplantation'/exp OR 'kidney transplantation'OR 'kidney transplant*'
29. 'liver graft'/exp OR 'liver graft' OR 'liver transplantation'/exp OR 'liver transplantation' OR 'liver transplant*'
30. 'pancreas transplantation'/exp OR 'pancreas transplantation' OR 'pancrea* transplant*'
31. 'intestine graft'/exp OR 'intestine graft' OR 'intestine transplantation'/exp OR 'intestine transplantation' OR 'intestin* transplant*'
32. #25 OR #26 OR #27 OR #28 OR #29 OR #30 OR #31 OR ‘transplant/exp OR ‘transplant*’
33. #13 AND #32

© 2022 Manothummetha K et al. *JAMA Network Open.*

ISI Web of Science

1. ALL=(COVID-19 OR SARS-CoV-2 OR 2019 Novel Coronavirus OR 2019*nCoV)
2. ALL=(vaccin* or immuni*ation)
3. 1 AND 2
4. ALL=(Ad26*COV*S OR JNJ*78436735 OR Ad26COVS1 OR VAC31518)
5. ALL=(AZD1222 or ChAdOx1 nCoV*19 or ChAdOx1*S or AZD2816)
6. ALL=(BNT162b2 or biontech or Comirnaty or Tozinameran)
7. ALL=(Coronavac or Sinovac or PiCoVacc)
8. ALL=(mRNA-1273 or Moderna)
9. ALL=(WIV04 and HB02) or Sinopharm)
10. 3 OR 4 OR 5 OR 6 OR 7 OR 8 OR 9
11. 10 AND ALL=(transplant*)

Cochrane Library

1. MeSH descriptor: [COVID-19] explode all trees
2. COVID-19 OR SARS-CoV-2 OR 2019 Novel Coronavirus OR 2019*nCoV
3. MeSH descriptor: [Vaccines] explode all trees
4. Vaccin* OR immuni*ation
5. (#1 OR #2) AND (#3 OR #4)
6. MeSH descriptor: [COVID-19 Vaccines] explode all trees
7. Ad26*COV*S OR Janssen OR JNJ*78436735 OR Ad26COVS1 OR VAC31518
8. AZD1222 or ChAdOx1 nCoV*19 or AZ or astrazeneca or ChAdOx1*S or AZD2816
9. BNT162b2 or Pfizer or biontech or Comirnaty or Tozinameran
10. Coronavac or Sinovac or PiCoVacc
11. mRNA-1273 or Moderna
12. (WIV04 and HB02) or Sinopharm
13. #5 or #6 or #7 or #8 or #9 or #10 or #11 or #12
14. MeSH descriptor: [Heart] explode all trees
15. cardiac or cardio* or heart
16. MeSH descriptor: [Lung] explode all trees
17. lung* or pulmonary
18. MeSH descriptor: [Kidney] explode all trees
19. kidney* or renal
20. MeSH descriptor: [Liver] explode all trees
21. liver or hepatic
22. MeSH descriptor: [Pancreas] explode all trees
23. pancrea*
24. MeSH descriptor: [Intestines] explode all trees
25. intestin*
26. #14 or #15 or #16 or #17 or #18 or #19 or #20 or #21 or #22 or #23 or #24 or #25
27. MeSH descriptor: [Transplantation] explode all trees
28. MeSH descriptor: [Transplant Recipients] explode all trees
29. transplant*
30. #27 or #28 or #29
31. #26 and #30

© 2022 Manothummetha K et al. *JAMA Network Open.*

32. MeSH descriptor: [Heart Transplantation] explode all trees
33. MeSH descriptor: [Lung Transplantation] explode all trees
34. MeSH descriptor: [Kidney Transplantation] explode all trees
35. MeSH descriptor: [Liver Transplantation] explode all trees
36. MeSH descriptor: [Pancreas Transplantation] explode all trees
37. intestin* transplant*
38. #32 or #33 or #34 or #35 or #36 or #37
39. #31 or #38
40. #13 AND #39

Clinicaltrial.gov

(heart OR cardiac OR cardio OR lung OR pulmonary OR liver OR hepatic OR renal OR kidney OR intestin OR pancrea) AND transplant | COVID-19 vaccin OR BNT162b2 OR Pfizer OR Tozinameran OR Ad26.COV.S OR Janssen OR JNJ-78436735 OR Ad26COVS1 OR VAC31518 OR AZD1222 OR ChAdOx1 nCoV-19 OR AZ OR astrazeneca OR ChAdOx1-S OR AZD2816 OR mRNA-1273 OR CoronaVac OR Sinopharm

© 2022 Manothummetha K et al. *JAMA Network Open.*

eTable 1. Newcastle-Ottawa Quality Assessment Scale of Included Studies in Meta-Analysis

Study/Year	Selection				Comparability	Outcome			Total score
	**Representativeness**	**Selection of the non-exposed cohort**	**Ascertainment**	**Endpoint does not present at start**	**Comparability (Confounding)**	**Assessment of outcome**	**Follow-up duration**	**Adequacy follow-up**	
Alejo 2021[1]	*	*	*	*	**	*	*	*	9
Azzi 2021 [2]	*	*		*	**	*	*	*	8
Ben Zadok 2021[3]	*	*	*	*	**	*	*	*	9
Benotmane 2021 (1 dose Moderna) [4]	*	*		*		*	*	*	6
Benotmane 2021 (2 doses Moderna) [5]	*	*		*		*	*	*	6
Benotmane 2021 (3 doses)[6]	*	*		*		*	*	*	6
Bergman 2021 [7]	*	*		*	*	*	*	*	7
Bertrand 2021 [8]	*	*	*	*	**	*	*	*	9
Bertrand 2021[9]	*	*	*	*	**	*	*	*	9
Bertrand 2021 (3 doses) [10]	*	*		*	**	*	*	*	8
Boyarsky 2021 (1 dose of mRNA vaccines) [11]		*	*	*	**	*	*	*	8
Boyarsky 2021 (2 doses of mRNA vaccines)[12]		*	*	*	**	*	*	*	8
Boyarsky 2021 (Janssen)[13]	*	*	*	*	**	*	*	*	9
Cao 2021[14]	*	*	*	*	**	*	*	*	9
Chavarot 2021 [15]	*	*	*	*	**	*	*	*	9
Chavarot 2021 (3 doses)[16]	*	*	*	*	**	*	*	*	9

© 2022 Manothummetha K et al. *JAMA Network Open.*

Cholankeril 2021	*		*	*	*	*	*	*	7
Crespo 2021 [17]	*	*	*	*		*	*	*	7
Cucchiari 2021[18]	*	*	*	*		*	*	*	7
D'offizi 2021 [19]	*			*	*	*	*	*	6
Danthu 2021 [20]	*	*	*	*	**	*	*	*	9
Davidov 2021[21]	*	*	*	*	*	*	*	*	8
Debska-slizien 2021 [22]	*	*	*	*	*	*	*	*	8
Del Bello 2021[23]	*	*		*	**	*	*	*	8
Ducloux 2021[24]	*	*	*	*		*	*	*	7
Firket 2021[25]	*	*		*	**	*	*	*	8
Georgery 2021 (1st dose)[26]	*	*	*	*	**	*	*	*	9
Georgery 2021 (2nd dose)[27]	*	*	*	*	**	*	*	*	9
Grupper 2021[28]	*	*	*	*	*	*	*		7
Guarino 2021[29]	*		*	*		*	*		5
Hall 2021[30]	*		*	*		*	*	*	6
Hall 2021 (3 doses)[31]	*	*	*	*	**	*	*	*	9
Haskin 2021[32]	*	*	*	*	**	*	*		8
Havlin 2021[33]	*		*	*	**	*	*	*	8
Havlin 3031 (3 doses) [34]				*	*	*	*	*	5
Herrera 2021[35]	*		*	*		*	*	*	6
Hod 2021[36]	*		*	*	*	*	*		6
Hoffman 2021 [37]	*			*	*	*	*	*	6
Holden 2021[38]	*		*	*		*	*		5
Kamar 2021[39]	*		*	*		*	*		5
Kamar 2021 (4 doses) [40]				*	*	*	*	*	5
Kantauskaite 2021[41]	*		*	*		*	*		5

© 2022 Manothummetha K et al. *JAMA Network Open.*

Korth 2021[42]	*		*	*	*	*	*		6
Kumar 2021 [43]	*	*		*	*	*	*	*	7
Marinaki 2021[44]	*		*	*		*	*		5
Marion 2021[45]	*		*	*		*	*		5
Massa 2021[46]	*				*	*	*	*	5
Masset 2021[47]	*		*	*		*	*		5
Masset 2021 (heterozygous vs homozygous vaccine) [48]									
Mazzola 2021[49]	*		*	*		*	*		5
Medina-Pestana 2021[50]	*		*	*		*	*		5
Middleton 2021[51]	*		*	*		*	*		5
Midtvedth 2021[52]	*		*	*		*	*		5
Miele 2021[53]	*		*	*		*	*		5
Narasimhan 2021[54]	*		*	*		*	*		5
Noble 2021[55]	*	*	*	*	**	*	*		8
Pedersen 2021[56]	*	*		*		*	*	*	6
Peled 2021[57]	*			*		*	*	*	5
Peled 2021 (3 doses)[58]	*		*	*		*	*	*	6
Prendecki 2021[59]	*	*	*	*		*	*	*	7
Quiroga 2021[60]	*		*		*	*	*	*	6
Rabinowich 2021[61]	*	*	*	*	*	*	*	*	8
Rashidi-Alavijeh 2021[62]	*	*	*	*	*	*	*	*	8
Rahav 2021[63]	*	*		*	*	*	*	*	7
Rincon-Arevalo 2021[64]	*	*	*	*		*	*	*	7
Rozen-Zvi 2021[65]	*		*	*		*	*	*	6
Ruether 2021[66]	*	*	*	*	*	*	*	*	8

© 2022 Manothummetha K et al. *JAMA Network Open.*

Russo 2021[67]	*		*	*		*	*	*	6
Sadioğlu 2021[68]	*		*	*		*	*	*	6
Sanders 2021[69]	*	*		*	*	*	*	*	7
Sattler 2021[70]	*	*	*	*	*	*	*	*	8
Schmidt 2021[71]	*	*	*	*	*	*	*	*	8
Schramm 2021[72]	*	*	*	*		*	*	*	7
Schrezenmeier 2021 [73]	*				*	*	*	*	5
Shostak 2021[74]	*		*	*		*	*	*	6
Stumpf 2021 (Sub-analysis of DIAVAC)[75]	*			*		*	*	*	5
Stumpf 2021[76]	*	*	*	*		*	*	*	7
Thuluvath 2021[77]	*	*		*		*	*	*	7
Villanego 2021[78]	*		*	*		*	*	*	6
Werbel 2021[79]	*			*		*	*	*	5
Westhoff 2021[80]	*		*	*		*	*	*	6
Wijtvliet 2021[81]	*			*	*	*	*	*	6
Yi 2021[82]	*	*	*	*		*	*	*	7

© 2022 Manothummetha K et al. *JAMA Network Open.*

eTable 2. Study Characteristics

Study	Vaccine	Country	Type of organ transplantation	Study design	Number of patients for analysis	Dose (time to antibody testing after the last dose unless otherwise stated)	Antibody measurement	Methods	Responders/total (%)
Alejo 2021[1]	BNT162b2 or mRNA-1273	USA	Kidney<br>Liver<br>Heart<br>Pancreas	Prospective	18	4 doses<br>(2-6 weeks)	Anti-Spike Ab	Elecsys anti-SARS-CoV-2 (Roche) or EUROIMMUN SARS-CoV-2 ELISA assay	15/18 (83.3)<br>Pre-dose 4, 6 with negative titers, 2 with low-positive titers, and 10 with high positive titers
Azzi 2021 [2]	BNT162b2 or mRNA-1273 or Ad26.CoV.2	USA	Kidney	Prospective	76	2 doses for BNT162b2 and mRNA-1273, 1 dose for Ad26.CoV2.S<br>(median 28 days)	Anti-spike Ab	VITROS Immunodiagnostic Products Anti-SARS-CoV-2 IgG<br>(Ortho-Clinical Diagnostics)	24/76 (31.6)
Ben Zadok 2021[3]	BNT162b2	Israel	Heart	Prospective	37	1 dose<br>(21-26 days)<br>2 doses<br>(35-40 days)	Anti-spike Ab	The ARCHITECT SARS-CoV-2 IgG II Quant assay (Abbott)	6/39 (15.4)<br><br>18/37 (48.7)
Benotmane 2021<br>(1 dose) [4]	mRNA-1273	France	Kidney	Prospective	241	1 dose<br>(28 days)	Anti-spike Ab	The ARCHITECT SARS-CoV-2 IgG II Quant assay (Abbott)	26/241 (10.8)
Benotmane 202 1<br>(2 doses) [5]	mRNA-1273	France	Kidney	Prospective	204	2 doses (28 days)	Anti-spike Ab	The ARCHITECT SARS-CoV-2 IgG II Quant assay (Abbott)	98/204 (48)
Benotmane 202 1<br>(3 doses)[6]	mRNA-1273	France	Kidney	Prospective	159	3 doses<br>(median 28 days)	Anti-spike Ab	The ARCHITECT SARS-CoV-2 IgG II Quant assay (Abbott)	78/159 (49.1)
Bergman 2021 [7]	BNT162b2	Sweden	Kidney, Kidney-pancreas, Liver	Prospective	83	2 doses<br>(14 days)	Anti-spike Ab	Elecsys anti-SARS-CoV-2 (Roche)	36/83 (43.4)
Bertrand 2021 [8]	BNT162b2	France	Kidney	Retrospective	45	1 dose<br>(3 weeks)<br><br>2 doses<br>(4 weeks)	Anti-spike Ab	The ARCHITECT SARS-CoV-2 IgG II Quant assay (Abbott)	1/45 (2.2)<br><br>8/45 (17.8)

© 2022 Manothummetha K et al. *JAMA Network Open.*

Bertrand 2021[9]	BNT162b2	France	Kidney	Retrospective	235	2 doses<br>(median 34.6 days)	Anti-spike Ab	The ARCHITECT SARS-CoV-2 IgG II Quant assay (Abbott)	65/235 (27.7)
Bertrand 2021 (3 doses) [10]	BNT162b2	France	Kidney	Retrospective	80	2 doses (median 34.5 days)<br><br>3 doses (1 month)	Anti-spike Ab	The ARCHITECT SARS-CoV-2 IgG II Quant assay (Abbott)	30/80 (37.5)<br><br>49/80 (61.2)
Boyarsky 2021 (1 dose of mRNA vaccines) [11]	BNT162b2 or mRNA-1273	USA	Kidney<br>Heart<br>Lung<br>Liver<br>Pancreas<br>Other	Prospective	436	1 dose<br>(Median 21 days)	Anti-spike Ab	Elecsys anti-SARS-CoV-2 (Roche) or EUROIMMUN SARS-CoV-2 ELISA assay	76/436 (17.4)
Boyarsky 2021 (2 doses of mRNA vaccines)[12]	BNT162b2 or mRNA-1273	USA	Kidney<br>Heart<br>Lung<br>Liver<br>Pancreas<br>Other	Prospective	658	2 doses<br>(Median 29 days)	Anti-spike Ab	Elecsys anti-SARS-CoV-2 (Roche) or EUROIMMUN SARS-CoV-2 ELISA assays	357/658 (54.3)
Boyarsky 2021 (Janssen)[13]	Ad26.CoV.2	USA	Kidney<br>Liver<br>Heart<br>Lung	Prospective	12	1 dose<br>(median 33 days)	Anti-spike Ab	Elecsys anti-SARS-CoV-2 (Roche)	2/12 (16.7)
Cao 2021[14]	BNT162b2 or mRNA-1273	USA	Lung<br>Kidney<br>Heart<br>Liver<br>Heart-lung	Retrospective	37	2 doses<br>(not reported	Anti-spike Ab	The Alinity I SARS-CoV-2 IgG II assay (Abbott)	15/37 (40.5)
Chavarot 2021[15]	BNT162b2	France	Kidney (all were treated with belatacept)	Prospective	101	1 dose<br>(28 days)<br><br>2 doses<br>(1 month)	Anti-spike Ab	SARS-CoV-2 IgG II Quant assay (Abbott) or total SARS-CoV-2 antibodies ELISA assay (Beijing Wantai Biological Pharmacy Enterprise)	2/101 (2)<br><br>2/35 (5.7)
Chavarot 2021 (3 doses)[16]	BNT162b2	France	Kidney (all were treated with belatacept)	Prospective	62	3 doses<br>(median 28 days)	Anti-spike Ab	SARS-CoV-2 IgG II Quant assay (Abbott)	Only seronegative after 2 doses 4/62 (6)
Cholankeril 2021	BNT162b2	USA	Liver	Prospective	69	2 doses<br>(median (IQR) 81 (72-90) days)	Anti-spike Ab	Quest Diagnostics	33/69 (47.8)
Crespo 2021 [17]	mRNA-1273	Spain	Kidney	Prospective	90	2 doses (28 days)	Anti-spike Ab	LIAISON SARS-CoV-2 TrimericS IgG (DiaSorin)	57/90 (63.3)

© 2022 Manothummetha K et al. *JAMA Network Open.*

Cucchiari 2021[18]	mRNA-1273	Spain	Kidney<br>Kidney-pancreas	Prospective	148	2 doses<br>(2 weeks)	Anti-spike Ab	Luminex	35/117 (29.9)
D'offizi 2021 [19]	BNT162b2 or mRNA-1273	Italy	Liver	Prospective	61	2 doses<br>(2 weeks)	Anti-spike Ab<br><br>Neutralizing Ab	The ARCHITECT SARS-CoV-2 IgG II Quant assay (Abbott)<br><br>SARS-CoV2/Human/ITA/ PAVIA10734/2020	47/61 (77)<br><br>29/61 (47.5)
Danthu 2021 [20]	BNT162b2	France	Kidney	Prospective	74	2 doses (36 days after the second dose)	Anti-spike Ab	LIAISON SARS-CoV-2 TrimericS IgG (DiaSorin)	3/74 (4.1)
Davidov 2021[21]	BNT162b2	Israel	Liver	Prospective	76	2 doses<br>(median (IQR) 36 (17-52) days)	Anti-spike Ab	Not reported	55/76 (72.4)
Debska-slizien 2021 [22]	BNT162b2 or mRNA-1273	Poland	Kidney	Prospective	142	2 (14-21 days)	Anti-spike	LIAISON SARS-CoV-2 TrimericS IgG (DiaSorin)	73/142 (51.4)
Del Bello 2021[23]	BNT162b2	France	Kidney<br>Liver<br>Heart<br>Lung<br>Pancreas<br>Combined	Retrospective	396	1 dose<br>(before the second dose)<br><br>2 doses<br>(before the third dose)<br><br>3 doses<br>(4 weeks)	Anti-spike Ab	Mainly total SARS-CoV-2 antibodies ELISA assay (Beijing Wantai Biological Pharmacy Enterprise)	20/396 (5.1)<br><br>164/396 (41.4)<br><br>269/396 (67.9)
Ducloux 2021[24]	BNT162b2	France	Kidney	Prospective	153	2 doses<br>(2.5 months)	Anti-spike Ab	SARS-CoV-2 immunoassay (Abbott)	81/153 (53)
Firket 2021[25]	BNT162b2	Belgium	Kidney	Prospective	10	1 dose<br>(21 days)<br><br>2 doses<br>(15 days)	Anti-spike Ab	LIAISON SARS-CoV-2 S1/S2 IgG chemiluminescent assay (DiaSorin)	0/10 (0)<br><br>1/10 (10)
Georgery 2021 (1st dose)[26]	BNT162b2	Belgium	Kidney	Prospective	78	1 dose<br>(28 days)	Anti-spike Ab	Elecsys anti-SARS-CoV-2 (Roche)	3/78 (3.9)
Georgery 2021 (2nd dose)[27]	BNT162b2	Belgium	Kidney	Prospective	79	2 doses<br>(28 days)	Anti-spike Ab	Elecsys anti-SARS-CoV-2 (Roche)	39/79 (49.4)
Grupper 2021[28]	BNT162b2	Israel	Kidney	Prospective	136	2 doses<br>(10-20 days)	Anti-spike Ab	LIAISON SARS-CoV-2 S1/S2 IgG chemiluminescent assay (DiaSorin)	51/136 (37.5)
Guarino 2021[29]	BNT162b2	Italy	Liver	Prospective	365	2 doses<br>(4 weeks)	Anti-spike Ab	LIAISON SARS-CoV-2 S1/S2 IgG chemiluminescent assay (DiaSorin)	273/365 (74.8)
Hall 2021[30]	mRNA-1273	Canada	Lung<br>Kidney<br>Kidney-pancreas	Prospective	127	1 dose<br>(4 weeks)<br><br>1 dose	Anti-spike Ab<br><br>Neutralizing Ab	Elecsys anti-SARS-CoV-2 (Roche)<br><br>SARS-CoV-2 Surrogate Virus	6/121 (5)<br><br>7/119 (6)

© 2022 Manothummetha K et al. *JAMA Network Open.*

			Heart<br>Liver<br>Others			(4 weeks)<br><br>2 doses<br>(4-6 weeks)<br><br>2 doses<br>(4-6 weeks)	 <br>Anti-spike Ab<br><br>Neutralizing Ab	Neutralization Test<br>(GenScript)<br><br>Elecsys anti-SARS-CoV-2 (Roche)<br><br>SARS-CoV-2 Surrogate Virus<br>Neutralization Test<br>(GenScript)	 <br>38/110 (34.6)<br><br>29/108 (26.9)
Hall 2021 (3 doses)[31]	mRNA-1273	Canada	Kidney<br>Liver Heart<br>Lung<br>Pancreas<br>Combined organs	Prospective	60	3 doses<br>(4 weeks)	Anti-spike Ab<br><br>Neutralizing antibodies	Elecsys anti-SARS-CoV-2 (Roche)<br><br>SARS-CoV-2 Surrogate Virus Neutralization Test (GenScript)	33/60 (55)<br><br>36/60 (60)
Haskin 2021[32]	BNT162b2	Israel	Kidney	Prospective	38	2 doses<br>(median 37 days)	Anti-spike Ab	SARS-CoV-2 IgG II Quant assay (Abbott)	24/38 (63.2)
Havlin 2021[33]	BNT162b2	Czech Republic	Lung	Prospective	48	2 doses<br>(4-6 weeks)	Anti-spike Ab	EUROIMMUN SARS-CoV-2 ELISA assays	0/21 (0)
Havlin 3031 (3 doses) [34]	BNT162b2	Czech Republic	Lung	Prospective	15	3 doses<br><br>(3 weeks)	Anti-spike Ab	TestLine Clinical Diagnostics	2/15 (13.3)
Herrera 2021[35]	mRNA-1273	Spain	Heart	Prospective	46	1 dose<br>(4 weeks)<br><br>2 doses<br>(4 weeks)	Anti-spike Ab	Siemens COV2T and Siemens COV2G	5/46 (10.9)<br><br>26/46 (56.5)
	mRNA-1273	Spain	Liver	Prospective	58	1 dose<br>(4 weeks)<br><br>2 doses<br>(4 weeks)	Anti-spike Ab	Siemens COV2T and Siemens COV2G	22/58 (37.9)<br><br>41/58 (70.6)
Hod 2021[36]	BNT162b2	Israel	Kidney	Retrospective	120	2 doses<br>(2-4 weeks)	Anti-spike Ab<br><br>Neutralizing Ab	In house ELISA<br><br>SARS-CoV-2 pseudovirus (psSAR-2) neutralization assay (Gert Zimmer)	52/120 (43.3)<br><br>42/120 (35) [responders in this study had both positive anti-RBD and neutralizing Ab]
Hoffman 2021 [37]	BNT162b2 or mRNA-1273	Netherlands	Lung	Prospective	91	2 doses<br>(6 weeks)	Anti-spike Ab	LIAISON SARS-CoV-2 S1/S2 IgG chemiluminescent assay (DiaSorin)	22/80 (27.5)

© 2022 Manothummetha K et al. *JAMA Network Open.*

Holden 2021[38]	BNT162b2	Denmark	Kidney<br>Liver<br>Heart<br>Lung	Prospective	80	2 doses<br>(median 5.6 weeks)	Anti-spike Ab	EUROIMMUN SARS-CoV-2 ELISA assays	28/80 (35)
Kamar 2021[39]	BNT162b2	France	Kidney<br>Liver<br>Heart<br>Lung<br>Pancreas	Prospective	101	1 dose<br>(before second dose)<br><br>2 doses<br>(before third dose)<br><br>3 doses<br>(4 weeks)	Anti-spike Ab	SARS-CoV-2 antibodies ELISA assay (Beijing Wantai Biological Pharmacy Enterprise)	4/101 (4)<br><br>40/99 (40.4)<br><br>67/99 (67.7)
Kamar 2021 (4 doses) [40]	BNT162b2	France	Kidney<br>Heart<br>Liver<br>Pancreas	Prospective	37	4 doses<br>(1 months)	Anti-spike Ab	SARS-CoV-2 antibodies ELISA assay (Beijing Wantai Biological Pharmacy Enterprise)	18/37 (48.6)
Kantauskaite 2021[41]	BNT162b2 or mRNA-1273	Germany	Kidney	Prospective	225	2 doses<br>(mean 14 days)	Anti-spike Ab<br><br>Neutralizing antibodies	Anti-SARS-CoV-2- QuantiVacELISA (Euroimmun)<br><br>SARS-CoV-2 neutralization efficacy (University Hospital Düsseldorf, Germany)	56/225 (24.9)<br><br>38/56 of people with positive anti-spike Ab
Korth 2021[42]	BNT162b2	Germany	Kidney	Prospective	23	2 doses<br>(14 days)	Anti-spike Ab	LIAISON SARS-CoV-2 TrimericS IgG (DiaSorin)	5/23 (21.7)
Kumar 2021 (3 doses) [43]	mRNA-1273	Canada	Lung<br>Heart<br>Kidney<br>Pancreas<br>Kidney-Pancreas<br>Liver	Prospective	60	3 doses<br>(4-6 weeks)	Neutralizing Ab against wild-type virus<br><br>Neutralizing Ab against the Alpha variant<br><br>Neutralizing Ab against the Beta variant<br><br>Neutralizing Ab against the Delta variant	SARS-CoV-2 Surrogate Virus Neutralization Test (GenScript)	40/117 (34.2)<br><br>17/117 (14.5)<br><br>15/117 (12.8)<br><br>21/117 (17.9)
Marinaki 2021[44]	BNT162b2	Greece	Kidney<br><br>Heart	Prospective	34	2 doses<br>(median 10 days)	Anti-spike Ab	SARS-CoV-2 IgG II Quant assay (Abbott)	20/34 (58.8)

© 2022 Manothummetha K et al. *JAMA Network Open.*

Marion 2021[45]	BNT162b2 or mRNA-1273	France	Kidney<br>Heart<br>Liver<br>Pancreas	Prospective	576	1 dose<br>(before second dose)<br><br>2 doses<br>(4 weeks)	Anti-spike Ab	Mainly total SARS-CoV-2 antibodies ELISA assay (Beijing Wantai Biological Pharmacy Enterprise)	37/576 (6.4)<br><br><br>124/367 (33.8)
Massa 2021[46]	BNT162b2	France	Kidney	Prospective	61	3 doses<br>(28 days)	Anti-spike Ab	The ARCHITECT SARS-CoV-2 IgG II Quant assay (Abbott)	38/61 (62.3)
Masset 2021[47]	BNT162b2	France	Kidney<br>Kidney-prancreas	Retrospective	456	2 doses<br>(1 month)<br><br>3 doses<br>(1 month)	Anti-spike Ab	The ARCHITECT SARS-CoV-2 IgG II Quant assay (Abbott) or Atellica chemiluminescence immunoassay (Siemens) or Elecsys anti-SARS-CoV-2 (Roche)	227/456 (49.8)<br><br><br>94/136 (69.1)
Masset 2021 (heterozygous vs homozygous vaccine) [48]	AZD1222-AZD1222 - mRNA vaccine or AZD1222-mRNA-mRNA vaccine<br><br>3 doses mRNA vaccine	France	Kidney	Prospective	28<br><br><br><br><br><br><br>56	3 doses<br>(1 month)	Anti-spike Ab	The ARCHITECT SARS-CoV-2 IgG II Quant assay (Abbott), Atellica chemiluminescence immunoassay (Siemens), Elecsys anti-SARS-CoV-2 (Roche)	21/28 (75)<br><br><br><br><br><br><br>38/56 (67.9)
Mazzola 2021[49]	BNT162b2	France	Kidney<br>Liver<br>Heart	Prospective	143	1 dose<br>(28 days)<br><br>2 doses (28 days)	Anti-spike Ab	The Alinity I SARS-CoV-2 IgG II assay (Abbott)	9/125 (7.2)<br><br><br>38/133 (28.6)
Medina-Pestana 2021[50]	Coronavac	Brazil	Kidney	Prospective	942	1 dose<br>(28 days)	Anti-spike Ab	The AdviseDx SARS-CoV-2 IgG II assay (Abbott)	143/942 (15.2)
Middleton 2021[51]	BNT162b2 or AZD1222	UK	Kidney	Retrospective	70	1 dose<br>(mean 40 days)	Anti-spike Ab	Siemens COV2T and Siemens COV2G	16/70 (22.9)
Midtvedth 2021[52]	BNT162b2	Norway	Kidney	Prospective	141	2 doses<br>(25-89 days)	Anti-spike, Anti-nucleocapsid Ab	Not reported	25/141 (17.7)
Miele 2021[53]	BNT162b2	Italy	Kidney<br>Lung<br>Liver<br>Heart	Prospective	16	2 doses<br>(at least 15 days)	Anti-spike Ab	LIAISON SARS-CoV-2 S1/S2 IgG chemiluminescent assay (DiaSorin)	6/16 (37.5)
Narasimhan 2021[54]	BNT162b2 or mRNA-1273	USA	Lung	Prospective	73	2 doses<br>(not reported)	Anti-spike Ab	The Alinity I SARS-CoV-2 IgG II assay (Abbott)	18/73 (24.7)
Noble 2021[55]	BNT162b2 or mRNA-1273	France	Kidney	Prospective	57	2 doses<br>(1 month)	Anti-spike Ab	Total SARS-CoV-2 antibodies ELISA assay (Beijing Wantai Biological Pharmacy Enterprise)	21/57 (36.8)
Pedersen 2021[56]	BNT162b2	Denmark	Kidney	Prospective	58	2 doses<br>(4 weeks)	Anti-spike Ab	VITROS Immunodiagnostic Products Anti-SARS-CoV-2 IgG (Ortho-Clinical Diagnostics), LIAISON	18/58 (31)

© 2022 Manothummetha K et al. *JAMA Network Open.*

								SARS-CoV-2 TrimericS IgG (DiaSorin)	
Peled 2021[57]	BNT162b2	Israel	Heart	Prospective	77	2 doses (21 days)	Anti-spike Ab<br><br>Neutralizing Ab	In house ELISA<br><br>SARS-CoV-2 pseudovirus (psSAR-2) neutralization assay (Gert Zimmer)	14/77 (18.2)<br><br>8/77 (10.4)
Peled 2021 (3 doses)[58]	BNT162b2	Israel	Heart	Prospective	96	3 doses (18 days)	Anti-spike Ab, Neutralizing Ab	In house ELISA	64/96 (67.7)
Prendecki 2021[59]	BNT162b2<br><br>AZD1222	UK	Kidney	Prospective	768	2 doses (median 31 days)	Anti-spike Ab	The ARCHITECT SARS-CoV-2 IgG II Quant assay (Abbott)	269/410 (66)<br><br>156/358 (44)
Quiroga 2021[60]	BNT162b2, mRNA-1273, Ad26.CoV.2, AZD1222	Spain	Kidney	Prospective	43	- 2 doses for BNT162b2, mRNA-1273, and AZD1222<br>- 1 dose for Ad26.CoV.2 (28 days)	Anti-spike Ab	COVID-19 Spike Quantitative Virclia IgG Monotest	9/43 (20.9)
Rabinowich 2021[61]	BNT162b2	Israel	Liver	Prospective	80	2 doses (10-20 days)	Anti-spike Ab	LIAISON SARS-CoV-2 S1/S2 IgG chemiluminescent assay (DiaSorin)	38/80 (47.5)
Rashidi-Alavijeh 2021[62]	BNT162b2	Germany	Liver	Prospective	43	2 doses (median 15 days)	Anti-spike Ab	LIAISON SARS-CoV-2 TrimericS IgG (DiaSorin)	34/43 (79.1)
Rahav 2021[63]	BNT162b2	Israel	Kidney<br>Heart<br>Liver	Prospective	227	2 doses (2-4 weeks)	Anti-spike Ab	The ARCHITECT SARS-CoV-2 IgG II Quant assay (Abbott), LIAISON SARS-CoV-2 S1/S2 IgG chemiluminescent assay (DiaSorin), Elecsys anti-SARS-CoV-2 (Roche), VIDAS SARS-COV-2 RBD IgG (BioMérieux), Siemens COV2T, Access SARS-CoV-2 RBD IgG assay (Beckman-Coulter)	90/227 (39.6)
Rincon-Arevalo 2021[64]	BNT162b2	Germany	Kidney	Prospective	40	2 doses (3-4 weeks)	Anti-spike Ab<br><br>Neutralizing Ab	EUROIMMUN SARS-CoV-2 ELISA assays<br><br>SARS-CoV-2 Surrogate Virus Neutralization Test (GenScript)	IgA 4/40 (10)<br>IgG 1/40 (2.5)<br><br>0/40 (0)
Rozen-Zvi 2021[65]	BNT162b2	Israel	Kidney	Prospective	308	2 doses (median 28 days)	Anti-spike Ab	SARS-CoV-2 IgG II Quant assay (Abbott)	112/308 (36.4)
Ruether 2021[66]	BNT162b2 or mRNA-1273 or AZD1222	Germany	Liver	Prospective	138	2 doses (median 29 days)	Anti-spike Ab	LIAISON SARS-CoV-2 TrimericS IgG (DiaSorin), Elecsys anti-SARS-CoV-2 (Roche)	84/135 (62)

© 2022 Manothummetha K et al. *JAMA Network Open.*

Russo 2021[67]	BNT162b2	Italy	Kidney	Retrospective	82	2 doses (median 43 days)	Anti-spike Ab	LIAISON SARS-CoV-2 S1/S2 IgG chemiluminescent assay (DiaSorin)	43/82 (52.4)
Sadioğlu 2021[68]	CoronaVac	Turkey	Kidney	Prospective	85	2 doses (median 31 days)	Anti-spike Ab	COVID-19 IgG – ELISA (DIA.PRO)	16/85 (18.8)
Sanders 2021[69]	mRNA-1273	Netherland	Kidney	Prospective	288	1 doses<br>(28 days)<br>2 doses<br>(28 days)	Anti-spike Ab	Not reported	61/288 (21.2)<br><br>164/288 (56.9)
Sattler 2021[70]	BNT162b2	Germany	Kidney	Prospective	39	2 doses<br>(8 days)	Anti-spike Ab<br><br>Neutralizing Ab	EUROIMMUN SARS-CoV-2 ELISA assays<br><br>SARS-CoV-2 Surrogate Virus Neutralization Test (GenScript)	IgG 1/39 (2.56%); IgA 4/39 (10.3)<br><br>0/39 (0)
Schmidt 2021[71]	Homologous or heterologous AZD1222 vaccine or mRNA vaccines (BNT162b2 or mRNA-1273)	Germany	Kidney<br>Heart<br>Lung<br>Liver<br>Liver and kidney	Prospective	40	1 dose<br>(13-30 days)<br><br>2 doses<br>(13-30 days)	Anti-spike Ab<br><br>Neutralizing Ab<br><br>Anti-spike Ab<br><br>Neutralizing Ab	EUROIMMUN SARS-CoV-2 ELISA assays<br><br>SARS-CoV-2-NeutraLISA (Euroimmun)<br><br>EUROIMMUN SARS-CoV-2 ELISA assays<br><br>SARS-CoV-2-NeutraLISA (Euroimmun)	2/38 (5.3)<br><br>0/38 (0)<br><br>12/34 (35.3)<br><br>10/34 (29.4)
Schramm 2021[72]	BNT162b2	Germany	Lung<br>Heart<br>Heart-lung	Prospective	50	1 dose<br>(21 days)<br><br>2 doses<br>(21 days)	Anti-spike Ab<br><br>Neutralizing Ab<br><br>Anti-spike Ab<br><br>Neutralizing Ab	SARS-CoV-2 IgG II Quant assay (Abbott), EUROIMMUN SARS-CoV-2 ELISA assays, Elecsys anti-SARS-CoV-2 (Roche)<br><br>SARS-CoV-2 Surrogate Virus Neutralization Test (GenScript)<br><br>SARS-CoV-2 IgG II Quant assay (Abbott), EUROIMMUN SARS-CoV-2 ELISA assays, Elecsys anti-SARS-CoV-2 (Roche)<br><br>SARS-CoV-2 Surrogate Virus Neutralization Test (GenScript)	2/50 (4)<br><br>0/50 (0)<br><br>5/50 (10)<br><br>0/50 (0)
Schrezenmeier 2021 [73]	AZD1222 or BNT162b2 after 2 doses of BNT162b2	Germany	Kidney	Prospective	25	3 doses<br>(19-27 days)	Anti-spike Ab<br>Neutralizing Ab	EUROIMMUN SARS-CoV-2 ELISA assay<br>SARS-CoV-2 Surrogate Virus Neutralization Test (GenScript)	9/25 (36)<br>7/20 (35)
Shostak 2021[74]	BNT162b2	Israel	Lung	Prospective	168	2 doses<br>(14-21 days)	Anti-spike Ab	SARS-CoV-2 IgG II Quant assay (Abbott)	31/137 (22.6)

© 2022 Manothummetha K et al. *JAMA Network Open.*

Stumpf 2021 (Sub-analysis of DIAVAC)[75]	BNT162b2	Germany	Kidney	Prospective	71	3 doses<br>(4 weeks)	Anti-spike Ab	EUROIMMUN SARS-CoV-2 ELISA assays	39/71 (55)
Stumpf 2021[76]	BNT162b2 or mRNA-1273	Germany	Kidney	Prospective	368	1 dose<br>(3-4 weeks)<br><br>2 doses<br>(8 weeks after the first dose)	Anti-spike Ab<br><br>Anti-spike Ab<br><br>Neutralizing Ab	EUROIMMUN SARS-CoV-2 ELISA assays<br><br>EUROIMMUN SARS-CoV-2 ELISA assays<br><br>SARS-CoV-2-NeutraLISA (Euroimmun)	IgA or IgG 11/144 (7.6)<br><br>IgA or IgG 140/333 (42)<br><br>79/333 (23.7)
Thuluvath 2021[77]	mRNA (BNT162b2 or mRNA-1273) or Janssen	USA	Liver	Prospective	62	2 doses<br>(4 weeks)	Anti-spike Ab	Elecsys anti-SARS-CoV-2 (Roche)	24/62 (38.7)
Villanego 2021[78]	BNT162b2 or mRNA-1273	Spain	Kidney	Prospective	91	2 doses<br>(1 month)	Anti-spike Ab	SARS-CoV-2 IgG chemiluminescent microparticle immunoassay (Abbott)	57/91 (62.6)
Werbel 2021[79]	2 doses of BNT162b2 or mRNA-1273 and dose 3 with BNT162b2 or mRNA-1273 or or Janssen 3 doses	USA	Mixed	Prospective	30	3 doses<br>(14 days)	Anti-spike Ab	EUROIMMUN anti-S1 IgG assay, Elecsys anti-SARS-CoV-2 (Roche)	14/30 (46.7)
Westhoff 2021[80]	2 doses of BNT162b2 followed by a dose of mRNA-1273	Germany	Kidney	Prospective	10	3 doses (2 weeks)	Anti-spike Ab	Elecsys anti-SARS-CoV-2 (Roche)	6/10 (60)
Wijtvliet 2021[81]	mRNA-1273<br><br>BNT162b2	Belgium	Kidney	Prospective	138	1 dose<br>(16-21 days)<br>2 doses<br>(21-35 days)<br>1 dose<br>(16-21 days)<br>2 doses<br>(21-35 days)	Anti-spike Ab	Luminex	11/44 (25)<br><br>32/42 (76.2)<br><br>8/94 (8.5)<br><br>51/91 (56)
Yi 2021[82]	BNT162b2 or mRNA-1273	USA	Kidney	Prospective	145	1 dose<br>(not reported)	Anti-spike Ab	Not reported	8/145 (5.5)

Ab: antibodies; IQR: interquartile range

© 2022 Manothummetha K et al. *JAMA Network Open.*

eTable 3. Grading of Recommendation Assessment, Development and Evaluation (GRADE) for Potential Factors Associated With Diminished Humoral Immune Response

Potential factors	Number of studies (number of cohorts)	Number of participants	Univariate			Multivariate			GRADE factors								
			-	0	+	-	0	+	Study limitations	Inconsistency	Indirectness	Imprecision	Publication bias	Moderate/large effect	Dose effect	Effect of plausible residual confounding	Overall Quality
Antimetabolite	25	3,664	15	6	0	4	0	0	×	×	✓	✓	×	✓	×	×	++
Anti-thymocyte globulin	5	721	0	4	0	0	1	0	×	✓	✓	×	×	✓	×	×	++
Calcineurin inhibitor	17	2,615	1	13	1	0	2	0	×	✓	✓	×	✓	×	×	×	++
Deceased donor status	10	1,891	1	9	0	0	0	0	×	✓	✓	✓	✓	×	×	×	+++
Male	26	4,414	0	25	0	0	1	0	×	✓	✓	×	✓	×	×	×	++
mTOR inhibitor	21	3,314	2	15	1	0	2	1	×	✓	✓	×	×	×	×	×	+
Rituximab	5	861	0	4	0	1	0	0	×	✓	✓	✓	✓	✓	×	×	+++
Age	10	1,955	7	3	0	0	0	0	×	×	✓	✓	×	×	×	×	+
BMI	9	1,971	0	9	0	0	0	0	×	✓	✓	×	×	×	×	×	+
Lymphocyte count	4	413	0	3	1	0	0	0	×	✓	✓	×	×	×	×	×	+
Duration after transplantation	9	1,667	0	7	2	0	0	0	×	×	✓	✓	✓	×	×	×	++

BMI: Body Mass Index

Note: For uni- and multivariate analyses: +, number of significant effects with a positive value; 0, number of non-significant effects; -, number of significant effects with a negative value. For GRADE factors: ✓, no serious limitations; ×, serious limitations (or not present for moderate/large effect size, dose effect); unclear, unable to rate item based on available information. For overall quality of evidence: +, very low; ++, low; +++, moderate; ++++, high. **Reference:** Huguet et al. Systematic Reviews 2013, 2:71.

© 2022 Manothummetha K et al. *JAMA Network Open.*

eTable 4. Humoral Immune Response Rates After 2 Doses of mRNA Vaccines by the Different Testing

Methods	Percentage of average seroconversion rates (range)	Numbers of the primary studies
Anti-spike antibodies		
Alinity I SARS-CoV-2 IgG II assay (Abbott)	29.2 (24.7 – 40.5)	3 studies[14,49,54]
ARCHITECT SARS-CoV-2 IgG II Quant assay (Abbott)	46.7 (27.7 – 65.6)	9 studies[3,5,9,19,32,44,59,65,74]
SARS-CoV-2 antibodies ELISA assay (Beijing Wantai Biological Pharmacy Enterprise)	39.1 (36.8 – 40.4)	2 studies[39,55]
LIAISON SARS-CoV-2 S1/S2 IgG chemiluminescent assay (DiaSorin)	59.8 (10 - 74.8)	6 studies[25,28,29,53,61,67]
LIAISON SARS-CoV-2 TrimericS IgG (DiaSorin)	42.9 (4.1 - 79.1)	6 studies[17,20,22,37,42,62]
EUROIMMUN SARS-CoV-2 ELISA assay	28.6 (0 - 42)	6 studies[33,38,41,64,70,76]
Luminex	47.2 (29.9 – 76.2)	2 study[18,81]
Elecsys anti-SARS-CoV-2 (Roche)	41.5 (34.6 - 49.4)	3 studies[27,307]
Siemens COV2T and Siemens COV2G	64.4 (56.5 - 70.7)	1 study (2 cohorts)[35]
Quest Diagnostics	47.8	1 study[83]
Neutralizing antibodies		
SARS-CoV-2 Surrogate Virus Neutralization Test (GenScript)	25.9 (0 – 26.9)	4 studies[30,64,70,72]
SARS-CoV-2 pseudovirus (psSAR-2) neutralization assay (Gert Zimmer)	25.4 (10.4 – 35)	2 studies[36,57]
SARS-CoV-2-NeutraLISA (Euroimmun)	23.7	1 study[76]

© 2022 Manothummetha K et al. *JAMA Network Open.*

eTable 5. Humoral Immune Response by the COVID-19 Vaccine Types and Doses

Study	Antibody measurement	Methods	Timing of Ab testing	Responders/Total	Seroconversion rate
BNT162b2 vaccine (1 dose)					
Ben Zadok 2021[3]	Anti-spike Ab	The ARCHITECT SARS-CoV-2 IgG II Quant assay (Abbott)	21 days	6/39	15.38%
Bertrand 2021[3]	Anti-spike Ab	The ARCHITECT SARS-CoV-2 IgG II Quant assay (Abbott)	3 weeks	1/45	2.22%
Chavarot 2021[15]	Anti-spike Ab	SARS-CoV-2 IgG II Quant assay (Abbott) or total SARS-CoV-2 antibodies ELISA assay (Beijing Wantai Biological Pharmacy Enterprise)	28 days	2/101	1.98%
Firket 2021[25]	Anti-spike Ab	LIAISON SARS-CoV-2 S1/S2 IgG chemiluminescent assay (DiaSorin)	21 days	0/10	0%
Del Bello 2021[23]	Anti-spike Ab	Mainly total SARS-CoV-2 antibodies ELISA assay (Beijing Wantai Biological Pharmacy Enterprise)	Before second dose	20/396	5.05%
Georgery 2021[26]	Anti-spike Ab	Elecsys anti-SARS-CoV-2 (Roche)	28 days	3/78	3.85%
Havlin 2021[33]	Anti-spike Ab	Euroimmun, TestLine Clinical Diagnostics, LIAISON SARS-CoV-2 TrimericS IgG (DiaSorin)	Before second dose	0/46	0.00%
Kamar 2021[39]	Anti-spike Ab	SARS-CoV-2 antibodies ELISA assay (Beijing Wantai Biological Pharmacy Enterprise)	Before 2nd dose	0/101	0%
Mazzola 2021[49]	Anti-spike Ab	The Alinity I SARS-CoV-2 IgG II assay (Abbott)	28 days	9/125	7.2%
Schramm 2021[72]	Anti-spike Ab	SARS-CoV-2 IgG II Quant assay (Abbott), EUROIMMUN SARS-CoV-2 ELISA assays, Elecsys anti-SARS-CoV-2 (Roche)	21 days	2/50	4.00%
	Neutralizing Ab	SARS-CoV-2 Surrogate Virus Neutralization Test (GenScript)	21 days	0/50	0%
Wijtvliet 2021[81]	Anti-spike Ab	Luminex	16-21 days	8/94	8.51%
mRNA-1273 vaccine (1 dose)					

© 2022 Manothummetha K et al. *JAMA Network Open.*

Benotmane 2021 (1 dose) [4]	Anti-spike Ab	The ARCHITECT SARS-CoV-2 IgG II Quant assay (Abbott)	28 days	26/241	10.79%
Herrera 2021 (Heart) [35]	Anti-spike Ab	Siemens COV2T and Siemens COV2G	4 weeks	5/46	10.87%
Herrera 2021 (Liver) [35]	Anti-spike Ab	Siemens COV2T and Siemens COV2G	4 weeks	22/58	37.93%
Hall 2021[30]	Anti-spike Ab	Elecsys anti-SARS-CoV-2 (Roche)	4 weeks	6/121	4.96%
	Neutralizing Ab	SARS-CoV-2 Surrogate Virus Neutralization Test (GenScript)	4 weeks	7/119	5.88%
Sanders 2021[69]	Anti-spike Ab	Not reported	28 days	61/288	21.18%
Wijtvliet 2021 [81]	Anti-spike Ab	Luminex	16-21 days	11/44	25.00%
Mixed mRNA (1 dose)					
Boyarsky 2021[11]	Anti-spike Ab	Elecsys anti-SARS-CoV-2 (Roche) or EUROIMMUN SARS-CoV-2 ELISA assay	Median 21 days	76/436	17.43%
Marion 2021[45]	Anti-spike Ab	Mainly total SARS-CoV-2 antibodies ELISA assay (Beijing Wantai Biological Pharmacy Enterprise)	Before second dose	37/576	6.42%
Stumpf 2021[76]	Anti-spike Ab	EUROIMMUN SARS-CoV-2 ELISA assays	3-4 weeks	11/144	7.64%
Yi 2021[82]	Anti-spike Ab	Not reported	Not reported	8/145	5.52%
BNT162b2 vaccine (2 doses)					
Bergman 2021 [7]	Anti-spike Ab	Elecsys anti-SARS-CoV-2 (Roche), EUROIMMUN SARS-CoV-2 ELISA assays	14 days	36/83	43.37%
Ben Zadok 2021[3]	Anti-spike Ab	The ARCHITECT SARS-CoV-2 IgG II Quant assay (Abbott)	35-40 days (after first dose)	18/37	48.65%
Bertrand 2021[5]	Anti-spike Ab	The ARCHITECT SARS-CoV-2 IgG II Quant assay (Abbott)	4 weeks	65/235	27.66%
Chavarot 2021[15]	Anti-spike Ab	SARS-CoV-2 IgG II Quant assay (Abbott) or total SARS-CoV-2 antibodies ELISA assay (Beijing Wantai Biological Pharmacy Enterprise)	1 month	2/35	5.71%

© 2022 Manothummetha K et al. *JAMA Network Open.*

Cholankeril 2021[83]	Anti-spike Ab	Quest Diagnostics	Median (IQR) 81 (72-90) days (after first dose)	33/69	47.83%
Danthu 2021[20]	Anti-spike Ab	LIAISON SARS-CoV-2 TrimericS IgG (DiaSorin)	36 days	3/74	4.05%
Davidov 2021[21]	Anti-spike Ab	Not reported	Median (IQR) 36 (17-52) days	55/76	72.37%
Del Bello 2021[23]	Anti-spike Ab	Mainly total SARS-CoV-2 antibodies ELISA assay (Beijing Wantai Biological Pharmacy Enterprise)	Before third dose	164/396	41.41%
Ducloux 2021[24]	Anti-spike Ab	SARS-CoV-2 immunoassay (Abbott)	2.5 months	81/153	52.94%
Firket 2021[25]	Anti-spike Ab	LIAISON SARS-CoV-2 S1/S2 IgG chemiluminescent assay (DiaSorin)	15 days	1/10	10.00%
Georgery 2021 (2 doses) [27]	Anti-spike Ab	Elecsys anti-SARS-CoV-2 (Roche)	28 days	39/79	49.37%
Grupper 2021[28]	Anti-spike Ab	LIAISON SARS-CoV-2 S1/S2 IgG chemiluminescent assay (DiaSorin)	10-20 d	51/136	37.50%
Guarino 2021[29]	Anti-spike Ab	LIAISON SARS-CoV-2 S1/S2 IgG chemiluminescent assay (DiaSorin)	4 weeks	273/365	74.79%
Haskin 2021[32]	Anti-spike Ab	The ARCHITECT SARS-CoV-2 IgG II Quant assay (Abbott)	1 week	24/38	63.16%
Havlin 2021[33]	Anti-spike Ab	EUROIMMUN SARS-CoV-2 ELISA assays	4-6 weeks	0/21	0.00%
Hod 2021[36]	Anti-spike Ab	In-house ELISA	2-4 weeks	52/120	43.33%
	Neutralizing Ab	SARS-CoV-2 pseudovirus (psSAR-2) neutralization assay, (Gert Zimmer)	2-4 weeks	42/120	35.00%
Holden 2021[38]	Anti-spike Ab	EUROIMMUN SARS-CoV-2 ELISA assays	Median (IQR) 5.6 (5.1-6.3) weeks	28/80	35%
Kamar 2021[39]	Anti-spike Ab	SARS-CoV-2 antibodies ELISA assay (Beijing Wantai Biological Pharmacy Enterprise)	Before third dose	40/99	40.40%
Korth 2021[42]	Anti-spike Ab	LIAISON SARS-CoV-2 TrimericS IgG (DiaSorin)	14 days	5/23	21.74%

© 2022 Manothummetha K et al. *JAMA Network Open.*

Marinaki 2021[44]	Anti-spike Ab	The ARCHITECT SARS-CoV-2 IgG II Quant assay (Abbott)	Median 10 days	20/34	58.82%
Masset 2021[47]	Anti-spike Ab	The ARCHITECT SARS-CoV-2 IgG II Quant assay (Abbott) or Atellica chemiluminescence immunoassay (Siemens) or Elecsys anti-SARS-CoV-2 (Roche)	1 month	227/456	49.78%
Mazzola 2021[49]	Anti-spike Ab	The Alinity I SARS-CoV-2 IgG II assay (Abbott)	28 days	38/133	28.57%
Miele 2021[53]	Anti-spike Ab	LIAISON SARS-CoV-2 S1/S2 IgG chemiluminescent assay (DiaSorin)	At least 15 days	6/16	37.5%
Pederson 2021[56]	Anti-spike Ab	VITROS Immunodiagnostic Products Anti-SARS-CoV-2 IgG (Ortho-Clinical Diagnostics), LIAISON SARS-CoV-2 TrimericS IgG (DiaSorin)	4 weeks	18/58	31.03%
Midtvedth 2021[52]	Anti-spike Ab, Anti-nucleocapsid Ab	Not reported	25-89 d	25/141	17.73%
Peled 2021[57]	Anti-spike Ab	In-house ELISA	21 days	14/77	18.18%
	Neutralizing Ab	SARS-CoV-2 pseudovirus (psSAR-2) neutralization assay (Gert Zimmer)	21 days	8/77	10.39%
Prendecki 2021[59]	Anti-spike Ab	The ARCHITECT SARS-CoV-2 IgG II Quant assay (Abbott)	Median 31 days	269/410	65.61%
Rabinowich 2021[61]	Anti-spike Ab	LIAISON SARS-CoV-2 S1/S2 IgG chemiluminescent assay (DiaSorin)	10-20 days	38/80	47.50%
Rashidi-Alavijeh 2021[62]	Anti-spike Ab	LIAISON SARS-CoV-2 TrimericS IgG (DiaSorin)	Median (IQR) 15 days (12-24)	34/43	79.07%
Rahav 2021 [63]	Anti-spike Ab	The ARCHITECT SARS-CoV-2 IgG II Quant assay (Abbott), LIAISON SARS-CoV-2 S1/S2 IgG chemiluminescent assay (DiaSorin), Elecsys anti-SARS-CoV-2 (Roche), VIDAS SARS-COV-2 RBD IgG (BioMérieux), Siemens COV2T, Access SARS-CoV-2 RBD IgG assay (Beckman-Coulter)	2-4 weeks	90/227	39.65%
Rincon-Arevalo 2021[64]	Anti-spike Ab (IgA)	EUROIMMUN SARS-CoV-2 ELISA assays	3-4 weeks	4/40	10.00%

© 2022 Manothummetha K et al. *JAMA Network Open.*

Rincon-Arevalo 2021[64]	Anti-spike Ab (IgG)	EUROIMMUN SARS-CoV-2 ELISA assays	3-4 weeks	1/40	2.50%
	Neutralizing Ab	SARS-CoV-2 Surrogate Virus Neutralization Test (GenScript)	3-4 weeks	0/40	0.00%
Rozen-Zvi 2021[65]	Anti-spike Ab	The ARCHITECT SARS-CoV-2 IgG II Quant assay (Abbott)	2-4 weeks	112/308	36.36%
Russo 2021[67]	Anti-spike Ab	LIAISON SARS-CoV-2 S1/S2 IgG chemiluminescent assay (DiaSorin)	Median 43 days	43/82	52.44%
Sattler 2021[70]	Anti-spike Ab (IgG)	EUROIMMUN SARS-CoV-2 ELISA assays	Mean (SD) 8 (1) days	1/39	2.56%
Sattler 2021[70]	Anti-spike Ab (IgA)	EUROIMMUN SARS-CoV-2 ELISA assays	Mean (SD) 8 (1) days	4/39	10.26%
	Neutralizing Ab	SARS-CoV-2 Surrogate Virus Neutralization Test (GenScript)	Mean (SD) 8 (1) days	0/39	0.00%
Schramm 2021[72]	Anti-spike Ab	SARS-CoV-2 IgG II Quant assay (Abbott), EUROIMMUN SARS-CoV-2 ELISA assays, Elecsys anti-SARS-CoV-2 (Roche)	21 days	5/50	10.00%
	Neutralizing Ab	SARS-CoV-2 Surrogate Virus Neutralization Test (GenScript)	21 days	0/50	0%
Shostak 2021[74]	Anti-spike Ab	The ARCHITECT SARS-CoV-2 IgG II Quant assay (Abbott)	14-21 days	31/137	22.63%
Wijtvliet 2021[81]	Anti-spike Ab	Luminex	21-35 days	51/91	56.04%
mRNA-1273 vaccine (2 doses)					
Benotmane 2021 (2nd dose) [5]	Anti-spike Ab	The ARCHITECT SARS-CoV-2 IgG II Quant assay (Abbott)	28 days	98/204	48.04%
Crespo 2021[17]	Anti-spike Ab	LIAISON SARS-CoV-2 TrimericS IgG (DiaSorin)	28 days	57/90	63.33%
Cucchiari 2021[18]	Anti-spike Ab	Luminex	2 weeks	35/117	29.91%
Hall 2021[30]	Anti-spike Ab	Elecsys anti-SARS-CoV-2 (Roche)	4-6 weeks	38/110	34.55%
	Neutralizing Ab	SARS-CoV-2 Surrogate Virus Neutralization Test (GenScript)	4-6 weeks	29/108	26.85%

© 2022 Manothummetha K et al. *JAMA Network Open.*

Herrera 2021 (Heart) [35]	Anti-spike Ab	Siemens COV2T and Siemens COV2G	4 weeks	26/46	56.52%
Herrera 2021 (Liver) [35]	Anti-spike Ab	Siemens COV2T and Siemens COV2G	4 weeks	41/58	70.69%
Sanders 2021[69]	Anti-spike Ab	Not reported	28 days	164/288	56.94%
Wijtvliet 2021[81]	Anti-spike Ab	Luminex	21-35 days	32/42	76.19%
Mixed mRNA (2 doses)					
Boyarsky 2021 (2 doses)	Anti-spike Ab	Elecsys anti-SARS-CoV-2 (Roche)	Median 29 days	357/658	54.26%
Cao 2021[14]	Anti-spike Ab	The Alinity I SARS-CoV-2 IgG II assay (Abbott)	Not reported	15/37	40.54%
Dębska-Ślizień 2021[22]	Anti-spike Ab	LIAISON SARS-CoV-2 TrimericS IgG (DiaSorin)	14-21 days	73/142	51.41%
D'Offizi 2021 [19]	Anti-spike Ab	The ARCHITECT SARS-CoV-2 IgG II Quant assay (Abbott)	2 weeks	47/61	77.05%
	Neutralizing Ab	SARS-CoV2/Human/ITA/PAVIA10734/2020 provided by F. Baldanti, Pavia	2 weeks	29/61	47.54%
Hoffman 2021[37]	Anti-spike Ab	LIAISON SARS-CoV-2 TrimericS IgG (DiaSorin)	6 weeks	22/80	27.50%
Kantauskaite 2021[41]	Anti-spike Ab	Anti-SARS-CoV-2- QuantiVacELISA (Euroimmun)	Mean 14 days	56/225	24.89%
Marion 2021[45]	Anti-spike Ab	Mainly total SARS-CoV-2 antibodies ELISA assay (Beijing Wantai Biological Pharmacy Enterprise)	4 weeks	124/367	33.79%
Narasimhan 2021[54]	Anti-spike Ab	The Alinity I SARS-CoV-2 IgG II assay (Abbott)	Not reported	18/73	24.66%
Noble 2021 [55]	Anti-spike Ab	Total SARS-CoV-2 antibodies ELISA assay (Beijing Wantai Biological Pharmacy Enterprise)	1 month	21/57	36.84%
Stumpf 2021[76]	Anti-spike Ab	EUROIMMUN SARS-CoV-2 ELISA assays	8 weeks	140/333	42.04%
	Neutralizing Ab	SARS-CoV-2-NeutraLISA (Euroimmun)	8 weeks	79/333	23.72%

© 2022 Manothummetha K et al. *JAMA Network Open.*

Villanego 2021[78]	Anti-spike Ab	SARS-CoV-2 IgG chemiluminescent microparticle immunoassay (Abbott)	1 month	57/91	62.64%
BNT162b2 vaccine (3 doses)					
Bertrand 2021 (3 doses) [10]	Anti-spike Ab	The ARCHITECT SARS-CoV-2 IgG II Quant assay (Abbott)	1 month	49/80	61.25%
Del Bello 2021[23]	Anti-spike Ab	Mainly total SARS-CoV-2 antibodies ELISA assay (Beijing Wantai Biological Pharmacy Enterprise)	4 weeks	269/396	67.93%
Havlin 2021 (3 doses) [34]	Anti-spike Ab	TestLine Clinical Diagnostics	3 weeks	2/15	13.33%
Kamar 2021 [39]	Anti-spike Ab	SARS-CoV-2 antibodies ELISA assay (Beijing Wantai Biological Pharmacy Enterprise)	4 weeks	67/99	67.68%
Massa 2021 [46]	Anti-spike Ab	The ARCHITECT SARS-CoV-2 IgG II Quant assay (Abbott)	28 days	38/56	62.30%
Masset 2021[47]	Anti-spike Ab	The ARCHITECT SARS-CoV-2 IgG II Quant assay (Abbott), Atellica chemiluminescence immunoassay (Siemens), Elecsys anti-SARS-CoV-2 (Roche)	1 month	94/136	69.12%
Peled 2021 (3 doses) [58]	Anti-spike Ab, Neutralizing Ab	In house ELISA	18 days	64/96	66.67%
Stumpf 2021 (Subanalysis of DIAVAC))[75]	Anti-spike Ab	EUROIMMUN SARS-CoV-2 ELISA assays	4 weeks	39/71	55%
mRNA-1273 vaccine (3 doses)					
Benotmane 2021 (3 doses) [10]	Anti-spike Ab	The ARCHITECT SARS-CoV-2 IgG II Quant assay (Abbott)	Median (IQR) 28 days (27-33)	78/159	49.06%
Hall 2021 (3 doses) [31]	Anti-spike Ab	Elecsys anti-SARS-CoV-2 (Roche)	4 weeks	33/60	55%
	Neutralizing Ab	SARS-CoV-2 Surrogate Virus Neutralization Test (GenScript)	4 weeks	36/60	60%
Mixed mRNA (3 doses)					

© 2022 Manothummetha K et al. *JAMA Network Open.*

Westhoff 2021[80]	Anti-spike Ab	Elecsys anti-SARS-CoV-2 (Roche)	2 weeks	6/10	60%
BNT162b2 vaccine (4 doses)					
Kamar 2021 (4th dose)[40]	Anti-spike Ab	SARS-CoV-2 antibodies ELISA assay (Beijing Wantai Biological Pharmacy Enterprise)	1 month	18/37	48.65%
mRNA (4 doses)					
Alejo 2021[1]	Anti-spike Ab	Elecsys anti-SARS-CoV-2 (Roche) or EUROIMMUN SARS-CoV-2 ELISA assay	2-6 weeks	15/18	83.33%
Janssen (1 dose)					
Boyarsky 2021[13]	Anti-spike Ab	Elecsys anti-SARS-CoV-2 (Roche)	Median (IQR) 33 days (31-44)	2/12	16.67%
CoronaVac (1 dose)					
Medina-Pestana 2021[50]	Anti-spike Ab	The AdviseDx SARS-CoV-2 IgG II assay (Abbott)	28 days	143/942	15.18%
CoronaVac (2 doses)					
Medina-Pestana 2021[50]	Anti-spike Ab	The AdviseDx SARS-CoV-2 IgG II assay (Abbott)	Median 40 (IQR 32-46) days	368/856	42.99%
Sadioğlu 2021[68]	Anti-spike Ab	COVID-19 IgG – ELISA (DIA.PRO)	Median 31 days	16/85	18.82%
Other vaccine platforms					
Azzi 2021 (mRNA vaccines 2 doses or Janssen 1 dose) [2]	Anti-spike Ab	VITROS Immunodiagnostic Products Anti-SARS-CoV-2 IgG (Ortho-Clinical Diagnostics)	Median 28 days	24/76	31.58%
Middleton 2021 (AZD1222 or BNT162b2 1 dose) [51]	Anti-spike Ab	Siemens COV2T and Siemens COV2G	Mean 40 days	16/70	22.86%
Prendecki 2021 (AZD1222 2 doses) [59]	Anti-spike Ab	The ARCHITECT SARS-CoV-2 IgG II Quant assay (Abbott)	Median 31 days	156/358	43.58%

© 2022 Manothummetha K et al. *JAMA Network Open.*

Quiroga 2021 (BNT162b2 or mRNA-1273 or AZD1222 2 doses and Ad26.CoV.2 1 dose) [60]	Anti-spike Ab	COVID-19 Spike Quantitative Virclia IgG Monotest	28 days	9/43	20.93%
Masset 2021 (AZD1222 2 doses followed by 1 dose of mRNA vaccine or AZD1222 1 dose followed by 2 doses of mRNA vaccine) [48]	Anti-spike Ab	Elecsys anti-SARS-CoV-2 (Roche), The ARCHITECT SARS-CoV-2 IgG II Quant assay (Abbott), LIAISON SARS-CoV-2 TrimericS IgG (DiaSorin)	1 month	21/28	75.00%
Ruether 2021[66]	Anti-spike Ab	LIAISON SARS-CoV-2 TrimericS IgG (DiaSorin), Elecsys anti-SARS-CoV-2 (Roche)	Median 29 days	84/135	62.22%
Schrezenmeier 2021 [73]	Anti-spike Ab	EUROIMMUN SARS-CoV-2 ELISA assays	19-27 days	9/25	36.00%
	Neutralizing Ab	SARS-CoV-2 Surrogate Virus Neutralization Test (GenScript)	19-27 days	7/20	35.00%
Schmidt 2021 (Homologous or heterologous AZD1222 vaccine or mRNA vaccines 2 doses) [71]	Anti-spike Ab	EUROIMMUN SARS-CoV-2 ELISA assays	13-30 days	1 dose: 2/38<br>2 doses: 12/34	5.26%<br>35.29%
	Neutralizing Ab	SARS-CoV-2-NeutraLISA (Euroimmun)	13-30 days	1 dose: 0/38<br>2 doses: 10/34	0%<br>29.41%
Thuluvath 2021 (mRNA vaccines 2 doses or Janssen 1 dose) [77]	Anti-spike Ab	Elecsys anti-SARS-CoV-2 (Roche)	4 weeks	24/62	39%
Werbel 2021 (mRNA or Janssen 3 doses) [79]	Anti-spike Ab	EUROIMMUN anti-S1 IgG assay, Elecsys anti-SARS-CoV-2 (Roche)	14 days	14/30	46.67%

Ab: antibodies; IQR: interquartile range; SD: standard deviation

© 2022 Manothummetha K et al. *JAMA Network Open.*

eTable 6. Participants and the Effect Estimates of Potential Risk Factors for Diminished Humoral Immune Response in Each Study

Study name	Risk factor	Subject with risk factor		Published measure of association	Calculated odds ratios from raw data	Published measure of association between risk factor and immune response		Confounding risk adjustment in multivariable analysis[a]
		**Response**	**No response**			**Univariable (95% CI)**	**Multivariable (95% CI)**	
BNT162b2 vaccine								
Bergman 2021 [7]	Antimetabolite	2/32	18/21	OR	0.01 (0.002, 0.07)	0.013 (0.003-0.051)[b]	0.011 (0.002-0.048)[b, c]	Creatinine baseline
Cholankeril 2021 [83]	Antimetabolite	11/33	14/36		0.79 (0.29, 2.11)			
	CNI	29/33	35/36		0.21 (0.02, 1.96)			
	Male	25/33	23/36		1.77 (0.62, 5.03)			
Davidov 2021 [21]	Antimetabolite	7/55	10/21		0.16 (0.05, 0.52)			
	Male	33/55	10/21		1.65 (0.6, 4.54)			
	mTORi	6/55	5/21		0.39 (0.11, 1.46)			
Ducloux 2021[24]	Antimetabolite	26/ 81	55/ 72	OR	0.15 (0.07, 0.3)	0.13 (0.06-0.27)	0.14 (0.05-0.42)	Transplant duration, eGFR, CNI, mTORi, MPA, CD4 cell count
	CNI	58/ 81	65/ 72	OR	0.27 (0.11, 0.68)	0.27 (0.11-0.68)	1.24 (0.24-6.38)	
	Male	48/ 81	45/ 72	OR	0.87 (0.46, 1.67)	1.4 (0.71-2.77)		
	mTOR	29/ 81	4/ 72	OR	9.48 (3.14, 28.65)	9.06 (2.99-24.48)	1.71 (0.32-9.17)	
Grupper 2021[28]	Antimetabolite	32/ 51	72/ 85	OR	0.3 (0.13, 0.69)		0.68 (0.44-0.79)[b, c]	Age, time post-first transplantation, eGFR, triple immunosuppression, high-dose corticosteroid last 12 months, low dose prednisone, maintenance MMF
	ATG	1/ 51	9/ 85		0.17 (0.02, 1.37)			
	CNI	46/ 51	77/ 85		0.96 (0.3, 3.1)			
	Deceased donor	14/ 51	38/ 85		0.47 (0.22, 0.99)			
	Male	34/ 51	53/ 85		1.21 (0.58, 2.5)			
	mTOR	5/ 51	5/ 85		1.74 (0.48, 6.33)			
	Rituximab	1/ 51	3/ 85		0.55 (0.06, 5.4)			
Guarino 2021[29]	Antimetabolite	83/ 273	49/ 92		0.38 (0.24, 0.62)			
	CNI	227/ 273	72/ 92		1.37 (0.76, 2.47)			
	Male	206/ 273	73/ 92		0.8 (0.45, 1.42)			
	mTOR	55/ 273	30/ 92		0.52 (0.31, 0.88)			
Haskin 2021[32]	ATG	2/ 24	3/ 14		0.33 (0.05, 2.3)			
	Deceased donor	10/ 24	5/ 14		1.29 (0.33, 5.02)			
	Male	18/ 24	7/ 14		3 (0.74, 12.13)			
	Rituximab	2/ 24	7/ 14	OR	0.09 (0.02, 0.54)		0.08 (0.013-0.48)	Not reported
Hod 2021[36]	Antimetabolite	27/ 42	69/ 78		0.24 (0.09, 0.6)			
	CNI	39/ 42	72/ 78		1.08 (0.26, 4.57)			
	Deceased donor	4/ 42	18/ 78		0.35 (0.11, 1.12)			
	Male	32/ 42	64/ 78		0.7 (0.28, 1.75)			
	mTOR	3/ 42	4/ 78		1.42 (0.3, 6.68)			
Holden 2021[38]	Antimetabolite	23/ 28	52/ 52		0.04 (0.002, 0.77)			
	CNI	28/ 28	47/ 52		6.6 (0.35, 123.86)			
	Male	14/ 28	30/ 52		0.73 (0.29, 1.85)			
	mTOR	1/ 28	1/ 52		1.89 (0.11, 31.4)			
Korth 2021[42]	Antimetabolite	3/ 5	16/ 18		0.19 (0.02, 1.9)			
	CNI	4/ 5	14/ 18		1.14 (0.1, 13.34)			
	Male	2/ 5	9/ 18		0.67 (0.09, 4.99)			
	mTOR	2/ 5	4/ 18		2.33 (0.28, 19.17)			
Masset 2021[47]	Antimetabolite	145/ 227	180/ 229	OR	0.48 (0.32, 0.73)		0.17 (0.09-0.33)[b]	Recipient age at vaccination, transplantation ≤ 4 years, allograft function by MDRD equation, CNI, mTOR, antimetabolite, steroids, lymphocyte count < 1500/mm^3
	CNI	178/ 227	191/ 229	OR	0.72 (0.45, 1.16)		0.65 (0.3-1.37)[b]	
	Deceased donor	180/ 227	196/ 229		0.65 (0.4, 1.05)			
	Male	145/ 227	130/ 229		1.35 (0.92, 1.96)			
	mTOR	48/ 227	20/ 229	OR	2.8 (1.6, 4.9)		1.37 (0.62-3.03)[b]	

© 2022 Manothummetha K et al. *JAMA Network Open.*

Mazzola 2021[49]	Antimetabolite	24/ 38	71/ 95		0.58 (0.26, 1.3)			Not reported
	CNI	31/ 38	78/ 95		0.97 (0.37, 2.56)			
	Male	28/ 38	64/ 95		1.36 (0.59, 3.14)			
	mTOR	8/ 38	18/ 95		1.14 (0.45, 2.9)			
Midtvedth 2021[52]	Antimetabolite	9/ 25	106/ 116		0.05 (0.02, 0.15)			
	Deceased donor	11/ 25	75/ 116		0.43 (0.18, 1.03)			
	Male	12/ 25	67/ 116		0.68 (0.28, 1.61)			
Pedersen 2021[56]	CNI	18/ 18	36/ 40		4.56 (0.23, 89.34)			
	Deceased donor	6/ 18	23/ 40		0.37 (0.12, 1.18)			
	Male	9/ 18	15/ 40		1.67 (0.54, 5.13)			
	mTOR	0/ 18	1/ 40		0.71 (0.03, 18.32)			
Peled 2021[57]	Antimetabolite	5/ 14	53/ 63		0.11 (0.03, 0.38)			Mycophenolic acid therapy, age, sex, everolimus therapy, lymphocyte absolute, duration from heart transplant to antibody testing
	Male	9/ 14	41/ 63		0.97 (0.29, 3.24)			
	mTOR	9/ 14	11/ 63	OR	8.51 (2.39, 30.36)		4.3 (0.66-30.76) [b, c]	
Prendecki 2021 [59]	Male			OR	-	1.04 (0.68-1.59)		
Rabinowich 2021[61]	Antimetabolite	18/ 38	26/ 42	OR	0.55 (0.23, 1.35)		0.56 (0.29-0.87)[b, c]	Age, eGFR, high dose steroid during the last 12 months, triple immunosuppression, low dose prednisone, MMF
	CNI	35/ 39	40/ 42		0.44 (0.08, 2.54)			
	Male	28/ 38	28/ 42		1.4 (0.53, 3.68)			
	mTOR	11/ 38	9/ 42		1.49 (0.54, 4.13)			
Rashidi-Alavijeh 2021[62]	Antimetabolite	5/ 34	6/ 9		0.09 (0.02, 0.46)			
	CNI	31/ 34	9/ 9		0.47 (0.02, 10.01)			
	Male	20/ 34	6/ 9		0.71 (0.15, 3.35)			
	mTOR	20/ 34	3/ 9		2.86 (0.61, 13.4)			
Rozen-Zvi 2021[65]	Antimetabolite	66/ 112	160/ 196		0.32 (0.19, 0.54)			
	ATG	3/ 112	11/ 196	OR	0.46 (0.13, 1.7)	0.463 (0.126-1.696)		
	Deceased donor	21/ 112	53/ 196		0.62 (0.35, 1.1)			
	Male	76/ 112	121/ 196		1.31 (0.8, 2.14)			
	mTOR	10/ 112	16/ 196		1.1 (0.48, 2.52)			
	Rituximab	0/ 112	6/ 196		0.13 (0.01, 2.33)			
Russo 2021[67]	Antimetabolite	24/ 43	33/ 39	OR	0.23 (0.08, 0.66)		0.19 (0.06-0.59)[b]	Age
	Male	24/ 43	23/ 39		0.88 (0.37, 2.11)			
	mTOR	6/ 43	4/ 39		1.42 (0.37, 5.46)			
Shostak 2021[74]	Antimetabolite	25/ 31	28/ 137	OR	0.26 (0.08, 0.81)	0.25 (0.08-0.8)		
	Male	21/ 31	91/ 137	OR	1.06 (0.46, 2.44)	0.94 (0.41-2.16)		
	mTOR	1/ 31	28/ 137	OR	0.13 (0.02, 0.99)	0.13 (0.02-0.99)		
			mRNA-1273 vaccine					
Benotmane 2021[5]	Antimetabolite	76/ 98	91/ 106		0.57 (0.28, 1.17)			
	CNI	89/ 98	84/ 106		2.59 (1.13, 5.95)			
	Deceased donor	79/ 98	84/ 106		1.09 (0.55, 2.16)			
	Male	64/ 98	66/ 106		1.14 (0.64, 2.02)			
	mTOR	18/ 98	9/ 106		2.43 (1.03, 5.69)			
Crespo 2021[17]	Antimetabolite			OR	-	0.68 (0.28-1.61)[b]		
	CNI			OR	-	0.39 (0.04-3.7)[b]		
	Male			OR	-	0.53 (0.22-1.27)[b]		
Cucchiari 2021[18]	ATG			OR	-	0.14 (0.02-1.14)[b]	0.17 (0.02-1.59)[b]	Age, baseline immunosuppression, ATG, eGFR
	mTOR			OR	-	2.86 (1.12-7.69)[b]	3.57 (1.12-11.11)[b]	
Sanders 2021 [69]	Antimetabolite	116/164	115/124		0.19 (0.09, 0.4)			
	CNI	127/164	109/124		0.47 (0.25, 0.91)			
	Deceased donor	56/164	51/124		0.74 (0.46, 1.2)			
	Male	96/164	64/124		1.32 (0.83, 2.12)			
	mTORi	14/164	3/124		3.76 (1.06, 13.4)			
	Rituximab	1/164	1/124	OR	0.76 (0.05, 12.18)	3.82 (1.07-13.6)		

© 2022 Manothummetha K et al. *JAMA Network Open.*

			BNT162b2 vaccine or mRNA-1273 vaccine					
D'Offizi 2021[19]	Antimetabolite	17/47	13/14		0.04 (0.01, 0.36)			
Debska-Slizien 2021 [22]	Antimetabolite<br>Deceased donor<br>Male	52/73<br>69/73<br>44/73	60/69<br>64/69<br>39/69		0.37 (0.16, 0.88)<br>1.35 (0.35, 5.24)<br>1.17 (0.6, 2.28)			
Kantauskaite 2021[41]	Antimetabolite<br>CNI<br>Male<br>mTOR	28/ 56<br>54/ 56<br>35/ 56<br>4/ 56	163/ 169<br>163/ 169<br>113/ 169<br>3/ 169	OR	0.04 (0.01, 0.1)<br>0.99 (0.2, 5.07)<br>0.83 (0.44, 1.55)<br>4.26 (0.92, 19.64)		1.48 (0.64-3.41)	Age, gender, eGFR, time after transplantation, no use of MMF, no use of CNI, no use of steroids, dual therapy
Villanego 2021[78]	Antimetabolite<br>ATG<br>CNI<br>Male<br>mTOR<br>Rituximab	45/ 57<br>2/ 57<br>51/ 57<br>42/ 57<br>8/ 57<br>0/ 57	33/ 34<br>3/ 34<br>33/ 34<br>19/ 34<br>2/ 34<br>1/ 34	OR	0.11 (0.01, 0.92)<br>0.38 (0.06, 2.37)<br>0.26 (0.03, 2.24)<br>2.21 (0.9, 5.42)<br>2.61 (0.52, 13.1)<br>0.19 (0.01, 4.9)		0.08 (0.01-0.53)	Age, time from kidney transplant to COVID-19 vaccine, serum creatinine, MPA

ATG: Anti-thymocyte globulin; CNI: Calcineurin inhibitor;; eGFR: estimated glomerular filtration rate; MDRD- Modification of Diet in Renal Disease; MMF: Mycophenolate mofetil; MPA: Mycophenolic acid; mTOR: mammalian (mechanistic) target of rapamycin; OR: odd ratio; RR: relative risk; CI: confidence interval

a: Multivariable ORs in each primary study were derived from the same regression model

b: Odd ratio of non-response of humoral immunity was transformed to odd ratio of response of humoral immunity by log transformation

c: These values were not used to calculate the pooled odds ratios in meta-analysis due to significant symmetry issues.

© 2022 Manothummetha K et al. *JAMA Network Open.*

eTable 7. Continuous Data of Potential Risk Factors for Diminished Humoral Immune Response

Study	Number of patients for analysis	Age (years)	Duration after transplantation (years)	BMI (kg/m^2)	Lymphocyte count (x10^3/mm^3)
Alejo 2021[1]	18	Median (IQR)<br>58 (50-65)	Median (IQR)<br>7.1 (2.3-16.2)	-	-
Azzi 2021 [2]	76	Median (IQR)<br>Responder<br>58 (41-70)<br>Non-responder<br>63 (54-69)	Median (range)<br>4 (0.25-22)	Median (IQR)<br>Responder<br>30.3 (25.5-34.3)<br>Non-responder<br>29.5 (23.7-32.4)	-
Ben Zadok 2021[3]	37	Median (IQR)<br>Responder<br>46 (34-63)<br>Non-responder<br>68 (59-70)	Median (IQR)<br>Responder<br>7 (2.7-14.5)<br>Non-responder<br>9.9 (1.1-13.5)	-	-
Benotmane 2021<br>(1 dose Moderna) [4]	241	Median (IQR)<br>Responder<br>58.4 (43.3-66.9)<br>Non-responder<br>57.7 (49.6-67.7)	Median (IQR)<br>Responder<br>15.4 (8.6-25.9)<br>Non-responder<br>5.8 (2.8-11.9)	Median (IQR)<br>Responder<br>26.4 (21.9-29.9)<br>Non-responder<br>25.7 (22.8-29.4)	-
Benotmane 2021<br>(2 doses Moderna) [5]	204	Median (IQR)<br>Responder<br>57.3 (46.9-66.2)<br>Non-responder<br>58 (51-67.7)	Median (IQR)<br>Responder<br>7.1 (3.8-14.7)<br>Non-responder<br>5.4 (2.4 -12)	Median (IQR)<br>Responder<br>25.9 (22.6-29.9)<br>Non-responder<br>25.4 (22.3-27.6)	-
Benotmane 2021<br>(3 doses)[6]	159	Median (IQR)<br>57.6 (49.6-66.1)	Median (IQR)<br>5.3 (1.9-11.1)	-	-
Bertrand 2021 [8]	45	Mean (SD)<br>63.5 (16.3)	Median<br>6.9 (range 0.2-30.2)	Mean (SD)<br>26.2 (4.7)	Mean (SD)<br>0.447 (0.263)
Bertrand 2021[9]	235	Mean (SD)<br>60.8 (15.1)	-	-	-
Bertrand 2021 (3 doses) [10]	80	Mean (SD)<br>63.6 (15.7)	Median (IQR)<br>7.3 (3.4-14.1)	-	-
Boyarsky 2021<br>(1 dose of mRNA vaccines) [11]	436	Median (IQR)<br>55.9 (41.3-67.4)	Median (IQR)<br>6.2 (2.7-12.7)	-	-
Cao 2021[14]	37	Median (IQR)<br>64 (50-69)	-	-	-
Chavarot 2021 [15]	101	Median (IQR)<br>64 (53-73)	Median (IQR)<br>4.9 (2.4-8.7)	-	-
Chavarot 2021 (3 doses)[16]	62	Median (IQR)<br>Responder<br>51.5 (42-58)	-	-	-

© 2022 Manothummetha K et al. *JAMA Network Open.*

		Non-responder<br>64 (51.5-72)			
Cholankeril 2021 [83]	69	Median (IQR)<br>Responder<br>62 (50-66)<br>Non-responder<br>64 (52-69)	Median (IQR)<br>Responder<br>5 (3.2-8.6)<br>Non-responder<br>2.2 (0.9-4.7)	-	-
Crespo 2021 [17]	90	Mean (SD)<br>59.7 (12.5)	-	Mean (SD)<br>27.7 (5.9)	-
Cucchiari 2021[18]	148	Mean (SD)<br>57.6 (14.3)	Median (IQR)<br>1.7 (0.8-4.9)	Mean (SD)<br>25.6 (4.2)	-
D'Offizi 2021 [19]	61	Median (IQR)<br>59 (56-61)	Median (IQR)<br>6 (3-10)	-	-
Danthu 2021 [20]	74	Mean (SD)<br>64.8 (11.5)	Mean (SD)<br>6.4 (7.8)	Mean (SD)<br>26.7 (5.8)	Mean (SD)<br>1.630 (1250)
Davidov 2021[21]	76	Median (IQR)<br>Responder<br>60 (46-69)<br>Non-responder<br>68 (61-71)	Median (IQR)<br>Responder<br>12 (4-17)<br>Non-responder<br>8 (3-11)	Median (IQR)<br>Responder<br>25 (22-28)<br>Non-responder<br>25 (23-28)	-
Debska-Slizien 2021 [22]	142	Median (IQR)<br>Responder<br>48 (40-61)<br>Non-responder<br>58 (50-66)	Median (IQR)<br>Responder<br>10 (6-19)<br>Non-responder<br>7 (2.5-12)	Median (IQR)<br>Responder<br>25.0 (23.1-28.4)<br>Non-responder<br>25.4 (22.5-28.4)	-
Del Bello 2021[23]	396	Mean (SD)<br>59 (15)	-	-	-
Ducloux 2021[24]	153	Median (IQR)<br>Responder<br>63 (54-72)<br>Non-responder<br>64 (57-71)	Median (IQR)<br>Responder<br>13.17 (5-16.17)<br>Non-responder<br>2.3 (1.3-7)	-	-
Firket 2021[25]	10	Mean (SD)<br>49.7(13.8)	-	Mean (SD)<br>26.45 (3.84)	-
Georgery 2021<br>(1st dose)[26]	78	Median (range)<br>62 (18-84)	Median (range)<br>9.7 (0.33-50.7)	-	-
Georgery 2021<br>(2nd dose)[27]	79	Median (range)<br>61 (18-88)	Median (range)<br>8.8 (0.3-50.8)	-	-
Grupper 2021[28]	136	Mean (SD)<br>Responder<br>54.6 (12.8)<br>Non-responder<br>60.9 (12.2)	Mean (SD)<br>Responder<br>5.7 (5.8)<br>Non-responder<br>4.1 (4.6)	Mean (SD)<br>Responder<br>27.4 (3.9)<br>Non-responder<br>26.7 (4.2)	Mean (SD)<br>Responder<br>2.27 (0.8)<br>Non-responder<br>1.83 (0.9)

© 2022 Manothummetha K et al. *JAMA Network Open.*

Guarino 2021[29]	365	Mean (SD)<br>Responder<br>61.7 (13.9)<br>Non-responder<br>65 (9.3)	Mean (SD)<br>Responder<br>14.8 (8.8)<br>Non-responder<br>11.9 (8.7)	Mean (SD)<br>Responder<br>26.8 (4.59)<br>Non-responder<br>27.7 (7.09)	-
Hall 2021[30]	127	Median (IQR)<br>Responder<br>67.8 (54.3-71.8)<br>Non-responder<br>65.9 (63.4-70.1)<br>[responder vs non-responder after 2 doses]	Median (IQR)<br>Responder<br>2.63 (1.4-6.9)<br>Non-responder<br>2.83 (1.5-5.8)<br>[responder vs non-responder after 2 doses]	-	-
Hall 2021 (3 doses)[31]	60	Median (IQR)<br>66.9 (64.0-71.8)	Median (IQR)<br>3.57 (2-6.8)	-	Median (IQR)<br>1.15 (0.90-1.60)
Haskin 2021[32]	38	Mean (SD)<br>Responder<br>19.1 (2.9)<br>Non-responder<br>17.9 (2.6)	Mean (SD)<br>Responder<br>8.2 (5.8)<br>Non-responder<br>5.9 (5.2)	-	-
Havlin 2021[33]	48	Mean (SD)<br>52.1 (14.3)	Median (range)<br>4.25 (0.3-20.1)	-	-
Havlin 2021 3rd dose [34]		Median (IQR)<br>56.2 (54-60)	Median (IQR)<br>3.5 (2.4-6.8)	-	-
Herrera 2021[35]	46 (Heart)	Median (IQR)<br>60 (20-80)	Median (IQR)<br>6.3 (0.4-21)	Median (IQR)<br>24.8 (18-36.5)	-
	58 (Liver)	61.5 (18-88)	4.6 (0.3-26.8)	26.3 (17-42)	-
Hoffman 2021[37]	15	-	Median (IQR)<br>5.35 (1.87-9.71)	-	-
Hall 2021<br>(3 doses)[31]	120	Mean (SD)<br>Responder<br>58.17 (12.7)<br>Non-responder<br>60.6 (13.2)	Mean (SD)<br>Responder<br>5.5 (6.0)<br>Non-responder<br>6.0 (6.5)	Mean (SD)<br>Responder<br>28.5 (4.84)<br>Non-responder<br>27.13 (5.04)	Mean (SD)<br>Responder<br>1.86 (0.51)<br>Non-responder<br>2.14 (1.76)
Holden 2021[38]	80	Mean (IQR)<br>Responder<br>52.9 (44.9-61.5)<br>Non-responder<br>60.2 (51.7-68.6)	Mean (IQR)<br>Responder<br>9.55 (4.8-16)<br>Non-responder<br>6.7 (3.15-14)	Mean (IQR)<br>Responder<br>27.6 (23.5-31.5)<br>Non-responder<br>26 (22.5-28.3)	-
Kamar 2021[39]	101	Mean (SD)<br>58 (2)	-	-	-
Kamar 2021<br>(4th dose) [40]	37	Mean (SEM)<br>Responder<br>60 (14)	Mean (SD)<br>Responder<br>13.4 (8.1)	-	Mean (SD)<br>Responder<br>1.4 (0.9)

© 2022 Manothummetha K et al. *JAMA Network Open.*

		Non-responder<br>58 (16)	Non-responder<br>6.6 (5.5)		Non-responder<br>0.9 (0.4)
Kantauskaite 2021[41]	225	Median (IQR)<br>62 (54-70)<br>Responder<br>61 (57-68)<br>Non-responder<br>62 (53-70)	Median (IQR)<br>6.8 (2.6-12.3)<br>Responder<br>12.5 (7.8-18.9)<br>Non-responder<br>4.7 (2.2-10.3)	-	-
Korth 2021[42]	23	Mean (SD) Responder<br>57.0 (8.1)<br>Non-responder<br>57.9 (14.9)	Mean (SD) Responder<br>17.6 (7.7)<br>Non-responder<br>9.7 (9.1)	-	-
Kumar 2021<br>(3rd dose) [43]	60	Median (IQR)<br>66.9 (64-71.8)	Median (IQR)<br>3.6 (2-6.8)	-	Median (IQR)<br>1.2 (0.9-1.6)
Marinaki 2021[44]	34	Median (IQR)<br>60 (49.1-68.4)	Median (IQR)<br>11.1 (7.3-15.8)	Mean (SD)<br>Responder<br>25.4 (5)<br>Non-responder<br>25.5 (4.8)<br>[Responder was defined by positive Ab test after the second dose]	-
Marion 2021[45]	576	Mean (SE) of patients with Ab test after the second dose<br>59 (1)	-	Median (IQR)<br>Responder<br>58.8 (22.3-28.2)<br>Non-responder<br>24.8 (22.6-28.5)	-
Massa 2021<br>(3rd dose)[46]	61	Median (IQR)<br>58 (47.1-66.1)	Median (IQR)<br>4.5 (1.8-11.3)	-	Median (IQR)<br>1.3 (0.8-1.7)
Masset 2021[47]	456	Mean (SD)<br>Responder<br>60.2 (13)<br>Non-responder<br>62.7 (11.1)<br>[Responder was defined by positive Ab test after the second dose]	Mean (SD) Responder<br>12.4 (8.9)<br>Non-responder<br>8.5 (7.6)<br>[Responder was defined by positive Ab test after the second dose]	-	-
Masset 2021<br>(3rd dose) [48]	28<br>(AZD1222- AZD1222 -mRNA vaccine or AZD1222- mRNA-mRNA vaccine)	Mean(SD)<br>58.7 (13.3)	Mean(SD)<br>8.2 (6.2)	-	Mean(SD)<br>1.8 (0.8)
	56<br>3 mRNA vaccine	Mean(SD)<br>58.7 (12)	Mean(SD)<br>8.5 (7.4)	-	Mean(SD)<br>1.7 (1)

© 2022 Manothummetha K et al. *JAMA Network Open.*

Mazzola 2021[49]	143	Median (IQR)<br>61 (55-67)	Median (IQR)<br>3.75 (1.83-8.83)	Mean (SD)<br>Responder<br>25.6 (3.8)<br>Non-responder<br>25.1 (4.4)	-
Medina-Pestana 2021[50]	942	Median (IQR)<br>50 (43-56)	Median (IQR)<br>6 (3-11)	-	-
Midtvedth 2021[52]	141	Mean (SD)<br>Responder<br>59.1 (19.7)<br>Non-responder<br>69.1 (16.2)	Mean (SD)<br>Responder<br>17.1 (13.5)<br>Non-responder<br>10.4 (8.4)	-	-
Miele 2021[53]	16	Mean (SD)<br>57 (15.9)	Median (SD)<br>9 (7.5)	-	-
Narasimhan 2021[54]	73	Median (IQR)<br>65 (53.5-69.5)	Median (IQR)<br>3.33 (1.6-5.3)	-	-
Noble 2021[55]	57	Mean (SD)<br>62 (13)	-	-	-
Pedersen 2021[56]	58	Median (IQR)<br>Responder<br>48.0 (42.3-61.4)<br>Non-responder<br>60.9 (53-67.4)	Median (IQR)<br>Responder<br>9.40 (4.5-12.9)<br>Non-responder<br>5.65 (2.3-14.1)	Median (IQR)<br>Responder<br>28.25 (25.1-31.6)<br>Non-responder<br>26.10 (22.4-29.6)	-
Peled 2021[57]	77	Median (IQR)<br>Responder<br>61.5 (47.8-68)<br>Non-responder<br>62.0 (49.5-68.5)	-	Mean (SD)<br>Responder<br>28.4 (6.6)<br>Non-responder<br>26.1 (5.1)	Mean (SD)<br>Responder<br>1.5 (0.6)<br>Non-responder<br>1.4 (0.7)
Peled 2021 (3 doses)[58]	96	Median (IQR)<br>Responder<br>58 (47-68)<br>Non-responder<br>65 (58.8-70.3)	Median (IQR)<br>6.3 (3.5-13.6)	Mean (SD)<br>Responder<br>26.9 (4.0)<br>Non-responder<br>26.8 (5.9)	-
Prendecki 2021[59]	768	-	-	Mean (SD)<br>Responder<br>26.8 (5.6)<br>Non-responder<br>25.83 (3.9)	Mean (SD)<br>Responder<br>1.75 (0.7)<br>Non-responder<br>1.59 (0.8)
Rabinowich 2021[61]	80	Mean (SD)<br>Responder<br>57.8 (11.9)<br>Non-responder<br>63.2 (11.9)	Mean (SD)<br>Responder<br>7.43 (5.53)<br>Non-responder<br>5.98 (5.9)	-	-

© 2022 Manothummetha K et al. *JAMA Network Open.*

Rashidi-Alavijeh 2021[62]	43	Median (IQR)<br>Responder<br>56.5 (46-64)<br>Non-responder<br>59 (53.5-64.5)	Median (IQR)<br>Responder<br>6.5 (3.7-12)<br>Non-responder<br>12 (5.5-14.5)	-	-
Rahav 2021 [63]	80<br>(Heart)	Median (IQR)<br>61.5 (50.0-68.0)	Median (IQR)<br>7.4 (3.3-15.1)	-	-
	111<br>(Kidney)	Median (IQR)<br>60 (49.0-70.0)	Median (IQR)<br>3.1 (1.0-9.2)	-	-
	36<br>(Liver)	Median (IQR)<br>68 (51.0-71.0)	Median (IQR)<br>7 (4.0-16.0)	-	-
Rincon-Arevalo 2021[64]	40	Median (IQR)<br>62.4 (51.3-69.5)	Median (IQR)<br>5.0 (2.0-10.0)	Mean (SD)<br>Responder<br>26.84 (4.38)<br>Non-responder<br>26.94 (4.77)	-
Rozen-Zvi 2021[65]	308	Mean (SD)<br>Responder<br>53.7 (14.5)<br>Non-responder<br>59.7 (13)	Mean (SD)<br>Responder<br>7.1 (7.2)<br>Non-responder<br>7.1 (7.8)	Mean (SD)<br>Responder<br>26.8 (5.6)<br>Non-responder<br>25.83 (3.9)	Mean (SD)<br>Responder<br>1.75 (0.7)<br>Non-responder<br>1.59 (0.8)
Ruether 2021[66]	138	Mean (SD)<br>55 (13.2)	Median (IQR)<br>7 (2-17)	Median (IQR)<br>24.8 (22.4-28.5)	Median (IQR)<br>950 (667-1,404)
Russo 2021[67]	82	Median (IQR)<br>58.5 (50.3-65)	Median (IQR)<br>5.75 (2.9-11.9)	Median (IQR)<br>Responder 26.8 (23.0-28.8)<br>Non-responder<br>25.2 (22.9-27.8)	-
Sadioğlu 2021[68]	85	Mean (SD)<br>Responder<br>38 (12)<br>Non-responder<br>48 (11)	Mean (SD)<br>Responder<br>5.8 (3.5)<br>Non-responder<br>7.2 (6)	-	-
Sanders 2021 [69]	288	Mean (SD)<br>Responder<br>53.5 (14)<br>Non-responder<br>59.5 (13.2)	-	Mean (SD)<br>Responder<br>27 (4.6)<br>Non-responder<br>26.8 (4.7)	Median (IQR)<br>Responder<br>1.3 (1-1.9)<br>Non-responder<br>1.1 (0.8-1.7)
Sattler 2021[70]	39	Mean (SD)<br>57.4 (14)	Mean (SD)<br>8.2 (6.1)	-	-
Schmidt 2021[71]	40	Mean (SD)<br>54.5 (12.7)	Median<br>6.5	-	Median<br>1.43
Schramm 2021[72]	50	Mean (SD)<br>55 (10)	Median (IQR)<br>1.89 (1.4-2.4)	-	-

© 2022 Manothummetha K et al. *JAMA Network Open.*

Schrezenmeier 2021 (3rd dose) [73]	25	Mean (SD)<br>59.7 (13.8)	-	-	-
Shostak 2021[74]	168	Median (IQR)<br>Responder<br>57 (36-63)<br>Non-responder<br>61 (51-68.5)	-	-	-
Stumpf 2021 (Subanalysis of DIAVAC)[75]	71	Overall<br>Mean (SD)<br>57 (14.4)	Overall<br>Mean (SD)<br>7.5 (6)	-	-
Stumpf 2021[76]	368	Mean (SD)<br>57.3 (13.7)	Mean (SD)<br>9.9 (6.8)	Mean (SD)<br>26.4 (4.8)	-
Thuluvath 2021 [77]	62	Mean (SD)<br>65.7 (8.7)	-	Mean (SD)<br>29.1 (5.7)	-
Villanego 2021[78]	91	Median (IQR)<br>Responder<br>59 (50–64.5)<br>Non-responder<br>62.5 (58–70.5)	Median (IQR)<br>Responder<br>8 (3.4–15.6)<br>Non-responder<br>2.3 (1.5–9.2)	-	-
Werbel 2021[79]	30	Median (IQR)<br>57 (44-62)	Median (IQR)<br>4.5 (2.3-10.5)	-	-
Wijtvliet 2021 [81]	94<br>BNT162b2<br>(1st dose)	Median (IQR)<br>56.5 (46.0-66.8)	-	-	-
	44<br>mRNA-1273<br>(1st dose)	Median (IQR)<br>54 (48.8-61.0)	-	-	-
	91<br>BNT162b2<br>(2nd dose)	Median (IQR)<br>57 (56.5-66.5)	-	-	-
	42<br>mRNA-1273<br>(2nd dose)	Median (IQR)<br>55.5 (50.0-61.0)	-	-	-
Yi 2021[82]	145	-	Median (IQR)<br>5 (3-10)	-	-

IQR: interquartile range; SD: standard deviation ; BMI: body mass index; SE: standard error

© 2022 Manothummetha K et al. *JAMA Network Open.*

eFigure 1. Forest Plots of Studied Risk Factors

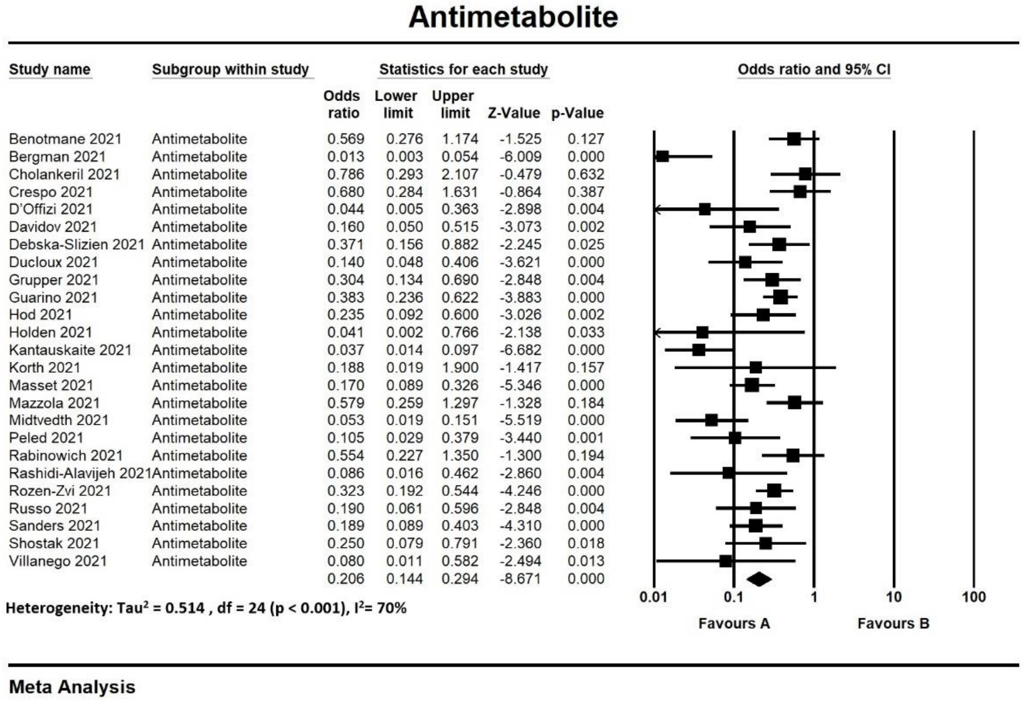

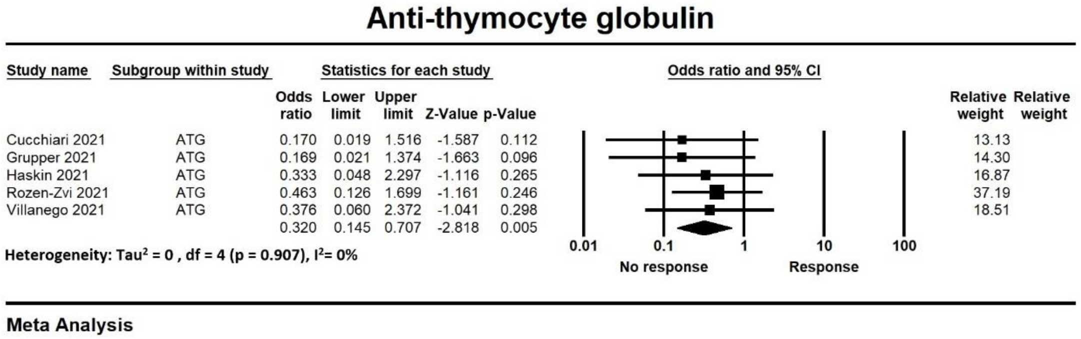

CI: confidence interval; ATG: anti-thymocyte globulin

© 2022 Manothummetha K et al. *JAMA Network Open.*

Calcineurin inhibitor

Study name	Subgroup within study	Odds ratio	Lower limit	Upper limit	Z-Value	p-Value
Benotmane 2021	CNI	2.590	1.128	5.945	2.245	0.025
Cholankeril 2021	CNI	0.207	0.022	1.957	-1.374	0.169
Crespo 2021	CNI	0.390	0.041	3.751	-0.815	0.415
Ducloux 2021	CNI	1.240	0.241	6.393	0.257	0.797
Grupper 2021	CNI	0.956	0.295	3.097	-0.075	0.940
Guarino 2021	CNI	1.371	0.761	2.468	1.051	0.293
Hod 2021	CNI	1.083	0.257	4.571	0.109	0.913
Holden 2021	CNI	6.600	0.352	123.858	1.261	0.207
Kantauskaite 2021	CNI	0.994	0.195	5.071	-0.007	0.994
Korth 2021	CNI	1.143	0.098	13.336	0.107	0.915
Masset 2021	CNI	0.650	0.304	1.389	-1.112	0.266
Mazzola 2021	CNI	0.965	0.365	2.555	-0.071	0.943
Pedersen 2021	CNI	4.562	0.233	89.343	1.000	0.317
Rabinowich 2021	CNI	0.438	0.075	2.535	-0.922	0.356
Rashidi-Alavijeh 2021	CNI	0.474	0.022	10.009	-0.480	0.631
Sanders 2021	CNI	0.460	0.239	0.886	-2.323	0.020
Villanego 2021	CNI	0.258	0.030	2.238	-1.230	0.219
		0.920	0.650	1.304	-0.467	0.641

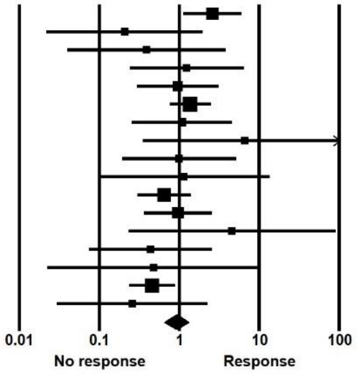

Heterogeneity: Tau^2 = 0.101 , df = 16 (p = 0.205), I^2= 21%

Meta Analysis

Deceased donor

Study name	Subgroup within study	Odds ratio	Lower limit	Upper limit	Z-Value	p-Value
Benotmane 2021	Deceased donor	1.089	0.548	2.163	0.243	0.808
Debska-Slizien 2021	Deceased donor	1.348	0.347	5.241	0.431	0.667
Grupper 2021	Deceased donor	0.468	0.221	0.990	-1.987	0.047
Haskin 2021	Deceased donor	1.286	0.330	5.017	0.362	0.718
Hod 2021	Deceased donor	0.351	0.110	1.116	-1.774	0.076
Masset 2021	Deceased donor	0.645	0.395	1.051	-1.759	0.079
Midtvedth 2021	Deceased donor	0.430	0.179	1.032	-1.889	0.059
Pedersen 2021	Deceased donor	0.370	0.115	1.183	-1.677	0.094
Rozen-Zvi 2021	Deceased donor	0.623	0.352	1.101	-1.630	0.103
Sanders 2021	Deceased donor	0.742	0.458	1.202	-1.213	0.225
		0.659	0.525	0.827	-3.597	0.000

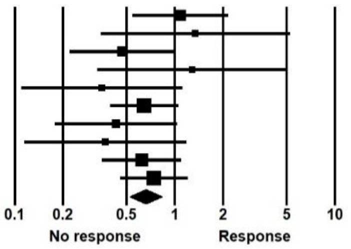

Heterogeneity: Tau^2 = 0 , df = 9 (p = 0.521), I^2= 0%

Meta Analysis

CI: confidence interval;

© 2022 Manothummetha K et al. *JAMA Network Open.*

Male

Study name	Subgroup within study	Odds ratio	Lower limit	Upper limit	Z-Value	p-Value
Benotmane 2021	Male	1.141	0.644	2.021	0.451	0.652
Cholankeril 2021	Male	1.766	0.620	5.033	1.065	0.287
Crespo 2021	Male	0.530	0.221	1.273	-1.420	0.156
Davidov 2021	Male	1.650	0.600	4.540	0.970	0.332
Debska-Slizien 2021	Male	1.167	0.598	2.276	0.453	0.650
Ducloux 2021	Male	1.400	0.709	2.765	0.969	0.333
Grupper 2021	Male	1.208	0.582	2.503	0.507	0.612
Guarino 2021	Male	0.800	0.450	1.422	-0.759	0.448
Haskin 2021	Male	3.000	0.742	12.127	1.541	0.123
Hod 2021	Male	0.700	0.280	1.749	-0.763	0.445
Holden 2021	Male	0.733	0.291	1.845	-0.659	0.510
Kantauskaite 2021	Male	1.480	0.641	3.416	0.919	0.358
Korth 2021	Male	0.667	0.089	4.994	-0.395	0.693
Masset 2021	Male	1.347	0.924	1.962	1.550	0.121
Mazzola 2021	Male	1.356	0.586	3.141	0.711	0.477
Midtvedth 2021	Male	0.675	0.284	1.606	-0.888	0.374
Pedersen 2021	Male	1.667	0.542	5.129	0.891	0.373
Peled 2021	Male	0.966	0.288	3.238	-0.056	0.955
Prendecki 2021	Male	1.040	0.680	1.590	0.181	0.856
Rabinowich 2021	Male	1.400	0.533	3.678	0.683	0.495
Rashidi-Alavijeh 2021	Male	0.714	0.152	3.349	-0.427	0.670
Rozen-Zvi 2021	Male	1.309	0.802	2.136	1.075	0.282
Russo 2021	Male	0.879	0.366	2.112	-0.289	0.773
Sanders 2021	Male	1.324	0.828	2.117	1.170	0.242
Shostak 2021	Male	0.940	0.410	2.158	-0.146	0.884
Villanego 2021	Male	2.211	0.901	5.424	1.732	0.083
		1.158	1.007	1.332	2.061	0.039

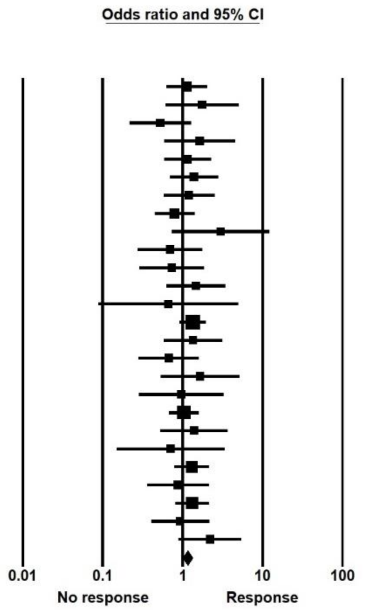

Heterogeneity: Tau^2 = 0 , df = 25 (p = 0.874), I^2= 0%

Meta Analysis

mTOR inhibitor

Study name	Subgroup within study	Odds ratio	Lower limit	Upper limit	Z-Value	p-Value
Benotmane 2021	mTORi	2.425	1.033	5.692	2.035	0.042
Cucchiari 2021	mTORi	3.570	1.133	11.244	2.174	0.030
Davidov 2021	mTORi	0.392	0.105	1.458	-1.397	0.162
Ducloux 2021	mTORi	1.710	0.319	9.154	0.627	0.531
Grupper 2021	mTORi	1.739	0.478	6.328	0.840	0.401
Guarino 2021	mTORi	0.521	0.308	0.883	-2.423	0.015
Hod 2021	mTORi	1.423	0.303	6.680	0.447	0.655
Holden 2021	mTORi	1.889	0.114	31.399	0.443	0.657
Kantauskaite 2021	mTORi	4.256	0.923	19.637	1.857	0.063
Korth 2021	mTORi	2.333	0.284	19.172	0.788	0.430
Masset 2021	mTORi	1.370	0.620	3.029	0.778	0.437
Mazzola 2021	mTORi	1.141	0.448	2.902	0.276	0.782
Pedersen 2021	mTORi	0.712	0.028	18.317	-0.205	0.837
Peled 2021	mTORi	4.300	0.630	29.356	1.488	0.137
Rabinowich 2021	mTORi	1.494	0.540	4.131	0.773	0.439
Rashidi-Alavijeh 2021	mTORi	2.857	0.609	13.395	1.332	0.183
Rozen-Zvi 2021	mTORi	1.103	0.483	2.521	0.232	0.816
Russo 2021	mTORi	1.419	0.369	5.457	0.509	0.611
Sanders 2021	mTORi	3.820	1.071	13.619	2.066	0.039
Shostak 2021	mTORi	0.130	0.018	0.915	-2.050	0.040
Villanego 2021	mTORi	2.612	0.521	13.098	1.167	0.243
		1.456	1.018	2.083	2.055	0.040

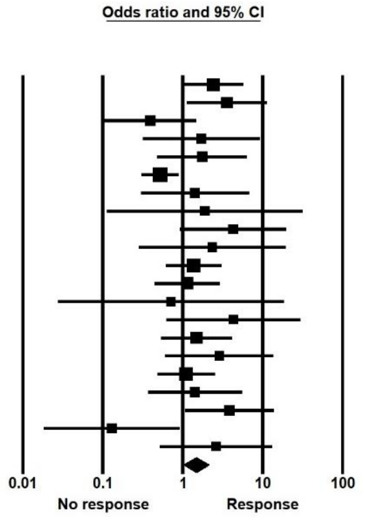

Heterogeneity: Tau^2 = 0.256 , df = 20 (p = 0.023), I^2= 42%

Meta Analysis

CI: confidence interval; mTORi: mTOR inhibitor

© 2022 Manothummetha K et al. *JAMA Network Open.*

Rituximab

Study name	Subgroup within study	Odds ratio	Lower limit	Upper limit	Z-Value	p-Value
Grupper 2021	Rituximab	0.547	0.055	5.400	-0.517	0.605
Haskin 2021	Rituximab	0.080	0.013	0.486	-2.743	0.006
Rozen-Zvi 2021	Rituximab	0.130	0.007	2.334	-1.384	0.166
Sanders 2021	Rituximab	0.755	0.047	12.184	-0.198	0.843
Villanego 2021	Rituximab	0.194	0.008	4.904	-0.995	0.320
		0.207	0.070	0.614	-2.837	0.005

Heterogeneity: $Tau^2 = 0$, df = 4 ($p = 0.611$), $I^2 = 0\%$

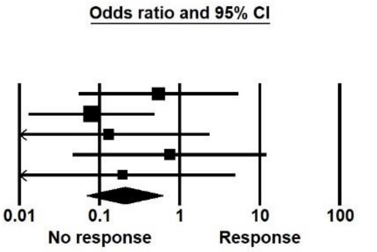

Meta Analysis

Age

Study name	Subgroup within study	Difference in means	Standard error	Variance	Lower limit	Upper limit	Z-Value	p-Value
Grupper 2021	Age	-6.300	2.201	4.845	-10.614	-1.986	-2.862	0.004
Guarino 2021	Age	-3.330	1.556	2.420	-6.379	-0.281	-2.140	0.032
Haskin 2021	Age	1.200	0.940	0.884	-0.643	3.043	1.276	0.202
Hod 2021	Age	-2.430	2.496	6.230	-7.322	2.462	-0.974	0.330
Korth 2021	Age	-0.900	7.009	49.123	-14.637	12.837	-0.128	0.898
Masset 2021	Age	-2.500	1.132	1.281	-4.718	-0.282	-2.209	0.027
Midtvedth 2021	Age	-10.000	3.717	13.815	-17.285	-2.715	-2.690	0.007
Rabinowich 2021	Age	-5.440	2.664	7.098	-10.662	-0.218	-2.042	0.041
Rozen-Zvi 2021	Age	-6.020	1.606	2.578	-9.167	-2.873	-3.749	0.000
Sanders 2021	Age	-6.000	1.626	2.643	-9.187	-2.813	-3.690	0.000
		-3.942	1.134	1.286	-6.165	-1.719	-3.475	0.001

Heterogeneity: $Tau^2 = 8.074$, df = 9 ($p < 0.001$), $I^2 = 73\%$

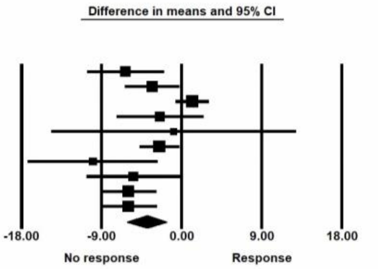

Meta Analysis

CI: confidence interval;

© 2022 Manothummetha K et al. *JAMA Network Open.*

Body Mass Index

Study name	Subgroup within study	Difference in means	Standard error	Variance	Lower limit	Upper limit	Z-Value	p-Value
Grupper 2021	BMI	0.690	0.725	0.525	-0.730	2.110	0.952	0.341
Guarino 2021	BMI	-0.930	0.642	0.413	-2.189	0.329	-1.448	0.148
Hod 2021	BMI	1.370	0.951	0.905	-0.495	3.235	1.440	0.150
Masset 2021	BMI	-0.100	0.459	0.211	-1.000	0.800	-0.218	0.828
Midtvedth 2021	BMI	0.500	0.949	0.900	-1.359	2.359	0.527	0.598
Peled 2021	BMI	2.300	1.593	2.536	-0.821	5.421	1.444	0.149
Rabinowich 2021	BMI	0.970	1.071	1.146	-1.129	3.069	0.906	0.365
Rozen-Zvi 2021	BMI	-0.100	0.549	0.301	-1.175	0.975	-0.182	0.855
Sanders 2021	BMI	0.200	0.553	0.305	-0.883	1.283	0.362	0.717
		0.145	0.230	0.053	-0.306	0.596	0.629	0.529

Statistics for each study

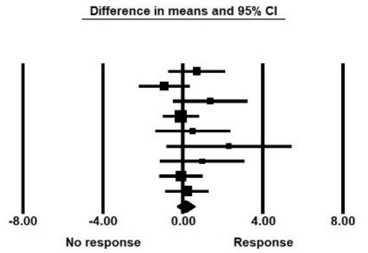

Heterogeneity: Tau^2 = 0.005 , df = 8 (p = 0.425), I^2= 1%

Meta Analysis

Lymphocyte count

Study name	Subgroup within study	Difference in means	Standard error	Variance	Lower limit	Upper limit	Z-Value	p-Value	Relative weight	Relative weight
Grupper 2021	Lymphocyte	0.440	0.153	0.023	0.140	0.740	2.875	0.004	31.29	
Hod 2021	Lymphocyte	-0.280	0.278	0.077	-0.825	0.265	-1.007	0.314	16.05	
Peled 2021	Lymphocyte	0.100	0.202	0.041	-0.296	0.496	0.495	0.621	23.95	
Rabinowich 2021	Lymphocyte	0.160	0.169	0.029	-0.171	0.491	0.948	0.343	28.71	
		0.163	0.133	0.018	-0.099	0.424	1.220	0.223		

Statistics for each study

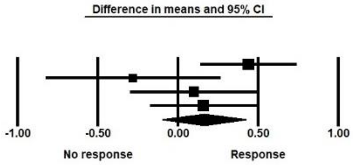

Heterogeneity: Tau^2 = 0.033 , df = 3 (p = 0.125), I^2= 48%

Meta Analysis

Duration after transplantation

Study name	Subgroup within study	Difference in means	Standard error	Variance	Lower limit	Upper limit	Z-Value	p-Value	Relative weight	Relative weight
Grupper 2021	Time from transplant	1.600	0.900	0.810	-0.164	3.364	1.778	0.075	14.62	
Guarino 2021	Time from transplant	2.850	1.056	1.115	0.781	4.919	2.700	0.007	13.44	
Haskin 2021	Time from transplant	2.300	1.880	3.535	-1.385	5.985	1.223	0.221	8.19	
Hod 2021	Time from transplant	-0.500	1.212	1.468	-2.875	1.875	-0.413	0.680	12.29	
Korth 2021	Time from transplant	7.900	4.474	20.018	-0.869	16.669	1.766	0.077	2.24	
Masset 2021	Time from transplant	3.900	0.775	0.600	2.381	5.419	5.033	0.000	15.56	
Midtvedth 2021	Time from transplant	6.700	2.090	4.368	2.604	10.796	3.206	0.001	7.22	
Rabinowich 2021	Time from transplant	1.450	1.282	1.644	-1.063	3.963	1.131	0.258	11.79	
Rozen-Zvi 2021	Time from transplant	-0.060	0.895	0.801	-1.815	1.695	-0.067	0.947	14.66	
		2.173	0.712	0.507	0.777	3.569	3.051	0.002		

Statistics for each study

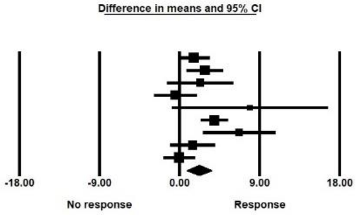

Heterogeneity: Tau^2 = 2.661 , df = 8 (p = 0.003), I^2= 66%

Meta Analysis

CI: confidence interval; BMI: body mass index

© 2022 Manothummetha K et al. *JAMA Network Open.*

eFigure 2. Sensitivity Analysis of Studied Risk Factors

Antimetabolite sensitivity

Study name	Subgroup within study	Statistics with study removed				
		Point	Lower limit	Upper limit	Z-Value	p-Value
Benotmane 2021	Antimetabolite	0.195	0.135	0.281	-8.752	0.000
Bergman 2021	Antimetabolite	0.231	0.166	0.320	-8.798	0.000
Cholankeril 2021	Antimetabolite	0.195	0.136	0.279	-8.904	0.000
Crespo 2021	Antimetabolite	0.195	0.136	0.280	-8.846	0.000
D'Offizi 2021	Antimetabolite	0.213	0.149	0.304	-8.471	0.000
Davidov 2021	Antimetabolite	0.207	0.143	0.300	-8.369	0.000
Debska-Slizien 2021	Antimetabolite	0.199	0.137	0.289	-8.490	0.000
Ducloux 2021	Antimetabolite	0.209	0.145	0.302	-8.336	0.000
Grupper 2021	Antimetabolite	0.201	0.138	0.292	-8.398	0.000
Guarino 2021	Antimetabolite	0.197	0.134	0.288	-8.356	0.000
Hod 2021	Antimetabolite	0.204	0.140	0.296	-8.366	0.000
Holden 2021	Antimetabolite	0.210	0.147	0.301	-8.523	0.000
Kantauskaite 2021	Antimetabolite	0.228	0.163	0.319	-8.658	0.000
Korth 2021	Antimetabolite	0.206	0.143	0.296	-8.529	0.000
Masset 2021	Antimetabolite	0.207	0.142	0.301	-8.207	0.000
Mazzola 2021	Antimetabolite	0.195	0.136	0.282	-8.746	0.000
Midtvedth 2021	Antimetabolite	0.221	0.155	0.313	-8.447	0.000
Peled 2021	Antimetabolite	0.211	0.147	0.304	-8.369	0.000
Rabinowich 2021	Antimetabolite	0.196	0.136	0.283	-8.701	0.000
Rashidi-Alavijeh 2021	Antimetabolite	0.211	0.147	0.303	-8.421	0.000
Rozen-Zvi 2021	Antimetabolite	0.198	0.135	0.291	-8.250	0.000
Russo 2021	Antimetabolite	0.206	0.142	0.298	-8.383	0.000
Sanders 2021	Antimetabolite	0.206	0.141	0.299	-8.262	0.000
Shostak 2021	Antimetabolite	0.204	0.141	0.295	-8.433	0.000
Villanego 2021	Antimetabolite	0.210	0.146	0.302	-8.455	0.000
		0.206	0.144	0.294	-8.671	0.000

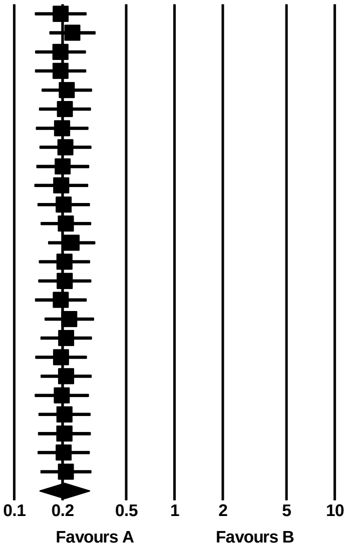

Meta Analysis

Anti-thymocyte globulin sensitivity analysis

Study name	Subgroup within study	Statistics with study removed				
		Point	Lower limit	Upper limit	Z-Value	p-Value
Cucchiari 2021	ATG	0.352	0.150	0.824	-2.406	0.016
Grupper 2021	ATG	0.356	0.151	0.838	-2.365	0.018
Haskin 2021	ATG	0.317	0.133	0.757	-2.588	0.010
Rozen-Zvi 2021	ATG	0.257	0.095	0.699	-2.662	0.008
Villanego 2021	ATG	0.308	0.128	0.742	-2.626	0.009
		0.320	0.145	0.707	-2.818	0.005

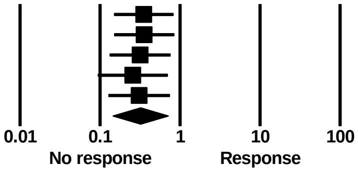

Meta Analysis

CI: confidence interval; ATG: anti-thymocyte globulin

© 2022 Manothummetha K et al. *JAMA Network Open.*

Calcineurin inhibitor sensitivity analysis

Study name	Subgroup within study	Point	Lower limit	Upper limit	Z-Value	p-Value
Benotmane 2021	CNI	0.816	0.607	1.097	-1.346	0.178
Cholankeril 2021	CNI	0.952	0.676	1.342	-0.280	0.780
Crespo 2021	CNI	0.938	0.655	1.343	-0.350	0.726
Ducloux 2021	CNI	0.907	0.627	1.312	-0.519	0.604
Grupper 2021	CNI	0.915	0.627	1.337	-0.458	0.647
Guarino 2021	CNI	0.850	0.582	1.241	-0.841	0.400
Hod 2021	CNI	0.910	0.627	1.321	-0.495	0.621
Holden 2021	CNI	0.898	0.638	1.264	-0.616	0.538
Kantauskaite 2021	CNI	0.915	0.632	1.326	-0.468	0.640
Korth 2021	CNI	0.915	0.635	1.317	-0.480	0.631
Masset 2021	CNI	0.965	0.658	1.415	-0.182	0.855
Mazzola 2021	CNI	0.913	0.621	1.342	-0.462	0.644
Pedersen 2021	CNI	0.901	0.635	1.278	-0.583	0.560
Rabinowich 2021	CNI	0.945	0.659	1.355	-0.307	0.759
Rashidi-Alavijeh 2021	CNI	0.927	0.646	1.331	-0.411	0.681
Sanders 2021	CNI	1.086	0.798	1.478	0.525	0.600
Villanego 2021	CNI	0.950	0.670	1.346	-0.290	0.771
		0.920	0.650	1.304	-0.467	0.641

Statistics with study removed

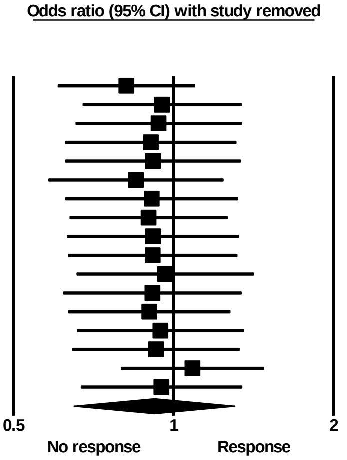

Meta Analysis

Deceased donor sensitivity analysis

Study name	Subgroup within study	Point	Lower limit	Upper limit	Z-Value	p-Value
Benotmane 2021	Deceased donor	0.619	0.487	0.788	-3.898	0.000
Debska-Slizien 2021	Deceased donor	0.645	0.512	0.813	-3.722	0.000
Grupper 2021	Deceased donor	0.682	0.537	0.866	-3.142	0.002
Haskin 2021	Deceased donor	0.646	0.513	0.814	-3.710	0.000
Hod 2021	Deceased donor	0.676	0.536	0.852	-3.313	0.001
Masset 2021	Deceased donor	0.662	0.510	0.859	-3.108	0.002
Midtvedth 2021	Deceased donor	0.679	0.537	0.860	-3.217	0.001
Pedersen 2021	Deceased donor	0.674	0.535	0.850	-3.334	0.001
Rozen-Zvi 2021	Deceased donor	0.666	0.518	0.855	-3.187	0.001
Sanders 2021	Deceased donor	0.637	0.492	0.824	-3.431	0.001
		0.659	0.525	0.827	-3.597	0.000

Statistics with study removed

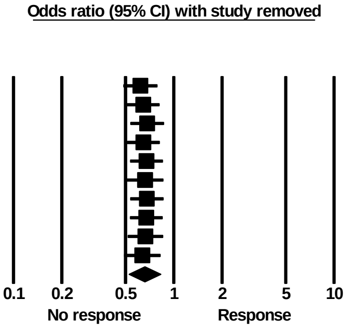

Meta Analysis

CI: confidence interval; CNI: calcineurin inhibitor

© 2022 Manothummetha K et al. *JAMA Network Open.*

Male sensitivity analysis

Study name	Subgroup within study	Point	Lower limit	Upper limit	Z-Value	p-Value
Benotmane 2021	Male	1.160	1.004	1.339	2.011	0.044
Cholankeril 2021	Male	1.150	0.998	1.324	1.936	0.053
Crespo 2021	Male	1.182	1.026	1.362	2.317	0.021
Davidov 2021	Male	1.150	0.999	1.325	1.945	0.052
Debska-Slizien 2021	Male	1.158	1.004	1.336	2.010	0.044
Ducloux 2021	Male	1.149	0.996	1.325	1.902	0.057
Grupper 2021	Male	1.157	1.003	1.334	2.000	0.045
Guarino 2021	Male	1.186	1.026	1.370	2.315	0.021
Haskin 2021	Male	1.147	0.997	1.320	1.916	0.055
Hod 2021	Male	1.172	1.018	1.351	2.203	0.028
Holden 2021	Male	1.171	1.016	1.349	2.186	0.029
Kantauskaite 2021	Male	1.150	0.998	1.326	1.934	0.053
Korth 2021	Male	1.162	1.010	1.336	2.093	0.036
Masset 2021	Male	1.131	0.973	1.315	1.599	0.110
Mazzola 2021	Male	1.153	1.001	1.329	1.970	0.049
Midtvedth 2021	Male	1.175	1.020	1.354	2.233	0.026
Pedersen 2021	Male	1.152	1.000	1.326	1.965	0.049
Peled 2021	Male	1.161	1.009	1.337	2.081	0.037
Prendecki 2021	Male	1.174	1.012	1.361	2.119	0.034
Rabinowich 2021	Male	1.154	1.002	1.329	1.983	0.047
Rashidi-Alavijeh 2021	Male	1.163	1.011	1.338	2.108	0.035
Rozen-Zvi 2021	Male	1.146	0.990	1.326	1.830	0.067
Russo 2021	Male	1.167	1.013	1.344	2.134	0.033
Sanders 2021	Male	1.143	0.988	1.324	1.793	0.073
Shostak 2021	Male	1.166	1.011	1.343	2.115	0.034
Villanego 2021	Male	1.140	0.989	1.313	1.813	0.070
		1.158	1.007	1.332	2.061	0.039

Odds ratio (95% CI) with study removed

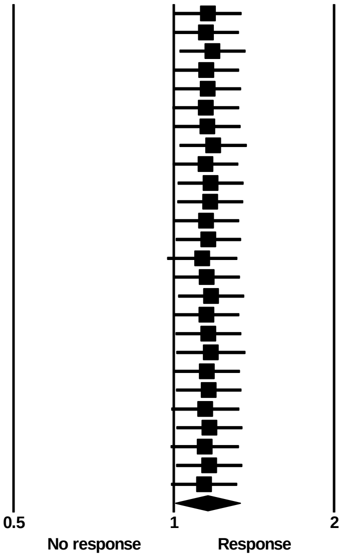

Meta Analysis

mTOR inhibitor sensitivity analysis

Study name	Subgroup within study	Point	Lower limit	Upper limit	Z-Value	p-Value
Benotmane 2021	mTORi	1.396	0.963	2.024	1.760	0.078
Cucchiari 2021	mTORi	1.373	0.959	1.966	1.732	0.083
Davidov 2021	mTORi	1.545	1.080	2.209	2.382	0.017
Ducloux 2021	mTORi	1.453	1.002	2.106	1.970	0.049
Grupper 2021	mTORi	1.448	0.995	2.108	1.935	0.053
Guarino 2021	mTORi	1.620	1.201	2.186	3.157	0.002
Hod 2021	mTORi	1.463	1.007	2.125	1.998	0.046
Holden 2021	mTORi	1.454	1.008	2.099	2.002	0.045
Kantauskaite 2021	mTORi	1.390	0.971	1.990	1.799	0.072
Korth 2021	mTORi	1.443	0.999	2.084	1.954	0.051
Masset 2021	mTORi	1.475	1.000	2.175	1.959	0.050
Mazzola 2021	mTORi	1.492	1.016	2.190	2.041	0.041
Pedersen 2021	mTORi	1.472	1.021	2.122	2.072	0.038
Peled 2021	mTORi	1.411	0.983	2.025	1.867	0.062
Rabinowich 2021	mTORi	1.461	0.998	2.140	1.951	0.051
Rashidi-Alavijeh 2021	mTORi	1.419	0.983	2.048	1.867	0.062
Rozen-Zvi 2021	mTORi	1.500	1.018	2.208	2.053	0.040
Russo 2021	mTORi	1.464	1.006	2.132	1.989	0.047
Sanders 2021	mTORi	1.378	0.963	1.973	1.754	0.079
Shostak 2021	mTORi	1.524	1.087	2.139	2.441	0.015
Villanego 2021	mTORi	1.427	0.987	2.062	1.892	0.059
		1.456	1.018	2.083	2.055	0.040

Odds ratio (95% CI) with study removed

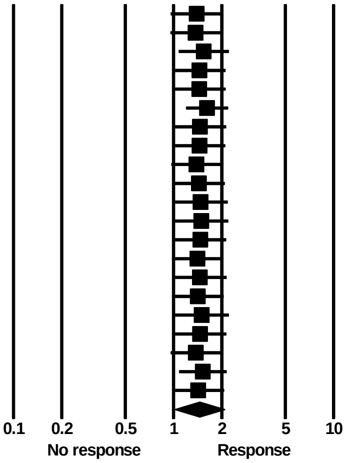

Meta Analysis

CI: confidence interval; mTORi: mTOR inhibitor

© 2022 Manothummetha K et al. *JAMA Network Open.*

Rituximab sensitivity analysis

Study name	Subgroup within study	Statistics with study removed				
		Point	Lower limit	Upper limit	Z-Value	p-Value
Grupper 2021	Rituximab	0.156	0.045	0.536	-2.946	0.003
Haskin 2021	Rituximab	0.356	0.091	1.395	-1.482	0.138
Rozen-Zvi 2021	Rituximab	0.223	0.069	0.723	-2.500	0.012
Sanders 2021	Rituximab	0.163	0.050	0.534	-2.999	0.003
Villanego 2021	Rituximab	0.208	0.065	0.662	-2.658	0.008
		0.207	0.070	0.614	-2.837	0.005

Odds ratio (95% CI) with study removed

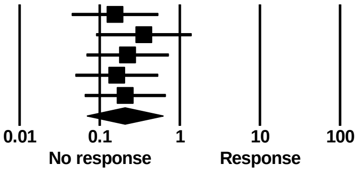

Meta Analysis

Body Mass Index sensitivity analysis

Study name	Subgroup within study	Statistics with study removed						
		Point	Standard error	Variance	Lower limit	Upper limit	Z-Value	p-Value
Grupper 2021	BMI	0.096	0.252	0.064	-0.399	0.590	0.379	0.704
Guarino 2021	BMI	0.298	0.244	0.060	-0.181	0.777	1.219	0.223
Hod 2021	BMI	0.067	0.235	0.055	-0.394	0.529	0.286	0.775
Masset 2021	BMI	0.246	0.281	0.079	-0.305	0.798	0.876	0.381
Midtvedth 2021	BMI	0.146	0.257	0.066	-0.357	0.649	0.570	0.568
Peled 2021	BMI	0.097	0.231	0.053	-0.355	0.550	0.421	0.674
Rabinowich 2021	BMI	0.115	0.244	0.060	-0.364	0.594	0.469	0.639
Rozen-Zvi 2021	BMI	0.225	0.273	0.075	-0.311	0.761	0.824	0.410
Sanders 2021	BMI	0.172	0.278	0.077	-0.374	0.717	0.618	0.537
		0.145	0.230	0.053	-0.306	0.596	0.629	0.529

Difference in means (95% CI) with study removed

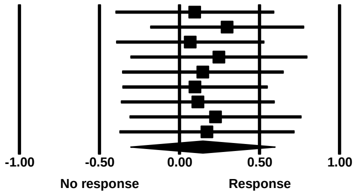

Meta Analysis

Age sensitivity analysis

Study name	Subgroup within study	Statistics with study removed						
		Point	Standard error	Variance	Lower limit	Upper limit	Z-Value	p-Value
Grupper 2021	Age	-3.681	1.195	1.427	-6.022	-1.339	-3.081	0.002
Guarino 2021	Age	-4.079	1.296	1.679	-6.619	-1.540	-3.148	0.002
Haskin 2021	Age	-4.478	0.704	0.495	-5.857	-3.099	-6.365	0.000
Hod 2021	Age	-4.122	1.234	1.523	-6.540	-1.703	-3.340	0.001
Korth 2021	Age	-4.025	1.166	1.360	-6.311	-1.740	-3.452	0.001
Masset 2021	Age	-4.247	1.369	1.875	-6.931	-1.564	-3.102	0.002
Midtvedth 2021	Age	-3.548	1.127	1.270	-5.757	-1.340	-3.149	0.002
Rabinowich 2021	Age	-3.815	1.207	1.457	-6.181	-1.450	-3.161	0.002
Rozen-Zvi 2021	Age	-3.648	1.199	1.437	-5.998	-1.299	-3.043	0.002
Sanders 2021	Age	-3.655	1.201	1.441	-6.008	-1.302	-3.044	0.002
		-3.942	1.134	1.286	-6.165	-1.719	-3.475	0.001

Difference in means (95% CI) with study removed

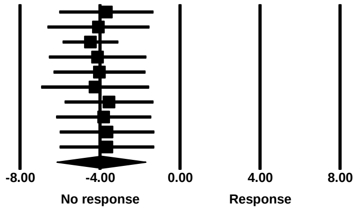

Meta Analysis

CI: confidence interval; BMI: body mass index

© 2022 Manothummetha K et al. *JAMA Network Open.*

Lymphocyte count sensitivity analysis

Study name	Subgroup within study	Point	Standard error	Variance	Lower limit	Upper limit	Z-Value	p-Value
Grupper 2021	Lymphocyte	0.014	0.136	0.018	-0.252	0.281	0.105	0.917
Hod 2021	Lymphocyte	0.348	0.127	0.016	0.099	0.596	2.743	0.006
Peled 2021	Lymphocyte	0.179	0.216	0.047	-0.244	0.602	0.828	0.407
Rabinowich 2021	Lymphocyte	0.159	0.236	0.056	-0.305	0.622	0.672	0.502
		0.174	0.168	0.028	-0.155	0.503	1.035	0.301

Statistics with study removed

Std diff in means (95% CI) with study removed

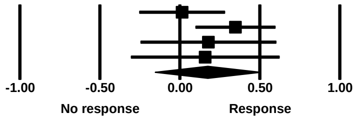

Meta Analysis

Duration after transplantation sensitivity analysis

Study name	Subgroup within study	Point	Standard error	Variance	Lower limit	Upper limit	Z-Value	p-Value
Grupper 2021	Time from transplant	0.301	0.103	0.011	0.099	0.503	2.922	0.003
Guarino 2021	Time from transplant	0.301	0.109	0.012	0.086	0.515	2.747	0.006
Haskin 2021	Time from transplant	0.295	0.096	0.009	0.106	0.484	3.061	0.002
Hod 2021	Time from transplant	0.344	0.089	0.008	0.168	0.519	3.844	0.000
Korth 2021	Time from transplant	0.284	0.091	0.008	0.105	0.463	3.111	0.002
Masset 2021	Time from transplant	0.261	0.098	0.010	0.069	0.453	2.661	0.008
Midtvedth 2021	Time from transplant	0.257	0.090	0.008	0.081	0.433	2.861	0.004
Rabinowich 2021	Time from transplant	0.307	0.100	0.010	0.111	0.503	3.071	0.002
Rozen-Zvi 2021	Time from transplant	0.359	0.080	0.006	0.203	0.516	4.496	0.000
		0.300	0.090	0.008	0.123	0.477	3.318	0.001

Statistics with study removed

Std diff in means (95% CI) with study removed

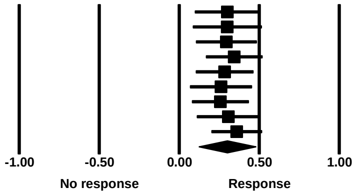

Meta Analysis

CI: confidence interval

© 2022 Manothummetha K et al. *JAMA Network Open.*

eFigure 3. Publication Bias of Studied Risk Factors

Antimetabolite

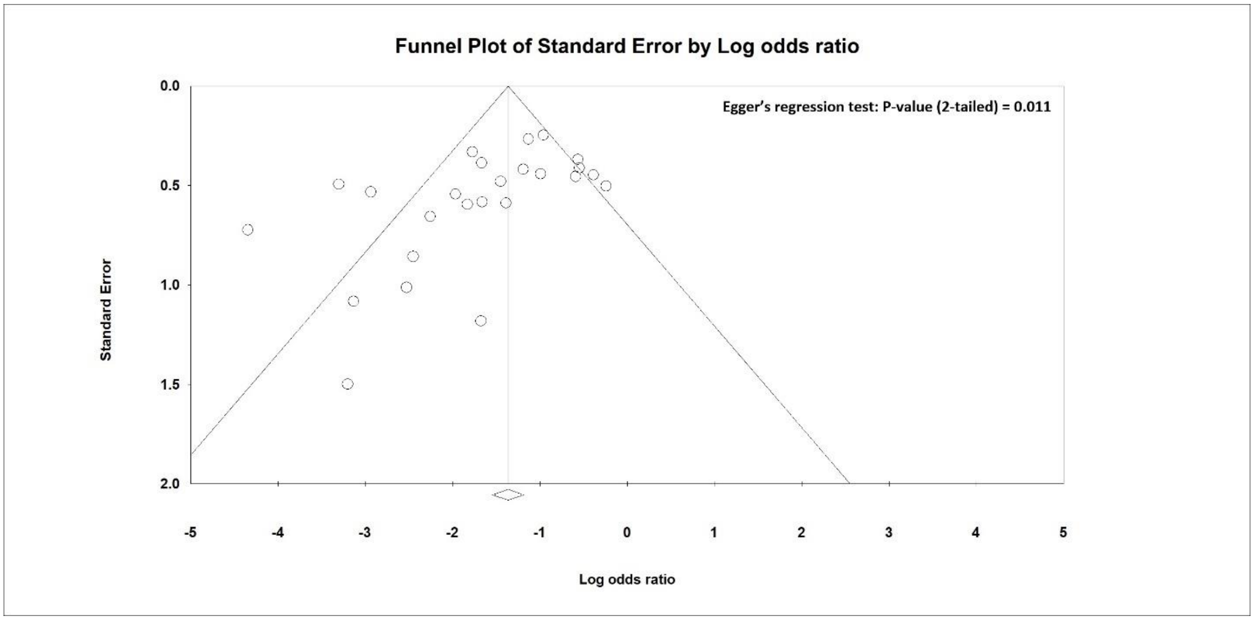

Anti-thymocyte globulin

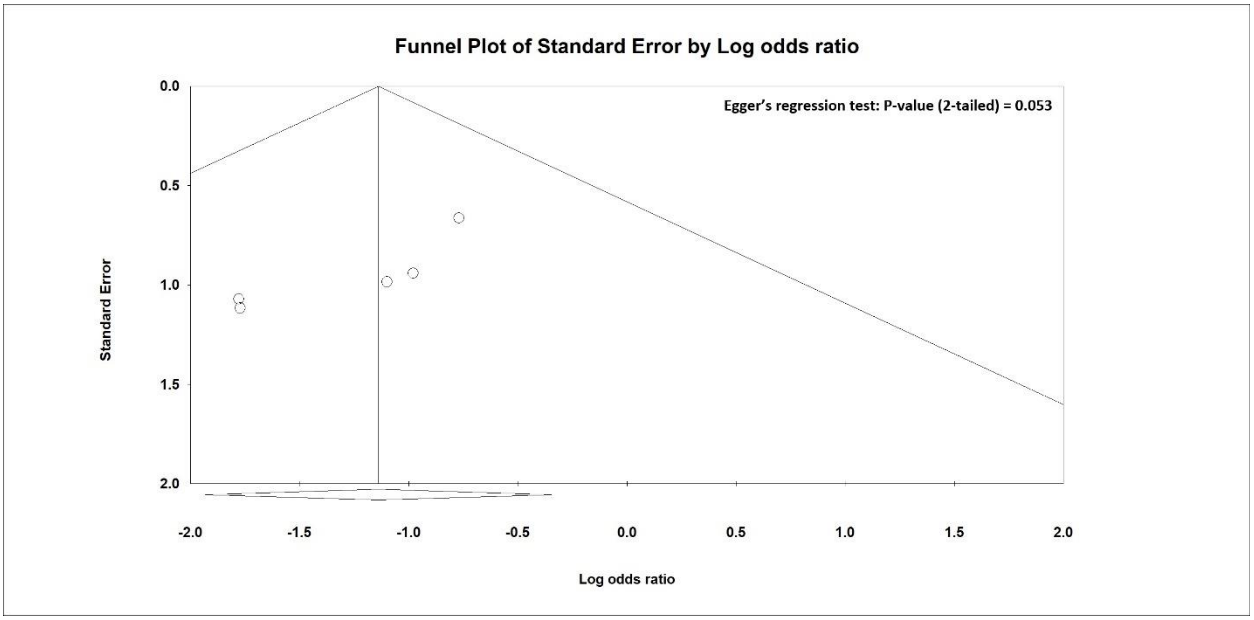

© 2022 Manothummetha K et al. *JAMA Network Open.*

Calcineurin inhibitor

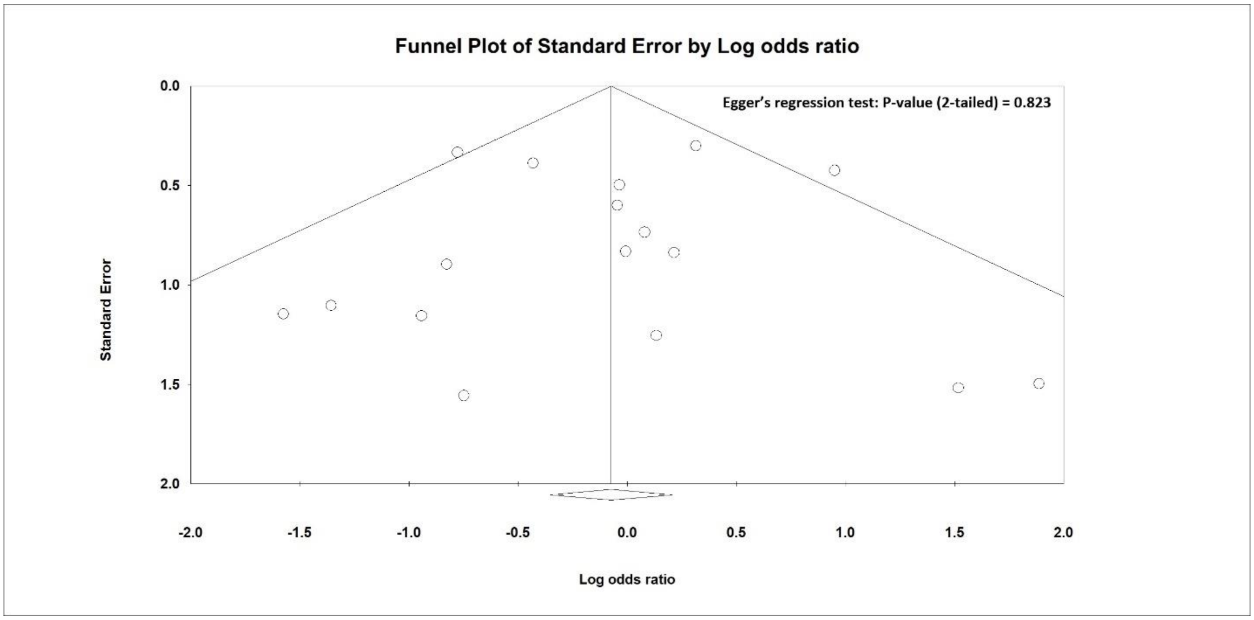

Deceased donor

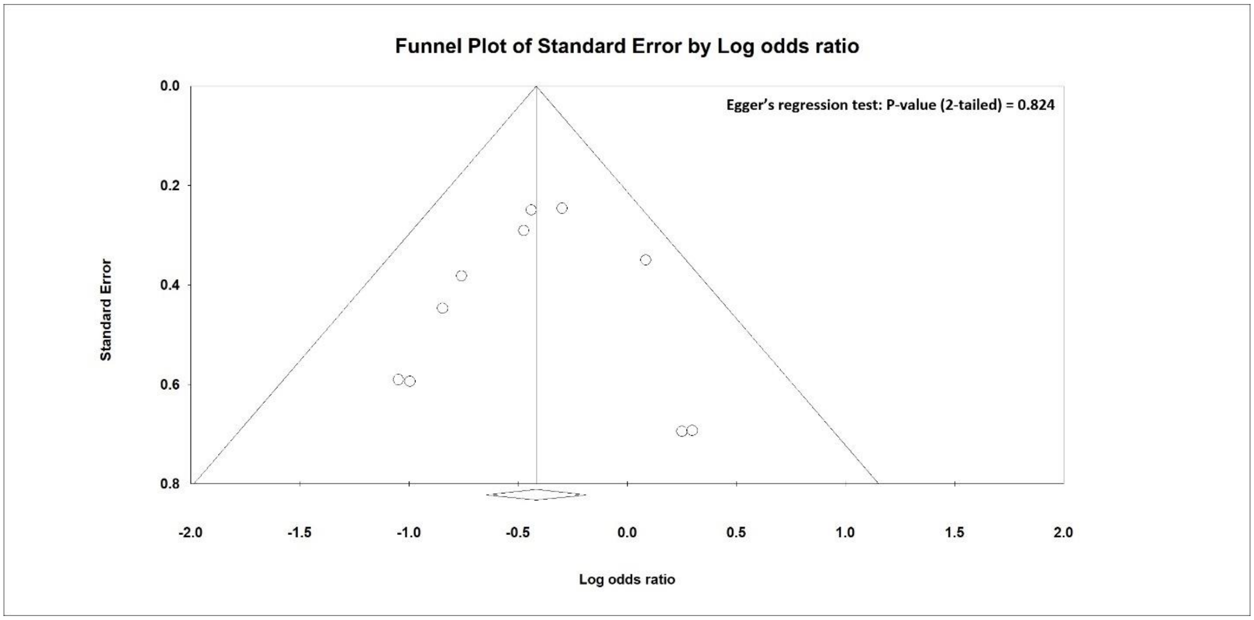

© 2022 Manothummetha K et al. *JAMA Network Open.*

Male

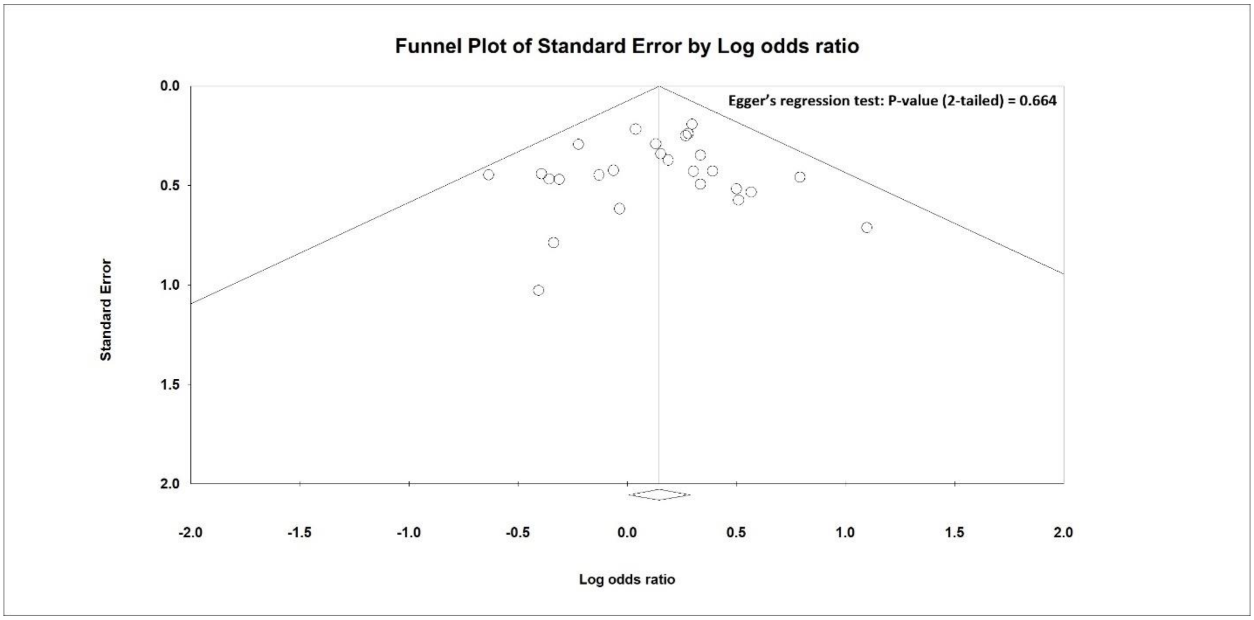

mTOR inhibitor

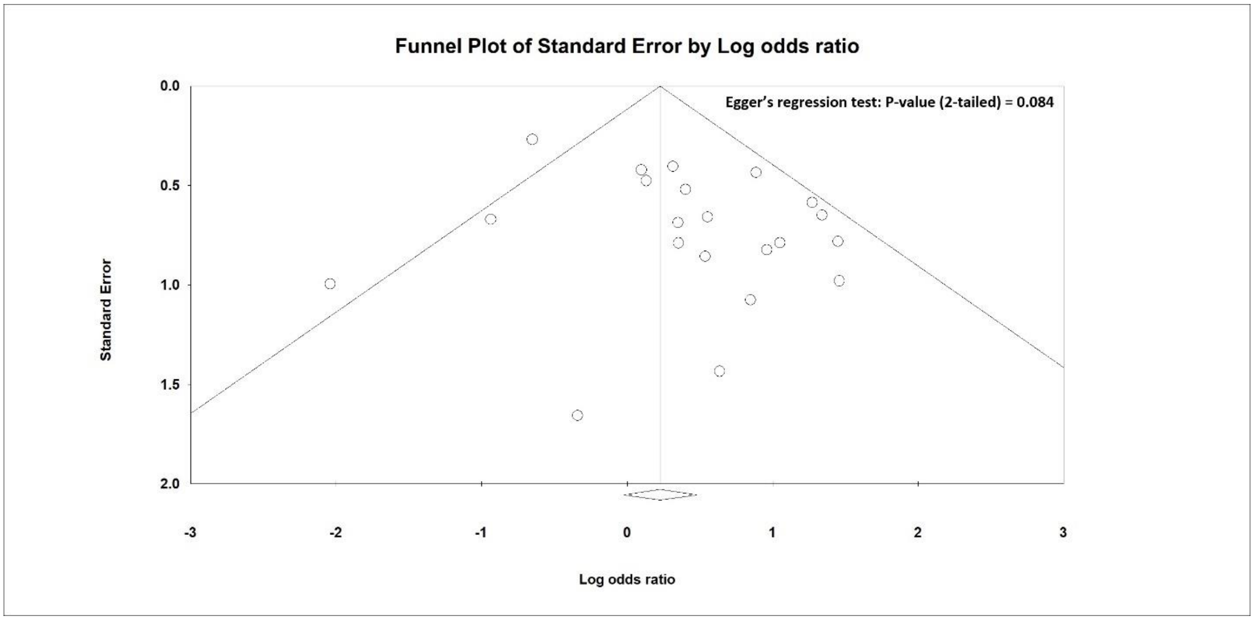

© 2022 Manothummetha K et al. *JAMA Network Open.*

Rituximab

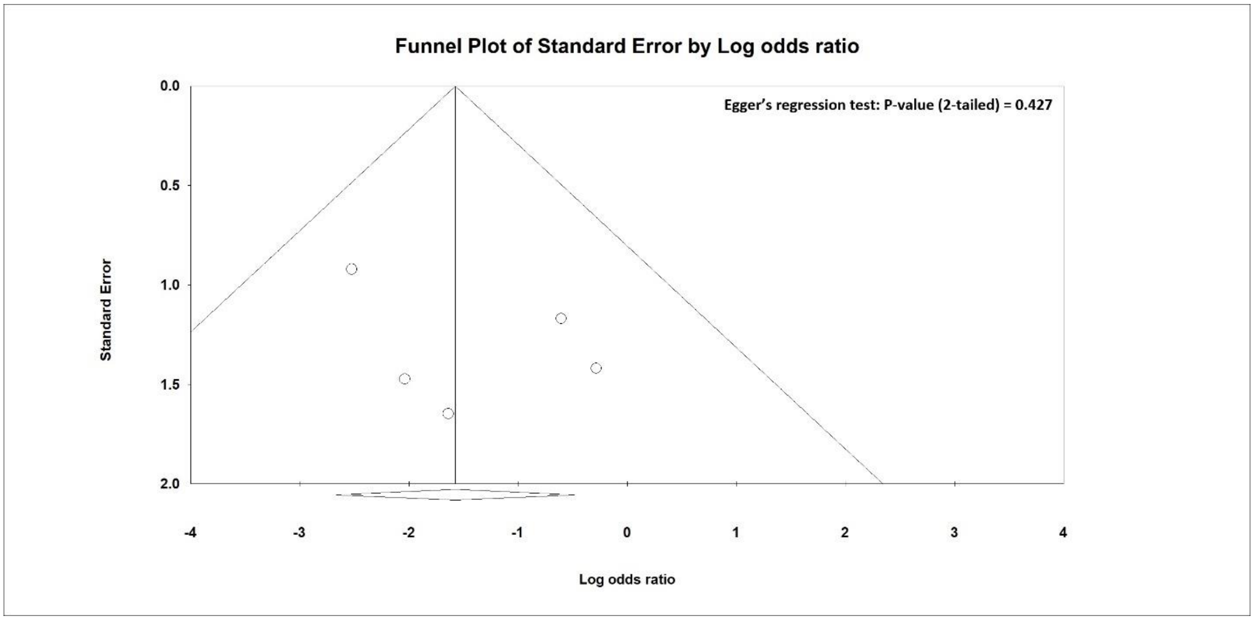

Age

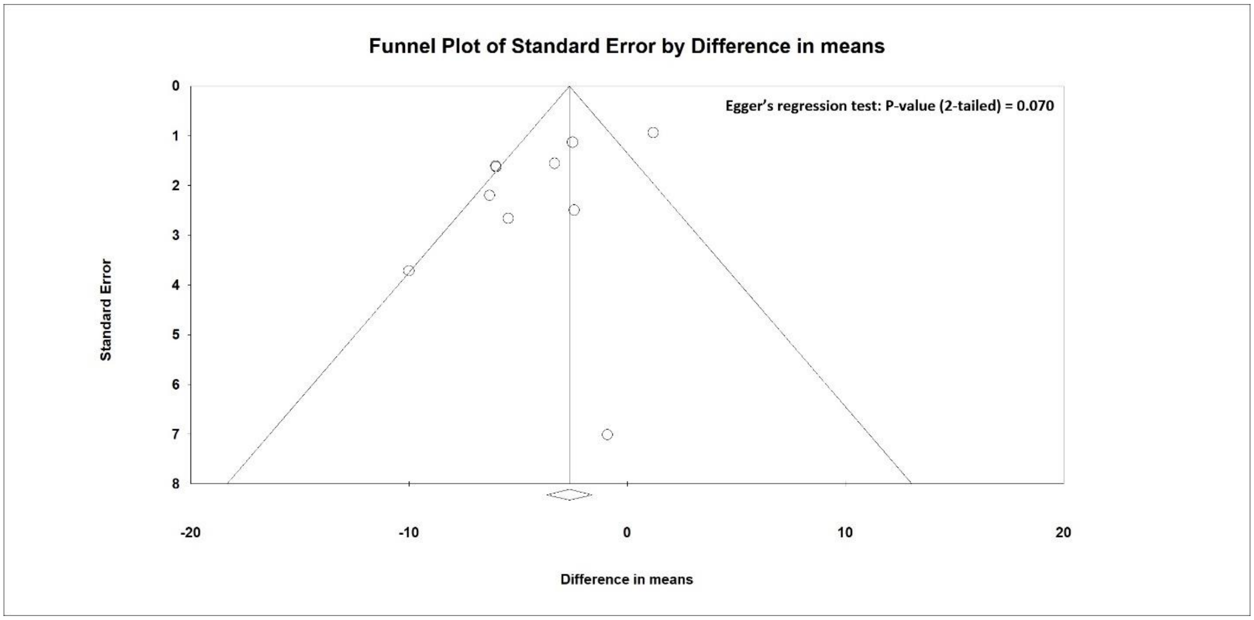

© 2022 Manothummetha K et al. *JAMA Network Open.*

BMI

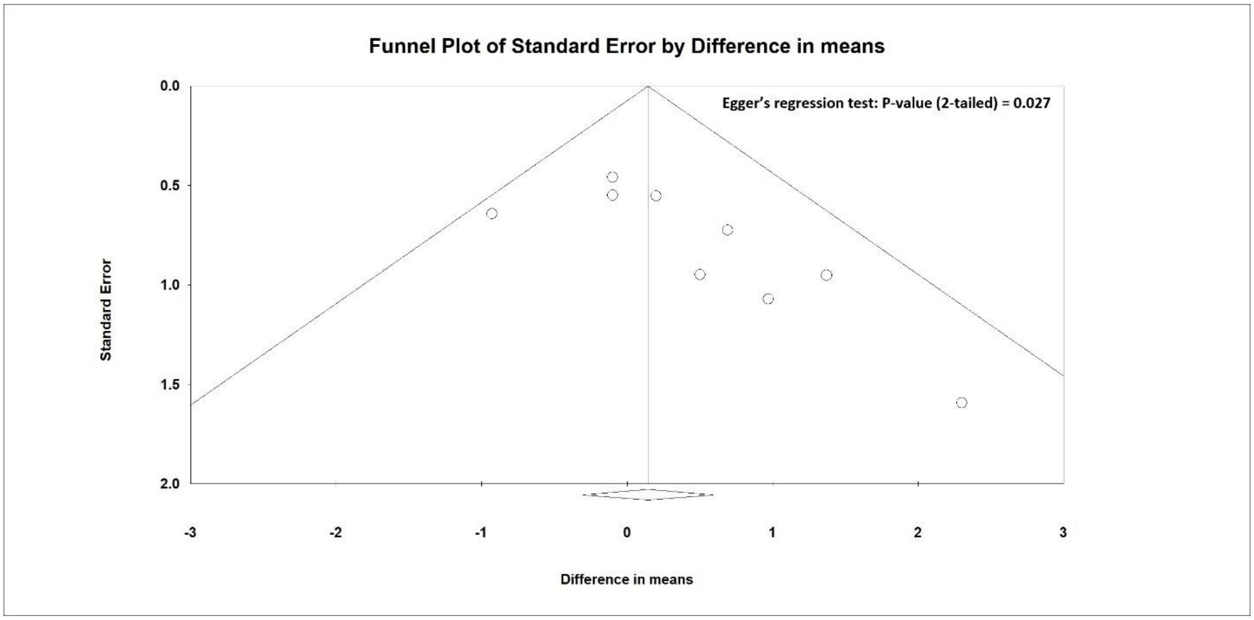

Lymphocyte count

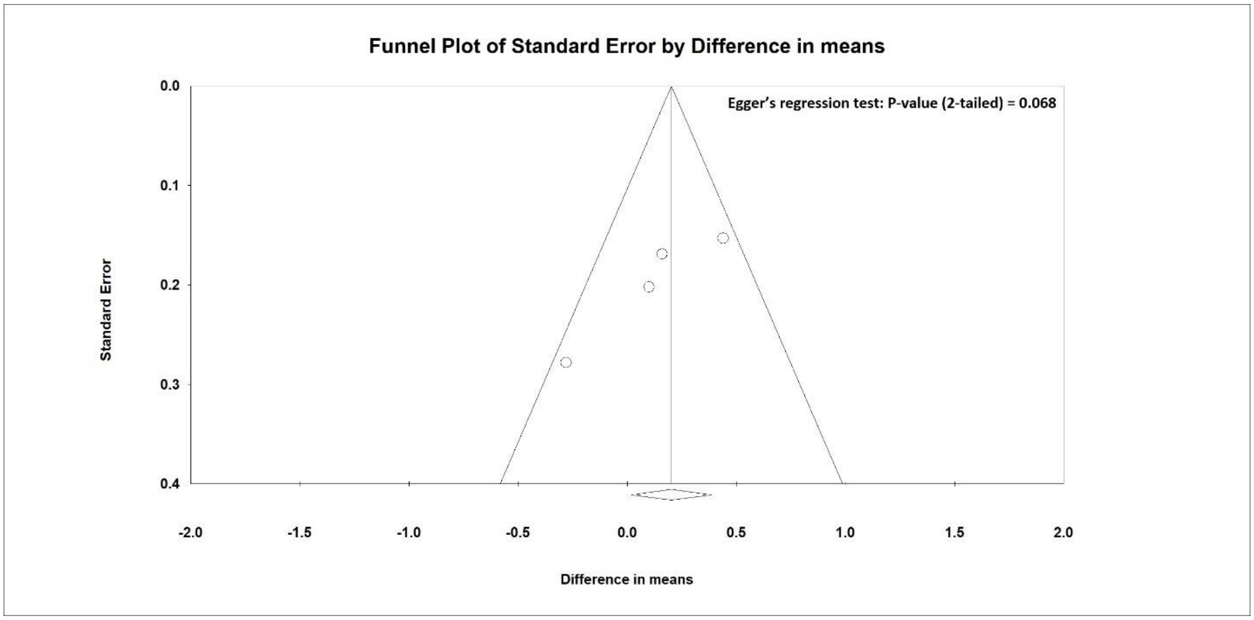

© 2022 Manothummetha K et al. *JAMA Network Open.*

Duration after transplantation

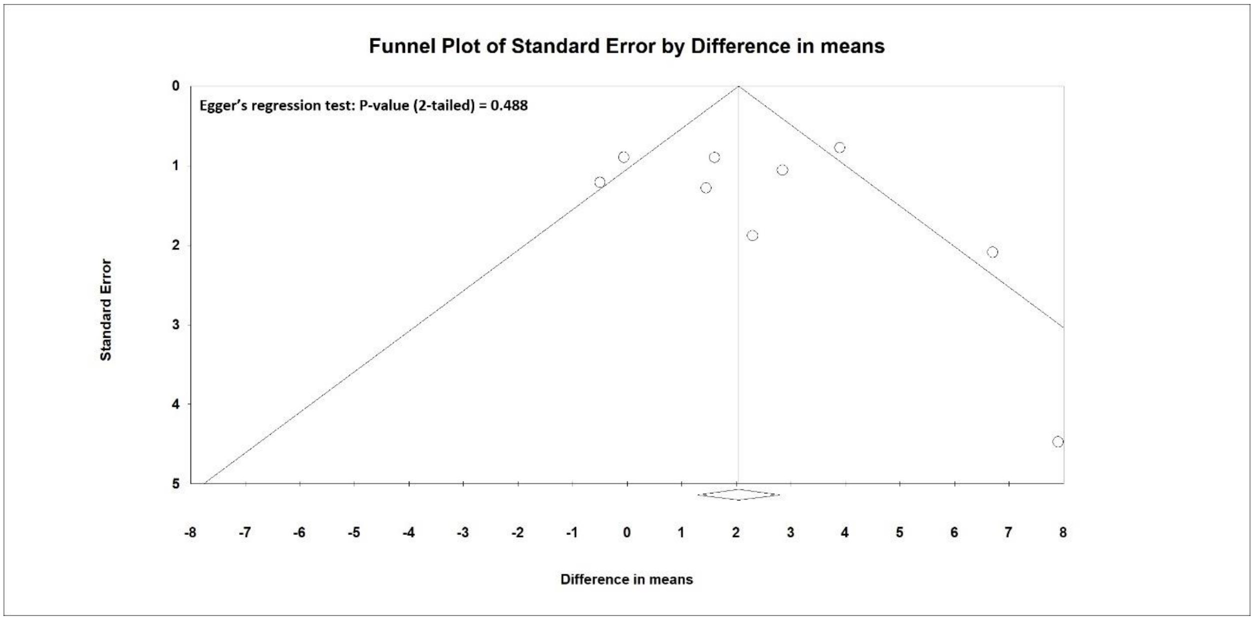

© 2022 Manothummetha K et al. *JAMA Network Open.*

eFigure 4. Funnel Plots and Adjusted Effect Estimates Accounting for Publication Bias

Antimetabolite

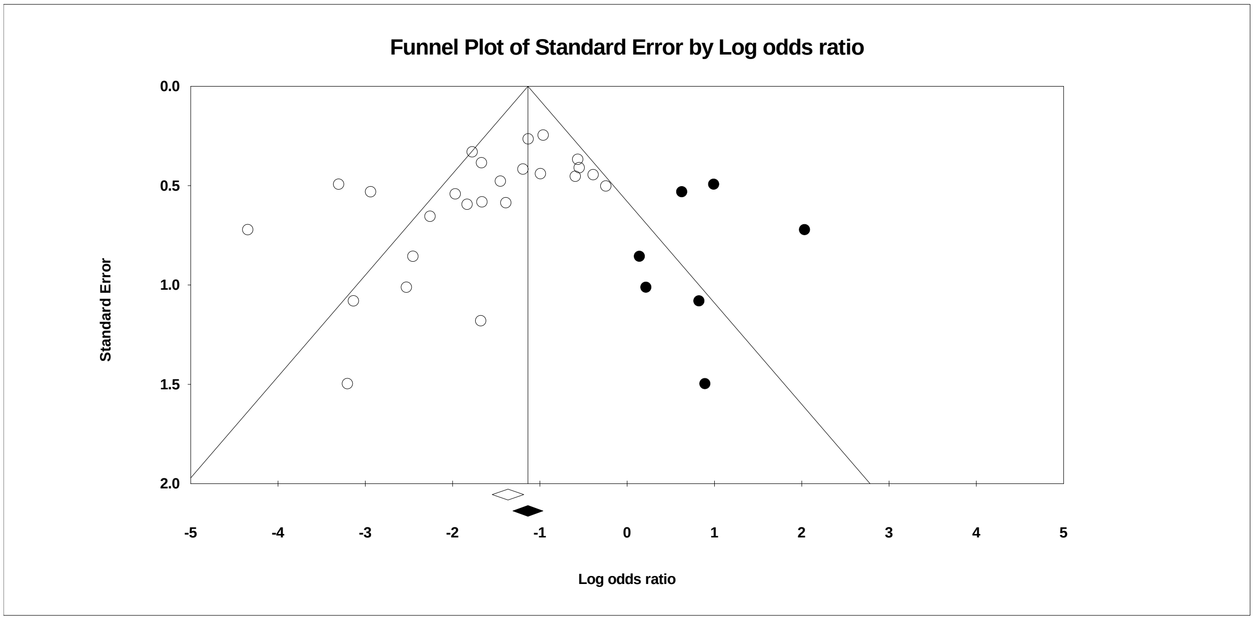

Anti-thymocyte globulin

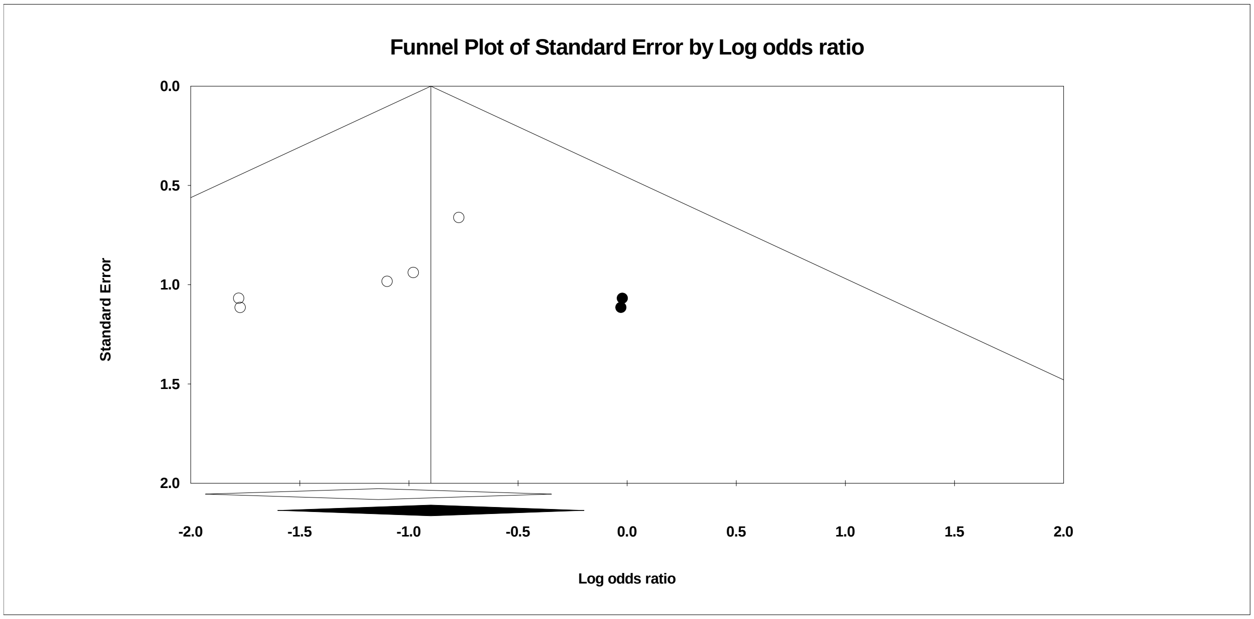

© 2022 Manothummetha K et al. *JAMA Network Open.*

mTOR inhibitor

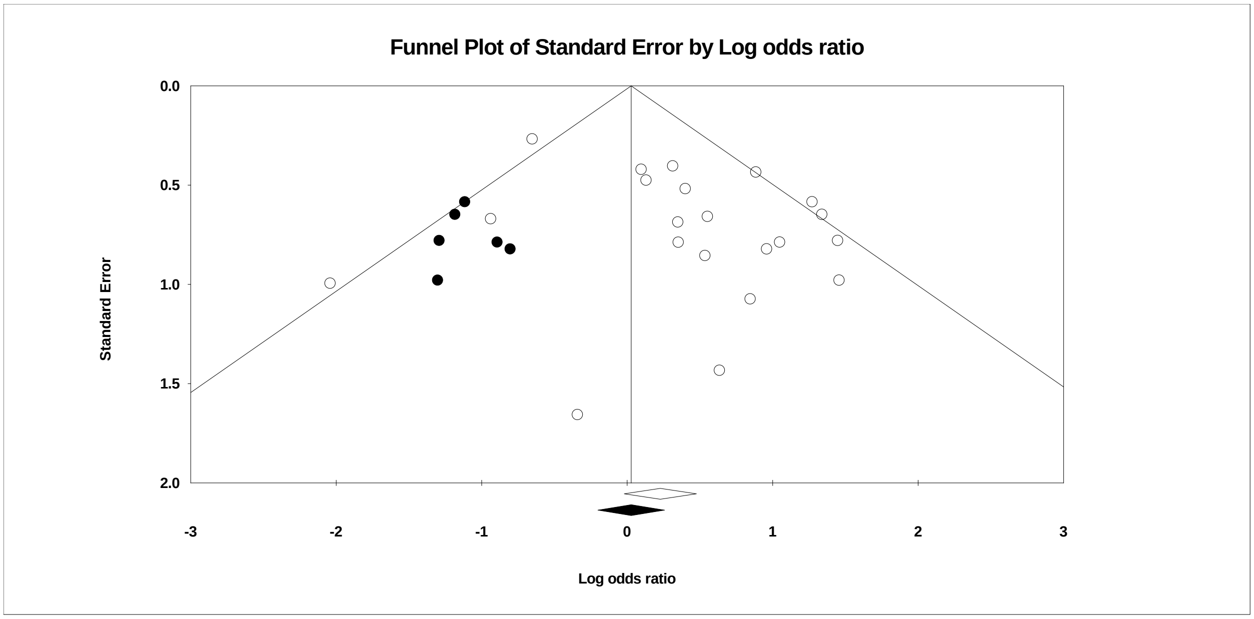

BMI

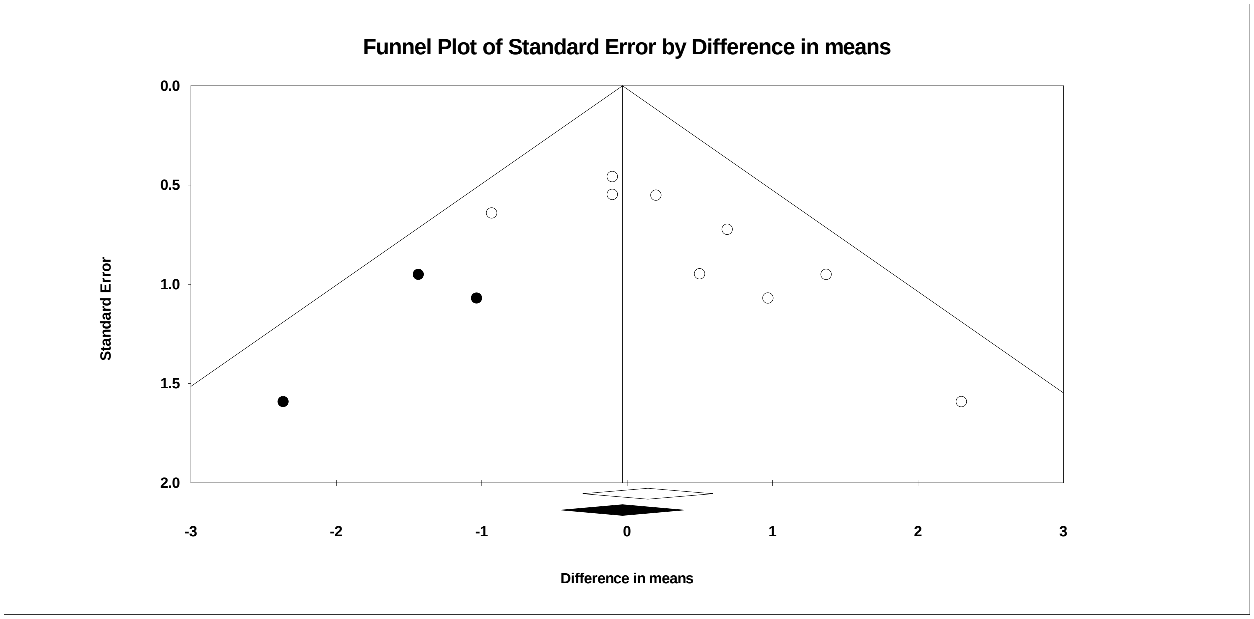

© 2022 Manothummetha K et al. *JAMA Network Open.*

Lymphocyte count

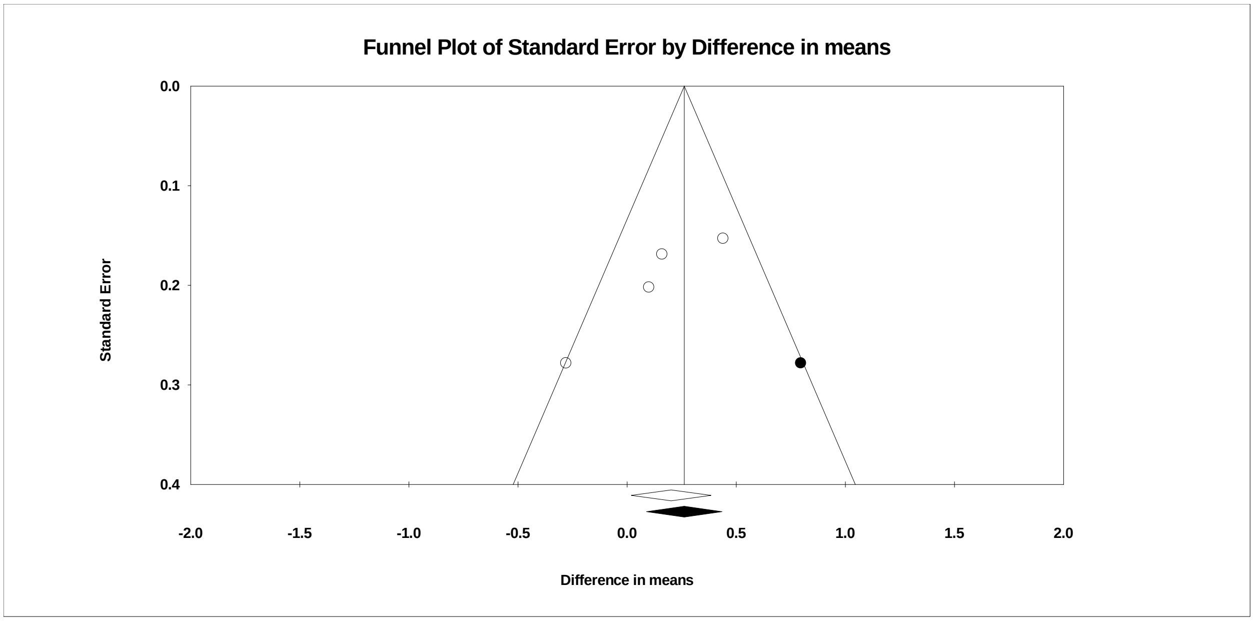

Duration after transplantation

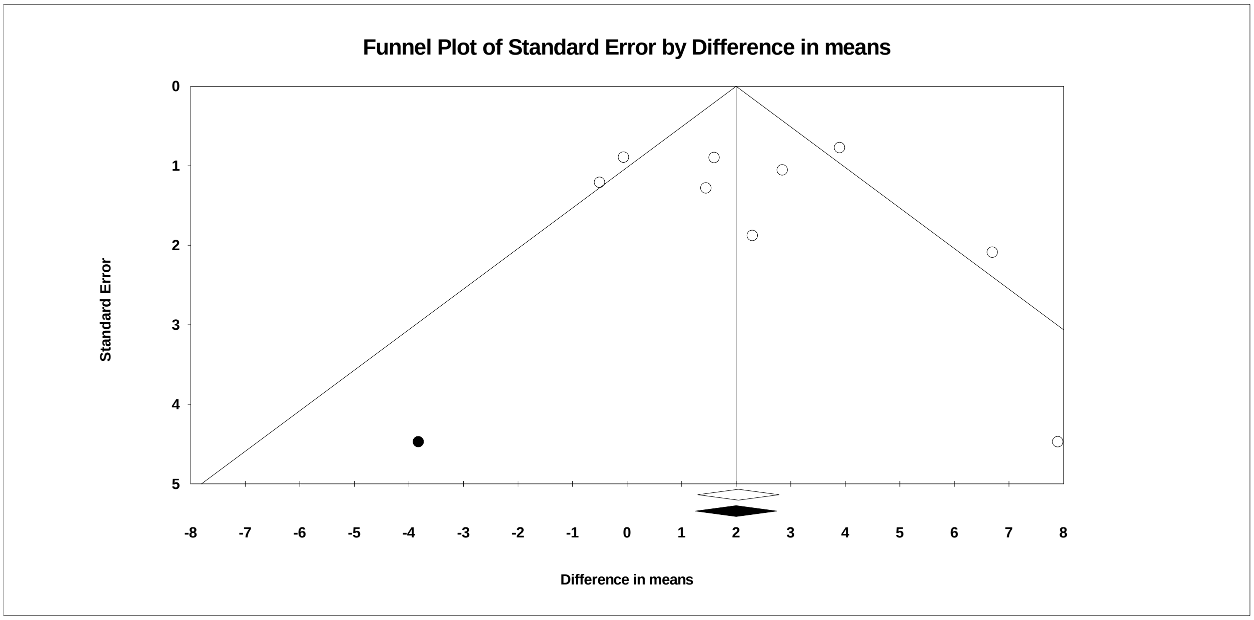

© 2022 Manothummetha K et al. *JAMA Network Open.*

eReferences

1. Alejo JL, Mitchell J, Chiang TPY, et al. Antibody Response to a Fourth Dose of a SARS-CoV-2 Vaccine in Solid Organ Transplant Recipients: A Case Series. *Transplantation*. 2021;(wej, 0132144). doi:10.1097/TP.0000000000003934

2. Azzi Y, Raees H, Wang T, et al. Risk factors associated with poor response to COVID-19 vaccination in kidney transplant recipients. *Kidney Int*. Published online September 3, 2021:S0085-2538(21)00826-7. doi:10.1016/j.kint.2021.08.019

3. Itzhaki Ben Zadok O, Shaul AA, Ben-Avraham B, et al. Immunogenicity of the BNT162b2 mRNA vaccine in heart transplant recipients - a prospective cohort study. *European journal of heart failure*. 2021;(dr4, 100887595). doi:10.1002/ejhf.2199

4. Benotmane I, Gautier-Vargas G, Cognard N, et al. Weak anti-SARS-CoV-2 antibody response after the first injection of an mRNA COVID-19 vaccine in kidney transplant recipients. *KIDNEY INTERNATIONAL*. 2021;99(6):1487-1489. doi:10.1016/j.kint.2021.03.014

5. Benotmane I, Gautier-Vargas G, Cognard N, et al. Low immunization rates among kidney transplant recipients who received 2 doses of the mRNA-1273 SARS-CoV-2 vaccine. *Kidney international*. 2021;99(6):1498-1500. doi:10.1016/j.kint.2021.04.005

6. Benotmane I, Gautier G, Perrin P, et al. Antibody Response After a Third Dose of the mRNA-1273 SARS-CoV-2 Vaccine in Kidney Transplant Recipients With Minimal Serologic Response to 2 Doses. *JAMA-JOURNAL OF THE AMERICAN MEDICAL ASSOCIATION*. doi:10.1001/jama.2021.12339

7. Bergman P, Blennow O, Hansson L, et al. Safety and efficacy of the mRNA BNT162b2 vaccine against SARS-CoV-2 in five groups of immunocompromised patients and healthy controls in a prospective open-label clinical trial. *EBioMedicine*. 2021;74. doi:10.1016/j.ebiom.2021.103705

8. Bertrand D, Hamzaoui M, Lemee V, et al. Antibody and T Cell Response to SARS-CoV-2 Messenger RNA BNT162b2 Vaccine in Kidney Transplant Recipients and Hemodialysis Patients. *Journal of the American Society of Nephrology : JASN*. 2021;32(9):2147-2152. doi:10.1681/ASN.2021040480

9. Bertrand D, Hanoy M, Edet S, et al. Antibody response to SARS-CoV-2 mRNA BNT162b2 vaccine in kidney transplant recipients and in-centre and satellite centre haemodialysis patients. *Clinical kidney journal*. 2021;14(9):2127-2128. doi:10.1093/ckj/sfab100

10. Bertrand D, Hamzaoui M, Lemée V, et al. Antibody and T cell response to a third dose of SARS-CoV-2 messenger RNA BNT162b2 vaccine in kidney transplant recipients. *Kidney Int*. Published online October 4, 2021:S0085-2538(21)00924-8. doi:10.1016/j.kint.2021.09.014

© 2022 Manothummetha K et al. *JAMA Network Open.*

11. Boyarsky BJ, Werbel WA, Avery RK, et al. Immunogenicity of a Single Dose of SARS-CoV-2 Messenger RNA Vaccine in Solid Organ Transplant Recipients. *JAMA-JOURNAL OF THE AMERICAN MEDICAL ASSOCIATION*. 2021;325(17):1784-1786. doi:10.1001/jama.2021.4385

12. Boyarsky BJ, Werbel WA, Avery RK, et al. Antibody Response to 2-Dose SARS-CoV-2 mRNA Vaccine Series in Solid Organ Transplant Recipients. *JAMA-JOURNAL OF THE AMERICAN MEDICAL ASSOCIATION*. 2021;325(21):2204-2206. doi:10.1001/jama.2021.7489

13. Boyarsky BJ, Chiang TPY, Ou MT, et al. Antibody Response to the Janssen COVID-19 Vaccine in Solid Organ Transplant Recipients. *TRANSPLANTATION*. 2021;105(8):E82-E83. doi:10.1097/TP.0000000000003850

14. Cao J, Liu X, Muthukumar A, Gagan J, Jones P, Zu Y. Poor Humoral Response in Solid Organ Transplant Recipients following Complete mRNA SARS-CoV-2 Vaccination. *Clinical chemistry*. 2021;(dbz, 9421549). doi:10.1093/clinchem/hvab149

15. Chavarot N, Ouedrani A, Marion O, et al. Poor Anti-SARS-CoV-2 Humoral and T-cell Responses After 2 Injections of mRNA Vaccine in Kidney Transplant Recipients Treated With Belatacept. *TRANSPLANTATION*. 2021;105(9):E94-E95. doi:10.1097/TP.0000000000003784

16. Chavarot N, Morel A, Leruez-Ville M, et al. Weak antibody response to three doses of mRNA vaccine in kidney transplant recipients treated with belatacept. *AMERICAN JOURNAL OF TRANSPLANTATION*. doi:10.1111/ajt.16814

17. Crespo M, Barrilado-Jackson A, Padilla E, et al. Negative immune responses to two-dose mRNA COVID-19 vaccines in renal allograft recipients assessed with simple antibody and interferon gamma release assay cellular monitoring. *Am J Transplant*. Published online September 22, 2021. doi:10.1111/ajt.16854

18. Cucchiari D, Egri N, Bodro M, et al. Cellular and humoral response after MRNA-1273 SARS-CoV-2 vaccine in kidney transplant recipients. *American journal of transplantation : official journal of the American Society of Transplantation and the American Society of Transplant Surgeons*. 2021;21(8):2727-2739. doi:10.1111/ajt.16701

19. D’Offizi G, Agrati C, Visco-Comandini U, et al. Coordinated cellular and humoral immune responses after two-dose SARS-CoV2 mRNA vaccination in liver transplant recipients. *Liver International*. n/a(n/a). doi:10.1111/liv.15089

20. Danthu C, Hantz S, Dahlem A, et al. Humoral Response after SARS-CoV-2 mRNA Vaccination in a Cohort of Hemodialysis Patients and Kidney Transplant Recipients. *Journal of the American Society of Nephrology : JASN*. 2021;32(9):2153-2158. doi:10.1681/ASN.2021040490

© 2022 Manothummetha K et al. *JAMA Network Open.*

21. Davidov Y, Tsaraf K, Cohen-Ezra O, et al. Immunogenicity and Adverse Effects of the 2-Dose BNT162b2 Messenger RNA Vaccine Among Liver Transplantation Recipients. *Liver Transplantation*. n/a(n/a). doi:10.1002/lt.26366

22. Dębska-Ślizień A, Ślizień Z, Muchlado M, et al. Predictors of Humoral Response to mRNA COVID19 Vaccines in Kidney Transplant Recipients: A Longitudinal Study—The COViNEPH Project. *Vaccines*. 2021;9(10):1165. doi:10.3390/vaccines9101165

23. Del Bello A, Abravanel F, Marion O, et al. Efficiency of a boost with a third dose of anti-SARS-CoV-2 messenger RNA-based vaccines in solid organ transplant recipients. *AMERICAN JOURNAL OF TRANSPLANTATION*. doi:10.1111/ajt.16775

24. Ducloux D, Colladant M, Chabannes M, Bamoulid J, Courivaud C. Factors associated with humoral response after BNT162b2 mRNA COVID-19 vaccination in kidney transplant patients. *Clinical Kidney Journal*. 2021;14(10):2270-2272. doi:10.1093/ckj/sfab125

25. Firket L, Descy J, Seidel L, et al. Serological response to mRNA SARS-CoV-2 BNT162b2 vaccine in kidney transplant recipients depends on prior exposure to SARS-CoV-2. *American journal of transplantation : official journal of the American Society of Transplantation and the American Society of Transplant Surgeons*. 2021;(100968638). doi:10.1111/ajt.16726

26. Georgery H, Devresse A, Yombi JC, et al. Very low Immunization Rate in Kidney Transplant Recipients after one Dose of the BNT162b2 Vaccine: Beware not to Lower the Guard!. *Transplantation*. 2021;(wej, 0132144). doi:10.1097/TP.0000000000003818

27. Georgery H, Devresse A, Yombi JC, et al. Disappointing Immunization Rate After 2 Doses of the BNT162b2 Vaccine in a Belgian Cohort of Kidney Transplant Recipients. *Transplantation*. 2021;(wej, 0132144). doi:10.1097/TP.0000000000003861

28. Grupper A, Rabinowich L, Schwartz D, et al. Reduced humoral response to mRNA SARS-CoV-2 BNT162b2 vaccine in kidney transplant recipients without prior exposure to the virus. *American journal of transplantation : official journal of the American Society of Transplantation and the American Society of Transplant Surgeons*. 2021;21(8):2719-2726. doi:10.1111/ajt.16615

29. Guarino M, Cossiga V, Esposito I, Alessandro F, Morisco F. Effectiveness of SARS-Cov-2 vaccination in liver transplanted patients: the debate is open!. *Journal of hepatology*. 2021;(ibs, 8503886). doi:10.1016/j.jhep.2021.07.034

30. Hall VG, Ferreira VH, Ierullo M, et al. Humoral and cellular immune response and safety of two-dose SARS-CoV-2 mRNA-1273 vaccine in solid organ transplant recipients. *American journal of transplantation : official journal of the American Society of Transplantation and the American Society of Transplant Surgeons*. 2021;(100968638). doi:10.1111/ajt.16766

© 2022 Manothummetha K et al. *JAMA Network Open.*

31. Hall V, Ferreira V, Ku T, et al. Randomized Trial of a Third Dose of mRNA-1273 Vaccine in Transplant Recipients. *NEW ENGLAND JOURNAL OF MEDICINE*. 2021;385(13):1244-1246. doi:10.1056/NEJMc2111462

32. Haskin O, Ashkenazi-Hoffnung L, Ziv N, et al. Serological Response to the BNT162b2 COVID-19 mRNA Vaccine in Adolescent and Young Adult Kidney Transplant Recipients. *Transplantation*. 2021;(wej, 0132144). doi:10.1097/TP.0000000000003922

33. Havlin J, Svorcova M, Dvorackova E, et al. Immunogenicity of BNT162b2 mRNA COVID-19 vaccine and SARS-CoV-2 infection in lung transplant recipients. *The Journal of heart and lung transplantation : the official publication of the International Society for Heart Transplantation*. 2021;40(8):754-758. doi:10.1016/j.healun.2021.05.004

34. Havlin J, Skotnicova A, Dvorackova E, et al. Impaired Humoral Response to Third Dose of BNT162b2 mRNA COVID-19 Vaccine Despite Detectable Spike Protein–specific T cells in Lung Transplant Recipients. *Transplantation*. Published online December 17, 2021. doi:10.1097/TP.0000000000004021

35. Herrera S, Colmenero J, Pascal M, et al. Cellular and humoral immune response after mRNA-1273 SARS-CoV-2 vaccine in liver and heart transplant recipients. *American journal of transplantation : official journal of the American Society of Transplantation and the American Society of Transplant Surgeons*. 2021;(100968638). doi:10.1111/ajt.16768

36. Hod T, Ben-David A, Olmer L, et al. Humoral Response of Renal Transplant Recipients to the BNT162b2 SARS-CoV-2 mRNA Vaccine Using Both RBD IgG and Neutralizing Antibodies. *Transplantation*. 2021;(wej, 0132144). doi:10.1097/TP.0000000000003889

37. Hoffman TW, Meek B, Rijkers GT, van Kessel DA. Poor Serologic Response to 2 Doses of an mRNA-based SARS-CoV-2 Vaccine in Lung Transplant Recipients. *Transplantation*. 2022;106(1):e103. doi:10.1097/TP.0000000000003966

38. Holden IK, Bistrup C, Nilsson AC, et al. Immunogenicity of SARS-CoV-2 mRNA vaccine in solid organ transplant recipients. *Journal of internal medicine*. 2021;(i2g, 8904841). doi:10.1111/joim.13361

39. Kamar N, Abravanel F, Marion O, Couat C, Izopet J, Del Bello A. Three Doses of an mRNA Covid-19 Vaccine in Solid-Organ Transplant Recipients. *The New England journal of medicine*. 2021;385(7):661-662. doi:10.1056/NEJMc2108861

40. Kamar N, Abravanel F, Marion O, et al. Assessment of 4 Doses of SARS-CoV-2 Messenger RNA–Based Vaccine in Recipients of a Solid Organ Transplant. *JAMA Network Open*. 2021;4(11):e2136030. doi:10.1001/jamanetworkopen.2021.36030

41. Kantauskaite M, Müller L, Kolb T, et al. Intensity of mycophenolate mofetil treatment is associated with an impaired immune response to SARS-CoV-2 vaccination in kidney

© 2022 Manothummetha K et al. *JAMA Network Open.*

transplant recipients. *Am J Transplant*. Published online September 22, 2021. doi:10.1111/ajt.16851

42. Korth J, Jahn M, Dorsch O, et al. Impaired Humoral Response in Renal Transplant Recipients to SARS-CoV-2 Vaccination with BNT162b2 (Pfizer-BioNTech). *Viruses*. 2021;13(5). doi:10.3390/v13050756

43. Kumar D, Ferreira VH, Hall VG, et al. Neutralization of SARS-CoV-2 Variants in Transplant Recipients After Two and Three Doses of mRNA-1273 Vaccine. *Ann Intern Med*. Published online November 23, 2021. doi:10.7326/M21-3480

44. Marinaki S, Adamopoulos S, Degiannis D, et al. Immunogenicity of SARS-CoV-2 BNT162b2 vaccine in solid organ transplant recipients. *American journal of transplantation : official journal of the American Society of Transplantation and the American Society of Transplant Surgeons*. 2021;21(8):2913-2915. doi:10.1111/ajt.16607

45. Marion O, Del Bello A, Abravanel F, et al. Safety and Immunogenicity of Anti-SARS-CoV-2 Messenger RNA Vaccines in Recipients of Solid Organ Transplants. *Annals of internal medicine*. 2021;(0372351). doi:10.7326/M21-1341

46. Massa F, Cremoni M, Gérard A, et al. Safety and cross-variant immunogenicity of a three-dose COVID-19 mRNA vaccine regimen in kidney transplant recipients. *EBioMedicine*. 2021;73:103679. doi:10.1016/j.ebiom.2021.103679

47. Masset C, Kerleau C, Garandeau C, et al. A third injection of BNT162b2 mRNA Covid-19 vaccine in kidney transplant recipients improves the humoral immune response. *Kidney international*. 2021;(kvb, 0323470). doi:10.1016/j.kint.2021.08.017

48. Masset C, Ville S, Garandeau C, et al. Observations on improving COVID-19 vaccination responses in kidney transplant recipients: heterologous vaccination and immunosuppression modulation. *Kidney International*. Published online December 8, 2021. doi:10.1016/j.kint.2021.11.024

49. Mazzola A, Todesco E, Drouin S, et al. Poor Antibody Response after Two Doses of SARS-CoV-2 vaccine in Transplant Recipients. *Clinical infectious diseases : an official publication of the Infectious Diseases Society of America*. 2021;(a4j, 9203213). doi:10.1093/cid/ciab580

50. Medina-Pestana J, Cristelli MP, Viana LA, et al. Clinical Impact, Reactogenicity, and Immunogenicity After the First CoronaVac Dose in Kidney Transplant Recipients. *Transplantation*. 2021;(wej, 0132144). doi:10.1097/TP.0000000000003901

51. Middleton RJ, Gorton J, O'Riordan E, Knight S, Kalra PA, Poulikakos D. Impact of Shielding and First Dose of COVID-19 Vaccination in Kidney Transplant Recipients. *NEPHRON*. doi:10.1159/000518631

© 2022 Manothummetha K et al. *JAMA Network Open.*

52. Midtvedt K, Tran T, Parker K, et al. Low Immunization Rate in Kidney Transplant Recipients Also After Dose 2 of the BNT162b2 Vaccine: Continue to Keep Your Guard up! *TRANSPLANTATION*. 2021;105(8):E80-E81. doi:10.1097/TP.0000000000003856

53. Miele M, Busa R, Russelli G, et al. Impaired anti-SARS-CoV-2 humoral and cellular immune response induced by Pfizer-BioNTech BNT162b2 mRNA vaccine in solid organ transplanted patients. *American journal of transplantation : official journal of the American Society of Transplantation and the American Society of Transplant Surgeons*. 2021;21(8):2919-2921. doi:10.1111/ajt.16702

54. Narasimhan M, Mahimainathan L, Clark AE, et al. Serological Response in Lung Transplant Recipients after Two Doses of SARS-CoV-2 mRNA Vaccines. *Vaccines*. 2021;9(7). doi:10.3390/vaccines9070708

55. Noble J, Langello A, Bouchut W, Lupo J, Lombardo D, Rostaing L. Immune Response Post-SARS-CoV-2 mRNA Vaccination in Kidney-Transplant Recipients Receiving Belatacept. *Transplantation*. 2021;(wej, 0132144). doi:10.1097/TP.0000000000003923

56. Pedersen RM, Bang LL, Tornby DS, et al. The SARS-CoV-2-neutralizing capacity of kidney transplant recipients 4 weeks after receiving a second dose of the BNT162b2 vaccine. *Kidney Int*. Published online September 20, 2021:S0085-2538(21)00866-8. doi:10.1016/j.kint.2021.09.006

57. Peled Y, Ram E, Lavee J, et al. BNT162b2 vaccination in heart transplant recipients: Clinical experience and antibody response. *The Journal of heart and lung transplantation : the official publication of the International Society for Heart Transplantation*. 2021;40(8):759-762. doi:10.1016/j.healun.2021.04.003

58. Peled Y, Ram E, Lavee J, et al. Third dose of the BNT162b2 vaccine in heart transplant recipients: Immunogenicity and clinical experience. *J Heart Lung Transplant*. Published online August 28, 2021:S1053-2498(21)02481-5. doi:10.1016/j.healun.2021.08.010

59. Prendecki M, Thomson T, Clarke CL, et al. Immunological responses to SARS-CoV-2 vaccines in kidney transplant recipients. *Lancet*. Published online October 4, 2021:S0140-6736(21)02096-1. doi:10.1016/S0140-6736(21)02096-1

60. Quiroga B, Soler MJ, Ortiz A, et al. Safety and immediate humoral response of COVID-19 vaccines in chronic kidney disease patients: the SENCOVAC study. *Nephrology Dialysis Transplantation*. Published online November 12, 2021:gfab313. doi:10.1093/ndt/gfab313

61. Rabinowich L, Grupper A, Baruch R, et al. Low immunogenicity to SARS-CoV-2 vaccination among liver transplant recipients. *Journal of hepatology*. 2021;75(2):435-438. doi:10.1016/j.jhep.2021.04.020

© 2022 Manothummetha K et al. *JAMA Network Open.*

62. Rashidi-Alavijeh J, Frey A, Passenberg M, et al. Humoral Response to SARS-Cov-2 Vaccination in Liver Transplant Recipients-A Single-Center Experience. *Vaccines*. 2021;9(7). doi:10.3390/vaccines9070738

63. Rahav G, Lustig Y, Lavee J, et al. BNT162b2 mRNA COVID-19 vaccination in immunocompromised patients: A prospective cohort study. *EClinicalMedicine*. 2021;41:101158. doi:10.1016/j.eclinm.2021.101158

64. Rincon-Arevalo H, Choi M, Stefanski AL, et al. Impaired humoral immunity to SARS-CoV-2 BNT162b2 vaccine in kidney transplant recipients and dialysis patients. *Science immunology*. 2021;6(60). doi:10.1126/sciimmunol.abj1031

65. Rozen-Zvi B, Yahav D, Agur T, et al. Antibody response to SARS-CoV-2 mRNA vaccine among kidney transplant recipients: a prospective cohort study. *Clinical microbiology and infection : the official publication of the European Society of Clinical Microbiology and Infectious Diseases*. 2021;27(8):1173.e1-1173.e4. doi:10.1016/j.cmi.2021.04.028

66. Ruether DF, Schaub GM, Duengelhoef PM, et al. SARS-CoV2-specific Humoral and T-cell Immune Response After Second Vaccination in Liver Cirrhosis and Transplant Patients. *Clin Gastroenterol Hepatol*. Published online September 9, 2021:S1542-3565(21)00967-8. doi:10.1016/j.cgh.2021.09.003

67. Russo G, Lai Q, Poli L, et al. SARS-COV-2 VACCINATION WITH BNT162B2 IN RENAL TRANSPLANT PATIENTS: RISK FACTORS FOR IMPAIRED RESPONSE AND IMMUNOLOGICAL IMPLICATIONS. *Clin Transplant*. Published online September 26, 2021:e14495. doi:10.1111/ctr.14495

68. Eren Sadioğlu R, Demir E, Evren E, et al. Antibody response to two doses of inactivated SARS-CoV-2 vaccine (CoronaVac) in kidney transplant recipients. *Transpl Infect Dis*. Published online October 4, 2021:e13740. doi:10.1111/tid.13740

69. Sanders JSF, Bemelman FJ, Messchendorp AL, et al. The RECOVAC Immune-response Study: The Immunogenicity, Tolerability, and Safety of COVID-19 Vaccination in Patients With Chronic Kidney Disease, on Dialysis, or Living With a Kidney Transplant. *Transplantation*. Published online December 17, 2021. doi:10.1097/TP.0000000000003983

70. Sattler A, Schrezenmeier E, Weber UA, et al. Impaired humoral and cellular immunity after SARS-CoV-2 BNT162b2 (tozinameran) prime-boost vaccination in kidney transplant recipients. *The Journal of clinical investigation*. 2021;131(14). doi:10.1172/JCI150175

71. Schmidt T, Klemis V, Schub D, et al. Cellular immunity predominates over humoral immunity after homologous and heterologous mRNA and vector-based COVID-19 vaccine regimens in solid organ transplant recipients. *AMERICAN JOURNAL OF TRANSPLANTATION*. doi:10.1111/ajt.16818

© 2022 Manothummetha K et al. *JAMA Network Open.*

72. Schramm R, Costard-Jackle A, Rivinius R, et al. Poor humoral and T-cell response to two-dose SARS-CoV-2 messenger RNA vaccine BNT162b2 in cardiothoracic transplant recipients. *Clinical research in cardiology : official journal of the German Cardiac Society*. 2021;110(8):1142-1149. doi:10.1007/s00392-021-01880-5

73. Schrezenmeier E, Rincon-Arevalo H, Stefanski AL, et al. B and T Cell Responses after a Third Dose of SARS-CoV-2 Vaccine in Kidney Transplant Recipients. *JASN*. 2021;32(12):3027-3033. doi:10.1681/ASN.2021070966

74. Shostak Y, Shafran N, Heching M, et al. Early humoral response among lung transplant recipients vaccinated with BNT162b2 vaccine. *The Lancet Respiratory medicine*. 2021;9(6):e52-e53. doi:10.1016/S2213-2600(21)00184-3

75. Stumpf J, Tonnus W, Paliege A, et al. Cellular And Humoral Immune Responses after Three Doses of BNT162b2 mRNA SARS-Cov-2 Vaccine in Kidney Transplant. *Transplantation*. 2021;(wej, 0132144). doi:10.1097/TP.0000000000003903

76. Stumpf J, Siepmann T, Lindner T, et al. Humoral and cellular immunity to SARS-CoV-2 vaccination in renal transplant versus dialysis patients: A prospective, multicenter observational study using mRNA-1273 or BNT162b2 mRNA vaccine. *The Lancet regional health Europe*. 2021;(101777707):100178. doi:10.1016/j.lanepe.2021.100178

77. Thuluvath PJ, Robarts P, Chauhan M. Analysis of antibody responses after COVID-19 vaccination in liver transplant recipients and those with chronic liver diseases. *Journal of hepatology*. 2021;(ibs, 8503886). doi:10.1016/j.jhep.2021.08.008

78. Villanego F, Cazorla JM, Vigara LA, et al. Protecting kidney transplant recipients against SARS-CoV-2 infection: A third dose of vaccine is necessary now. *AMERICAN JOURNAL OF TRANSPLANTATION*. doi:10.1111/ajt.16829

79. Werbel WA, Boyarsky BJ, Ou MT, et al. Safety and Immunogenicity of a Third Dose of SARS-CoV-2 Vaccine in Solid Organ Transplant Recipients: A Case Series. *Annals of internal medicine*. 2021;(0372351). doi:10.7326/L21-0282

80. Westhoff TH, Seibert FS, Anft M, et al. A third vaccine dose substantially improves humoral and cellular SARS-CoV-2 immunity in renal transplant recipients with primary humoral nonresponse. *Kidney Int*. Published online September 9, 2021. doi:10.1016/j.kint.2021.09.001

81. Wijtvliet VPWM, Ariën KK, Abrams S, et al. mRNA-1273 vaccine (Moderna): a better option than BNT162b2 (Pfizer) in kidney transplant recipients and dialysis patients? *Nephrology Dialysis Transplantation*. Published online December 9, 2021:gfab352. doi:10.1093/ndt/gfab352

© 2022 Manothummetha K et al. *JAMA Network Open.*

82. Yi SG, Knight RJ, Graviss EA, et al. Kidney Transplant Recipients Rarely Show an Early Antibody Response Following the First COVID-19 Vaccine Administration. *Transplantation*. 2021;105(7):e72-e73. doi:10.1097/TP.0000000000003764

83. Cholankeril G, Al-Hillan A, Tarlow B, et al. Clinical Factors Associated With Lack of Serological Response to SARS-CoV-2 Messenger RNA Vaccine in Liver Transplantation Recipients. *Liver Transplantation*. 2022;28(1):123-126. doi:10.1002/lt.26351

© 2022 Manothummetha K et al. *JAMA Network Open.*
